# Assembling a Cinnamyl Pharmacophore in the C3-Position of Substituted Isatins via Microwave-Assisted Synthesis: Development of a New Class of Monoamine Oxidase-B Inhibitors for the Treatment of Parkinson’s Disease

**DOI:** 10.3390/molecules28166167

**Published:** 2023-08-21

**Authors:** Amritha Manoharan, Jong Min Oh, Feba Benny, Sunil Kumar, Mohamed A. Abdelgawad, Mohammed M. Ghoneim, Mohamed E. Shaker, Mohamed El-Sherbiny, Hailah M. Almohaimeed, Prashant Gahtori, Hoon Kim, Bijo Mathew

**Affiliations:** 1 Department of Pharmaceutical Chemistry, Amrita School of Pharmacy, Amrita Vishwa Vidyapeetham, AIMS Health Sciences Campus, Kochi 682 041, India; manoharanamritha99@gmail.com (A.M.); febacbenny@gmail.com (F.B.); solankimedchem@gmail.com (S.K.); 2 Department of Pharmacy, Research Institute of Life Pharmaceutical Sciences, Sunchon National University, Suncheon 57922, Republic of Korea; ddazzo005@naver.com; 3 Department of Pharmaceutical Chemistry, College of Pharmacy, Jouf University, Sakaka 72341, Saudi Arabia; mhmdgwd@ju.edu.sa; 4 Pharmaceutical Organic Chemistry Department, Faculty of Pharmacy, Beni-Suef University, Beni-Suef 62514, Egypt; 5 Department of Pharmacy Practice, College of Pharmacy, AlMaarefa University, Ad Diriyah 13713, Saudi Arabia; mghoneim@um.edu.sa; 6 Pharmacognosy and Medicinal Plants Department, Faculty of Pharmacy, Al-Azhar University, Cairo 11884, Egypt; 7 Department of Pharmacology, College of Pharmacy, Jouf University, Sakaka 72341, Saudi Arabia; melsayed@ju.edu.sa; 8 Department of Pharmacology & Toxicology, Faculty of Pharmacy, Mansoura University, Mansoura 35516, Egypt; 9 Department of Basic Medical Sciences, College of Medicine, AlMaarefa University, Riyadh 11597, Saudi Arabia; msharbini@mcst.edu.sa; 10 Department of Anatomy, Faculty of Medicine, Mansoura University, Mansoura 35516, Egypt; 11 Department of Basic Science, College of Medicine, Princess Nourah bint Abdulrahman University, Riyadh 11671, Saudi Arabia; hmalmohaimeed@pnu.edu.sa; 12 School of Pharmacy, Graphic Era Hill University, Dehradun 248002, India; pgahtori@gehu.ac.in

**Keywords:** indole-based phenylallylidene derivatives, monoamine oxidase inhibitors, kinetics, reversibility, neuroprotection, molecular docking, neurological diseases

## Abstract

Monoamine oxidase (MAO, EC 1.4.3.4) is responsible for the oxidative breakdown of both endogenous and exogenous amines and exists in MAO-A and MAO-B isomers. Eighteen indole-based phenylallylidene derivatives were synthesized via nucleophilic addition reactions comprising three sub-series, **IHC**, **IHMC**, and **IHNC**, and were developed and examined for their ability to inhibit MAO. Among them, compound **IHC3** showed a strong MAO-B inhibitory effect with an IC_50_ (*half-maximal inhibitory concentration*) value of 1.672 μM, followed by **IHC2** (IC_50_ = 16.934 μM). Additionally, **IHC3** showed the highest selectivity index (SI) value of >23.92. The effectiveness of **IHC3** was lower than the reference pargyline (0.14 μM); however, the SI value was higher than pargyline (17.16). Structurally, the **IHC** (-H in the B-ring) sub-series exhibited relatively stronger MAO-B inhibition than the others. In the **IHC** series, **IHC3** (-F in the A-ring) exhibited stronger MAO-B suppression than the other substituted derivatives in the order -F > -Br > -Cl > -OCH_3_, -CH_3_, and -H at the 2-position in the A-ring. In the reversibility and enzyme kinetics experiments, **IHC3** was a reversible inhibitor with a K_i_ value of 0.51 ± 0.15 μM for MAO-B. Further, it was observed that **IHC3** greatly decreased the cell death caused by rotenone in SH-SY5Y neuroblastoma cells. A molecular docking study of the lead molecule was also performed to determine hypothetical interactions in the enzyme-binding cavity. These findings suggest that **IHC3** is a strong, specific, and reversible MAO-B inhibitor that can be used to treat neurological diseases.

## 1. Introduction

Isatin (1H-indole-2,3-dione), often referred to as indole quinone or indenedione, is a flexible structural motif that can be employed to synthesize a wide range of heterocyclic compounds [1]. It is also referred to as an endogenous indole that is present in the peripheral tissues and brain of mammals [2] It was primarily isolated from the *Couroupita guianesis* and Isatis genus of plants [3]. Other plants, such as *Melochia tomentosa* can also be used to obtain substituted isatins, the melosatin alkaloids, that contain methoxy phenylpentyl isatins. In addition to plants, fungi such as *Streptomyces albus* and *Chaetomium globosum* were employed to isolate the isatins 6-(3’-methylbuten-2’-yl) and 5-(3’-methylbuten-2’-yl), respectively [4,5] Isatin was originally synthesized by Erdmann and Laurent as a byproduct of indigo dye oxidation using nitric and chromic acids. Isatin forms orange-red monoclinic prism crystals that melt at 200 °C when dissolved in water, acetic acid, or alcohol(ethanol) [6]. The most prevalent methods to produce isatin are the Sandmeyer reaction, Martinet synthesis, Stolle method, and Gassman synthesis. The metalation of anilide derivatives is also a contemporary approach to the synthesis of isatin [4,7]. Isatin contains a bicyclic heteroaromatic nucleus in which the six-membered ring is carbocyclic and the five-membered ring is heterocyclic, with di-keto functional groups at the C-2 and C-3 positions. Both rings are on the same plane. The five-membered ring is anti-aromatic, whereas the six-membered ring is aromatic [6,8]. The isatin molecule undergoes numerous structural modifications owing to its association with the phenyl ring, a γ-lactam moiety, and a carbonyl group. Isatins have been exploited as electrophiles and nucleophiles in various chemical reactions. The most recognized reactions in isatin include nucleophilic addition to the carbonyl group at the C-3 position and electrophilic substitution in the aromatic ring. Aldol condensation, oxidation, Friedel-Crafts reactions, and ring expansion reactions are significant chemical reactions of isatin at the C-3 position. A C-3 substituted isatin can be used to prepare a variety of pharmacologically active structural moieties, including hydrazones, imines, oxindoles, and thiosemicarbazones. Several studies have been conducted on the synthesis of spiro isatin compounds holding the C-3 position [6,9,10,11,12,13,14]. Isatin exhibits different types of biological activities, including CNS depressant, anticonvulsant, antioxidant, anti-inflammatory, antimalarial, anticancer, antiulcer, antitubercular, anti-HIV, and antibacterial activities [4,15,16,17].

Parkinson’s disease (PD) is the second most common neurological illness and usually affects elderly individuals worldwide. It currently impacts 0.3% of the population as a whole and 1–3% of people older than 65, and by the year 2030, its prevalence will increase from 8.7 to 9.3 million [18]. PD occurs through a pathological buildup of α-synuclein in Lewy bodies and a specific reduction of dopamine-producing neurons in the pars compacta of the substantia nigra, but there is still no recognized etiology for PD [19,20]. Monoamine oxidases (both isomers MAO-A and MAO-B; EC 1.4.3.4) are responsible for the oxidative breakdown of both endogenous and exogenous amines. Since the human brain’s MAO-B predominantly deaminates dopamine, PD patients ought to notice a rise in basal central levels of dopamine, when receiving MAO-B blockers. Consequently, this theory was used to design the MAO-B blocker as a PD medication [21]. MAO-B inhibitors became an effective treatment for PD in the early 1960s [22]. MAO is potently inhibited by isatin, which exhibits an IC_50_ value of roughly 3 µM; isatin appears to be more specific for MAO-B than MAO-A [23]. Isatin is located near the FAD (flavin adenine dinucleotide) cofactor in the MAO-B substrate cavity. The entrance cavity of the enzyme is free because isatin interacts with the substrate cavity. Thus, the C-5 or C-6 position of isatin can be replaced by a functional group that may occupy the entrance cavity and facilitate more effective interaction with the enzyme than isatin alone, possibly encouraging stronger suppression [24]. Several studies have generated various isatin derivatives with substitutions at the C-3, C-5, and C-6 positions and examined their capacity to inhibit MAO. A previous study revealed the MAO-inhibitory action of 3-hydroxy-3-phenacyloxindole analogs of isatin. A benzyl group at the first position and a bromoaryl or hydroxyaryl substitution at the third position of the isatin scaffold boosted its inhibitory action against MAO enzymes [25]. The findings from another study revealed that the most prevalent approach for increasing the MAO-B inhibitory potency of isatin is substitution at C-5 with various substituents [26]. Studies have also focused on incorporating different structural moieties at various positions in isatin. For instance, a strong MAO inhibitory action was observed when 3,4-(methylenedioxy)aniline was incorporated into isatin at the C-3 position via a semicarbazone linker. It turned out that the 5-chlorosubstituted derivative of isatin was more effective against MAO-B (IC_50_ = 7.253 ± 0.002 µM), whereas the unsubstituted derivative of isatin was more effective against MAO-A (IC_50_ = 3.26 ± 0.031 µM) [27]. Introducing 2-amino-6-nitrobenzothiazole at the C-3 position of isatin using a semicarbazone linker with a methylene spacer enhanced suppression and specificity towards MAO-B over MAO-A. According to this study, chlorine substitution at the fifth position of isatin was very effective against MAO-B (IC_50_ = 1.8 ± 0.3 nM), followed by its unsubstituted counterpart. However, replacing chlorine with nitrogen reduced the MAO inhibitory activity of the molecule [28]. Isatin has also been linked to other structural motifs using hydrazone linkers. Hydrazone was used as the linker to join piperonylic acid at the C-3 position of isatin. When *p*-fluoro or dichlorobenzyl groups were substituted at the N1 position, the resulting isatin derivatives exhibited stronger inhibition of MAO-B (IC_50_ = 0.52 ± 0.093 µM and IC_50_ = 0.64 ± 0.072 µM, respectively); however, the MAO-B inhibition ability is less compared to the substitution of a propargyl unit at the first position (IC_50_ = 0.034 ± 0.007 µM) [29].

Cinnamon, a member of the *Lauraceae* family, is a prominent herb used in both conventional and contemporary medicine. It is derived from the outer layer of bark of trees, belonging to the genera *Cinnamomum zeylanicum*/*Cinnamon cassia*. Cinnamaldehyde, trans-cinnamaldehyde, and cinnamic acid are the main components of cinnamon [30,31]. According to in vitro research on the impact of cinnamon and its metabolites in PD, the primary compounds of Cinnamomum species, particularly cinnamaldehyde and sodium benzoate, may have a neuroprotective effect by preventing oxidative damage by inducing cell death, producing reactive oxygen species (ROS), and improperly regulating of autophagy. Sodium benzoate is a product of cinnamic acid metabolism that increases the generation of neurotrophic factors and prevents neurological inflammation in PD [32,33]. The hindering of dysfunctional autophagy by cinnamaldehyde has been studied using 1-methyl-4-phenyl-1,2,3,6-tetrahydropyridine (MPTP) and 1-methyl-4-phenylpyridinium (MPP^+^), which are used to induce PD in animal and cellular models, respectively. The outcome showed that cinnamaldehyde dramatically decreased the rate of dopaminergic cell death in the substantia nigra and striatum of MPTP-treated mice. Additionally, autophagy suppression decreased MPP^+^-mediated cell death [34]. The MAO inhibitory activity of cinnamaldehyde and its derivatives has also been explored, showing high potency against MAO-B [35].

Molecular hybridization is an innovative approach in medicinal chemistry wherein two pharmacophoric units can be joined to create a novel compound with enhanced effectiveness compared to the parental compounds. The development and functionality of these compounds depend on the pharmacophoric units and the types of linkers employed to join them [36,37]. Their enhanced flexibility, the existence of a donor atom (nitrogen), and the ease of synthesizing hydrazone make them an appealing research topic in coordination chemistry [38,39].

A well-known endogenous small molecule MAO-B inhibitor is indole-2,3-dione. In the review and computational work, our group addressed how the C-3 position of indole-2,3-dione is not utilized often [9,40]. As previously mentioned, cinnamon has neuroprotective properties. Based on this observation, we designed indole-based phenylallylidene derivatives by incorporating cinnamaldehyde at the C-3 position of isatin by using a hydrazone linker, since hydrazone can easily attack the carbonyl carbon of cinnamaldehyde. In the current investigation, phenylallylidene derivatives based on indoles were synthesized and tested for MAO inhibitory activity. The lead compound was evaluated for kinetics, reversibility, neuroprotection, and computational studies.

## 2. Results and Discussion

### 2.1. Chemistry

Indole-based phenylallylidene derivatives were synthesized (Figure 1) via a nucleophilic addition reaction between the differently substituted isatin hydrazones and para-substituted cinnamaldehydes. Eighteen derivatives were synthesized and characterized using ^1^H NMR, ^13^C NMR, and mass spectroscopy (Supplementary Materials, Figures S1–S18).

### 2.2. Biochemistry

#### 2.2.1. Inhibition Studies of MAO-A and MAO-B

**Figure 2:**
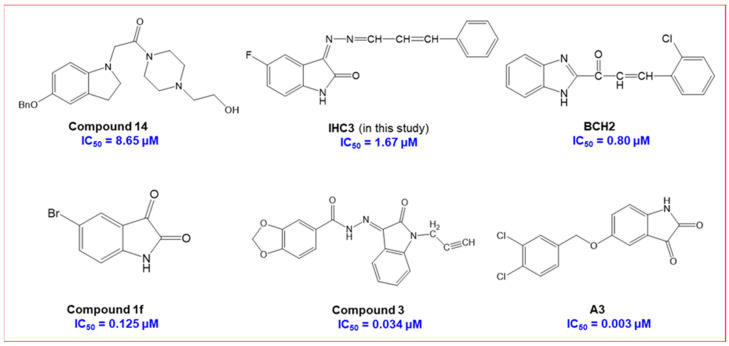
Structures of lead compounds as MAO-B inhibitor, compared to **IHC3**. References cited were represented. **Compound 14** [14], **BCH2** [42], **Compound 1f** [43], **Compound 3** [29] and **A3** [44].

**Figure 3:**
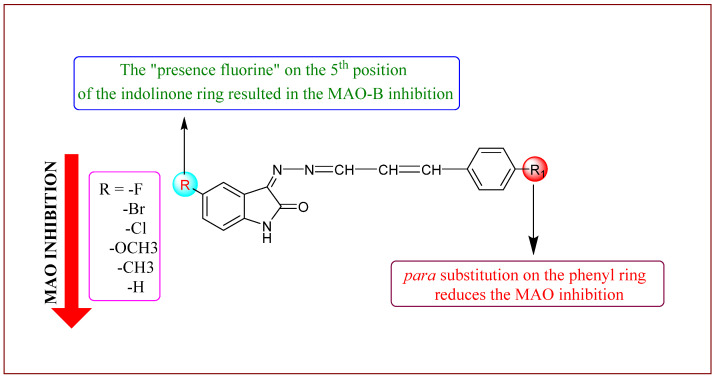
Structure-activity relationship of indole-based phenylallylidene derivatives.

The full series comprised 18 compounds from three sub-series: the basic series, **IHC**, and two sub-series, **IHMC** and **IHNC**. Among them, compound **IHC3** showed less than 50% residual activity against MAO-B at a concentration of 10 µM, with an IC_50_ value of 1.672 μM, followed by **IHC2** (IC_50_ = 16.934 μM) (Table 1). The IC_50_ value of **IHC3** was lower than the indol and 1-(3-(benzyloxy)benzyl)piperazine **14** (IC_50_ = 8.65 μM)[41], but higher than the benzimidazole chalcone derivative **BCH2** (IC_50_ = 0.80 μM) [42], isatin analog **1f** (IC_50_ = 0.125 μM) [43], piperonylic acid-derived hydrazone bearing isatin **3** (IC_50_ = 0.034 μM) [29] and isatin derivative **A3** (IC_50_ = 0.003 μM) [44] (Figure 2). In addition, **IHC3** had the highest selectivity index (SI) value (> 23.92), indicating that it is a selective MAO-B inhibitor (Table 1). Structurally, most compounds in the **IHC** series (-H in the B-ring) showed stronger MAO-B suppression than the other sub-series of compounds, **IHMC** (-OCH_3_ in the B-ring), and **IHNC** (-NO_2_ in the B-ring). In the **IHC** series, **IHC**3 (-F in A-ring) had 10.13 to 23.92 times higher MAO-B inhibition than the other substituted derivatives, in the order -F > -Br > -Cl > -OCH_3_, -CH_3_, and -H at the 2-position in the A-ring (Figure 3). In addition, **IHC**3 showed 15.07- and 13.15-times higher MAO-B inhibition than other sub-series compounds, such as **IHMC2** (-Br in the A-ring, IC_50_ = 25.192 μM) and **IHNC2** (-Br in A-ring, IC_50_ = 21.995 μM), respectively, which showed the highest MAO-B inhibition in the other two sub-series. In contrast, all compounds showed weak MAO-A inhibition. These findings indicated that **IHC3** is a strong and specific MAO-B inhibitor.

#### 2.2.2. Reversibility Studies

**Figure 4:**
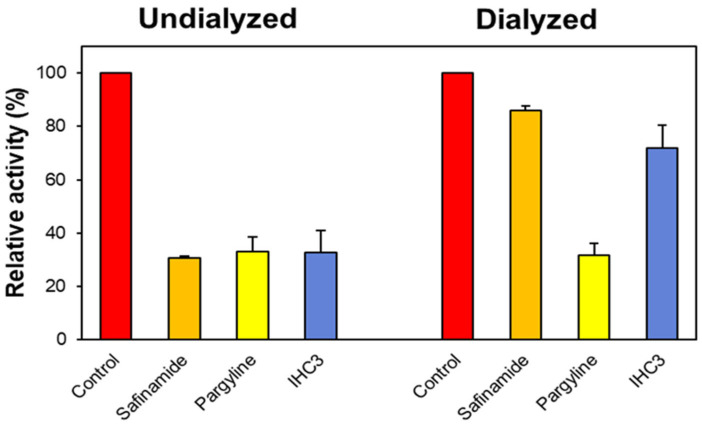
Recovery of MAO-B inhibition by **IHC3** using dialysis experiments. The concentrations of safinamide (reversible MAO-B inhibitor), pargyline (an irreversible MAO-B inhibitor), and **IHC3** used were ~2.0 times their IC_50_ values (0.04, 0.28, and 3.20 μM, respectively). After preincubation for 30 min, the mixtures were dialyzed for 6 h, with two buffer changes.

Dialysis was performed to assess whether the inhibitory effect of **IHC3** on MAO-B was reversible. The total concentrations of safinamide (a reversible MAO-B inhibitor), pargyline (an irreversible MAO-B inhibitor), and **IHC3** utilized in this study were approximately 2.0 times higher than their respective IC_50_ values (0.04, 0.28, and 3.20 M, respectively). Recovery trends were determined by comparing the relative A_U_ and A_D_ activities. The residual activity of **IHC3** was recovered from 32.87% (A_U_) to 72.05% (A_D_) (Figure 4), which was close to that of safinamide (A_U_ and A_D_ of 30.65% and 85.86%, respectively) but not pargyline (A_U_ and A_D_ of 32.86% and 31.53%, respectively). These findings suggest that **IHC3** is a reversible MAO-B inhibitor.

#### 2.2.3. Enzyme Kinetics

**Figure 5:**
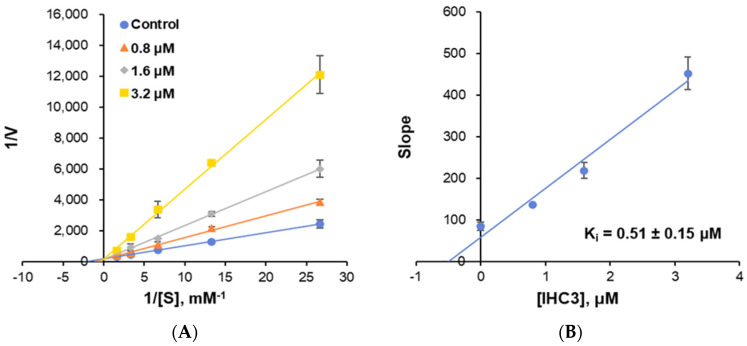
Lineweaver-Burk plots for the MAO-B inhibition activity of **IHC3** (**A**) and its secondary plot (**B**) of the slopes vs. inhibitor concentrations. The experiments were analyzed at five concentrations of benzylamine as a substrate and three inhibitor concentrations.

The enzyme kinetics of MAO-B using **IHC3** were examined at three different inhibitor doses and five different benzylamine concentrations. The Lineweaver-Burk plot (Figure 5A) suggests that **IHC3** is a competitive MAO-B inhibitor. K_m_ and V_max_ values were 0.46 ± 0.06 mM and 0.0054 ± 0.0001 ΔA/min, respectively (Figure 5A and Figure S19A). In comparison to the nonlinear regression method, a hyperbola curve was analyzed [45]. Those values were 0.41 ± 0.06 mM and 0.0050 ± 0.0004 ΔA/min, respectively (Figure S19B), determined by GraphPad Prism software 5. Therefore, the conditions, i.e., ~ 1/4×, 1/2×, 1×, 2×, and 4× K_m_ used in this study were relevant to the basic kinetics. The secondary plot (Figure 5B) shows that the Ki value of the reaction was 0.51 ± 0.15 M. The inhibitor concentrations used were ~ 1/2×, ~1×, and ~2× IC_50_ values. Based on these findings, we concluded that **IHC3** is a potent competitive MAO-B inhibitor.

#### 2.2.4. Neuroprotection Studies

**Figure 6:**
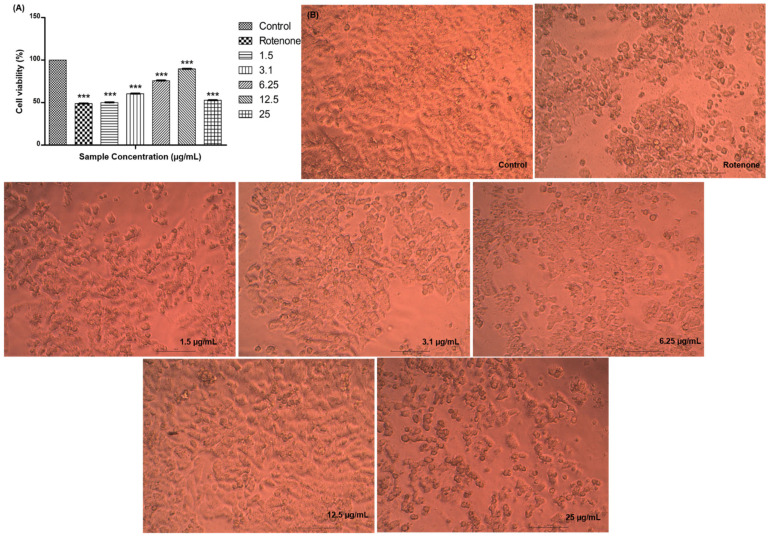
(**A**) Graphical representation depicting the neuroprotective effect of **IHC3** on rotenone-induced SHSY5Y cells by MTT assay. *Y*-axis, percentage viability; *X*-axis, concentration of **IHC3**. All experiments were done in triplicates and results represented as mean ± SE. One-way ANOVA and Dunnets test were performed to analyze data. *** *p* < 0.001 compared to control groups. (**B**) Rotenone-induced morphological aberration in SH-SY5Y cells.

Using the MTT assay test, the neuroprotective effects of rotenone-induced on SH-SY5Y cells were evaluated. **IHC3** increased cell viability dose-dependently with the maximum at 89.67% at 12.5 µg/mL, with treated to rotenone-induced cells (Figure 6A). However, the viability decreased at a concentration of 25 µg/mL as the neuroprotective effect of the compound was significantly diminished. Phase-contrast microscopy was also used to examine the compound’s impact on cellular morphology (Figure 6B). The integrity of the cell membrane and reduction in cell number, relating to cellular viability, were morphologically shown in the SH-SY5Y cells.

### 2.3. Molecular Docking

**Figure 7:**
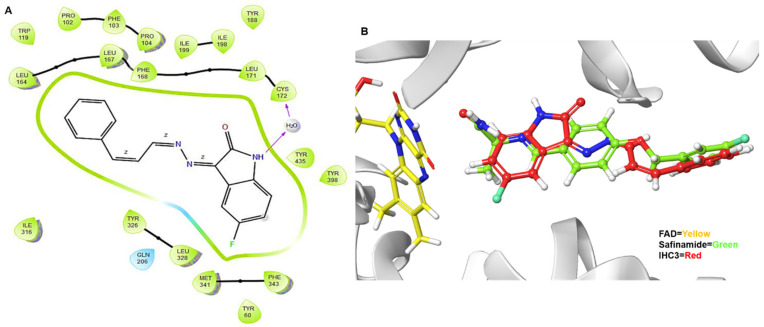
(**A**) Binding interactions of the compound **IHC3** to the active site of the hMAO-B enzyme (PDB ID: 2V5Z). (**B**) 3D binding poses of FAD (yellow), safinamide (green), and **IHC3** (red).

Based on the outcomes of the enzyme evaluation, the results showed that **IHC3** was the best compound in the series and was evaluated against hMAO-B enzymes. Docking analyses were performed to investigate the binding mechanisms of **IHC3** and evaluate the effects of structure-related alterations on its inhibitory action. The X-ray crystal structure of hMAO-B was taken from the Protein Data Bank (PDB ID: 2V5Z). **IHC3** had high docking scores for hMAO-B and a docking score of −11.061 kcal (XP GlideScore) for MAO-B (Table 2). The hMAO-B docking pose demonstrated a water molecule serving as a connecting medium to link the NH group of isatin with the amino acid Cys172 of MAO-B through a hydrogen bond. Meanwhile, the side chains of Tyr435, Phe343, Tyr398, Met341, Leu328, Tyr326, Ile316, Leu167, Phe168, Leu164, and Leu171 were connected via hydrophobic interactions with **IHC3**, while Gln206 was connected via polar interactions (Figure 7A). It was hypothesized that this interaction was essential for **IHC3** and would clarify its enhanced suppressive effect because neither of the remaining compounds showed an equivalent interaction. To confirm the docking findings, we re-docked a native ligand in the MAO-B pocket. It was revealed that the amide side chain and isatin scaffold of safinamide or **IHC3** bind closed to the flavin adenine dinucleotide (FAD) (Figure 7B). Therefore, the similar binding orientation like safinamide and -NH of isatin with Cys172 may engage in significant hydrogen-bonding interactions, which may have a major impact on the biological function of isatin.

## 3. Materials and Methods

### 3.1. Synthetic Strategy

Here, 2.5:1 mixtures of hydrazine hydrate and differently substituted isatins were dissolved in 7.5 mL of methanol and glacial acetic acid (as the catalyst), producing the corresponding isatin hydrazones. Subsequently, 1:1 mixtures of the intermediate and differently substituted cinnamaldehyde were dissolved in methanol (7.5 mL), and 2–3 drops of glacial acetic acid (catalyst) were added to this mixture. The mixture was set up in a microwave reactor (Monowave 50, Anton Paar, Graz, Austria) for 7 min at 120 °C to complete the reaction. Thin-layer chromatography (TLC) was performed using a mixture of 2:1 hexane and ethyl acetate to observe the reaction. Finally, the product was washed with methanol, filtered, and the air-dried product was recrystallized from methanol.

### 3.2. Biochemistry

#### 3.2.1. Inhibition Studies of MAO-A and MAO-B

The activity of MAO-A and MAO-B was assessed using 0.06 mM kynuramine and 0.3 mM benzylamine, respectively [46], and the absorbance was determined using a continuous assay technique [47]. The activity at various doses of the compounds was assessed and the IC_50_ value for the compound exhibiting a residual activity of less than 80% was determined by using GraphPad Prism software 5 to a limit of 40 µM. The inhibitory effects of the compounds produced were compared with standard MAO-A (clorgyline and toloxatone) and MAO-B (pargyline and safinamide) inhibitors. The SI values were computed using the following formula: (IC_50_ of MAO-A)/(IC_50_ of MAO-B) [46,47,48].

#### 3.2.2. Reversibility Studies

By comparing the dialyzed and undialyzed residual activities of the lead compound at a concentration of 2.0 times the IC_50_ value and incubating them for 30 min before evaluation, as previously reported [47], the reversibility of the MAO-B inhibition activity by the lead compounds was examined. After preincubation, the mixture was dialyzed for 6 h with two buffer changes at 4 °C. Safinamide and pargyline, the standard reversible and irreversible MAO-B inhibitors, respectively, were used to compare the regained activity of the compounds. Reversibility was determined by comparing the activities of the dialyzed (A_D_) and undialyzed (A_U_) samples [47].

#### 3.2.3. Enzyme Kinetics

Five substrate concentrations (0.0375–0.60 mM) of benzylamine for MAO-B [46,48] and three inhibitor concentrations (approximately 0.5, 1.0, and 2.0 times the IC_50_ value) [46] were used to assess the inhibitory activity of the lead compounds for MAO-B. Analysis of the resulting Lineweaver–Burk plot and its secondary plot revealed the enzyme inhibition profile and K_i_ value, respectively [46]. To compare the data with the nonlinear regression method, a hyperbola curve was analyzed [45].

#### 3.2.4. Neuroprotection Studies

The neuroprotective effects of **IHC3** on rotenone-induced SH-SY5Y cells were assessed using the MTT test. In 96-well plates, SH-SY5Y cells (5 × 10^4^ cells/well) were cultivated. Rotenone (10 µM) was pre-treated within different concentrations on cells in the presence or absence of **IHC3** (1.5, 3.1, 6.25, 12.5, and 25 µg in 500 µL of 5% DMEM). The sample material in the wells was removed after the 24 h incubation period and 30 µL of reconstituted MTT solution was added. Then it was incubated for 4 h at 37 °C in humidified 5% CO_2_. The formazan crystals were dissolved by adding 100 µL of MTT solubilization solution, after the supernatant had been taken from the sample during the incubation period. At a wavelength of 540 nm, the absorbance measurements were obtained using a microplate reader (Erba Lisa Scan EM, Mannheim, Germany).

### 3.3. Molecular Docking

The binding potential of the lead compound to the binding pockets of human MAO-B (PDB ID: 2V5Z) was determined using an in silico method using Schrodinger Maestro (v13.4). Preprocessing, optimization, and protein energy minimization were performed using protein preparation wizard (PPW) program to generate the protein crystal structures. LigPrep was used for ligand preparation for the docking study. A receptor grid-generating module and grid file were created at the exact location of the co-crystallized ligand. Ligand docking was performed in extra precision (XP) mode.

## 4. Conclusions

In conclusion, eighteen isatin derivatives were synthesized, and their inhibitory activities against MAO were evaluated. Of these, **IHC3** was a competitive and reversible MAO-B inhibitor with an IC_50_ value of 1.672 ± 0.022 µM, and **IHC2** was found to be the second active molecule. There was a speck of unequivocal evidence for the inhibitory activity of the fluorine-substituted molecule. Eventually, it was observed that **IHC3** greatly decreased the cell death caused by rotenone in SH-SY5Y neuroblastoma cells. Computational studies showed that the NH group of isatin could interact with the amino acid Cys172 of MAO-B through a hydrogen bond and that a water molecule was a bridge for this linkage. This may be the major reason for the effective inhibition of hMAO-B by compound **IHC3**, which showed a docking score of −11.061 kcal/mol. Collectively, the results revealed that incorporating an electronegative atom at the C-5 position of the indolinone ring enhanced MAO-B inhibition.

## Figures and Tables

**Figure 1:**
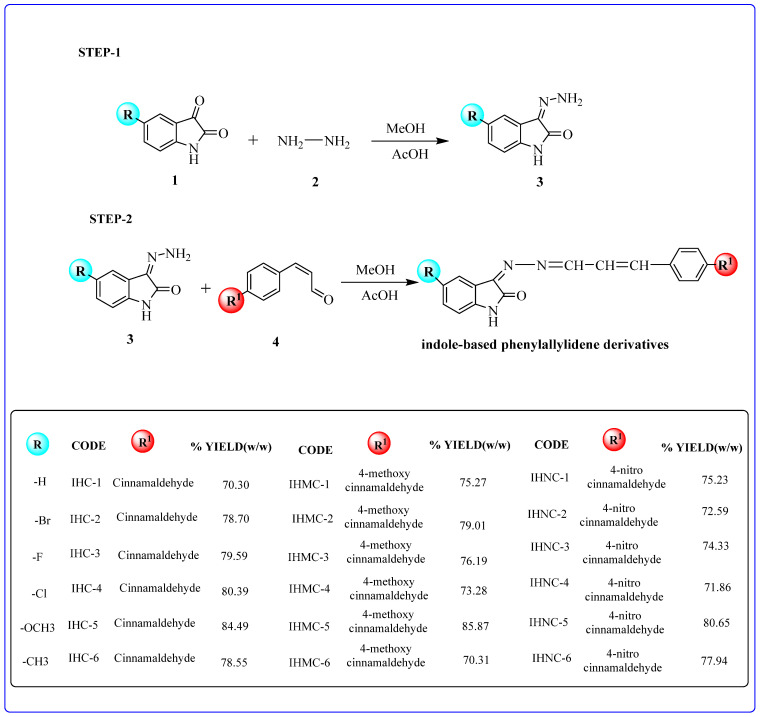
Synthesis of indole-based phenylallylidene derivatives. **1**, substituted isatin; **2**, hydrazine; **3**, isatin-hydrazide; **4**, substituted cinnamaldehyde.

**Table 1:** Inhibitory activity of the 18 compounds of the **IHC** series against MAO-A and MAO-B ^a^.

Compounds	Residual Activity at 10 µM (%)		IC_50_ (µM)		SI ^b^
	MAO-A	MAO-B	MAO-A	MAO-B	
**IHC1**	94.48 ± 2.10	89.30 ± 3.00	>40	>40	
**IHC2**	92.37 ± 0.52	61.39 ± 3.42	>40	16.934 ± 0.397	>2.36
**IHC3**	76.90 ± 1.55	11.39 ± 5.22	>40	1.672 ± 0.022	>23.92
**IHC4**	86.74 ± 1.78	73.89 ± 8.87	>40	27.485 ± 1.152	>1.46
**IHC5**	82.27 ± 1.80	75.79 ± 6.17	>40	>40	
**IHC6**	98.10 ± 1.55	79.44 ± 3.25	>40	>40	
**IHMC1**	89.92 ± 1.30	96.89 ± 0.95	>40	>40	
**IHMC2**	86.63 ± 0.52	78.07 ± 1.12	>40	25.192 ± 0.147	>1.59
**IHMC3**	85.54 ± 6.42	81.29 ± 4.61	>40	>40	
**IHMC4**	81.46 ± 0.77	76.55 ± 3.27	>40	>40	
**IHMC5**	84.48 ± 0.73	85.60 ± 4.27	>40	>40	
**IHMC6**	89.80 ± 2.55	91.85 ± 3.48	>40	>40	
**IHNC1**	88.70 ± 2.40	96.91 ± 3.36	> 40	>40	
**IHNC2**	88.50 ± 0.71	71.25 ± 1.99	>40	21.995 ± 0.354	>1.82
**IHNC3**	80.40 ± 0.57	78.93 ± 3.35	>40	>40	
**IHNC4**	82.30 ± 0.99	74.50 ± 4.63	>40	26.486 ± 0.600	>1.51
**IHNC5**	88.60 ± 1.98	81.99 ± 1.05	>40	>40	
**IHNC6**	87.20 ± 1.13	81.09 ± 6.40	>40	>40	
Toloxatone			1.08 ± 0.03	>40	<0.027
Safinamide			>40	0.021 ± 0.001	>1904
Clorgyline			0.007 ± 0.001	1.853 ± 0.112	0.004
Pargyline			2.403 ± 0.358	0.14 ± 0.01	17.16

^a^ Results represent the means ± standard errors of duplicate or triplicate experiments. ^b^ Selectivity index (SI) = IC_50_ for MAO-A/IC_50_ for MAO-B.

**Table 2:** Docking scores of **IHC3** to the hMAO-B active site.

Compound	Docking Score (kcal)
**IHC3**	−11.061
Safinamide	−11.136

The PDB ID of MAO-B is 2V5Z.

## Data Availability

Not applicable.

## Supplementary Materials

The following supporting information can be downloaded at: https://www.mdpi.com/article/10.3390/molecules28166167/s1, Figures S1–S18: Structures of sixteen compounds comprising TLC, ^1^H-NMR spectrum, ^13^C-NMR spectrum, Mass spectrum, and spectral interpretation. Figure S19: MAO-B kinetics.
