# Supplementary material for: Assembling a Cinnamyl Pharmacophore in the C3-Position of Substituted Isatins via Microwave-Assisted Synthesis: Development of a New Class of Monoamine Oxidase-B Inhibitors for the Treatment of Parkinson’s Disease
Source: Molecules. 2023 Aug 21;28(16):6167. doi: 10.3390/molecules28166167 (PMC10458360; doi:10.3390/molecules28166167)
Supplement: Supplementary file 1 [file molecules-28-06167-s001.zip › molecules-2539671-supplementary.pdf]

Supporting Information

Assembling a cinnamyl pharmacophore in the C3-position of substituted isatins via microwave-assisted synthesis: Development of a new class of monoamine oxidase-B inhibitors for the treatment of Parkinson's disease

Amritha Manoharan [1,#], Jong Min Oh [2,#], Feba Benny [1], Sunil Kumar [1], Mohamed A. Abdelgawad [3,4], Mohammed M. Ghoneim [5,6], Mohamed E. Shaker [7,8], Mohamed El-Sherbiny [9,10], Hailah M. Almohaimeed [11], Prashant Gahtori [12], Hoon Kim [2,*] and Bijo Mathew [1,*]

[1] Department of Pharmaceutical Chemistry, Amrita School of Pharmacy, Amrita Vishwa Vidyapeetham, AIMS Health Sciences Campus, Kochi, 682 041 India; manoharanamritha99@gmail.com (A.M.); febacbenny@gmail.com (F.B.); solankimedchem@gmail.com (S.K.)

[2] Department of Pharmacy, and Research Institute of Life Pharmaceutical Sciences, Sunchon National University, Suncheon 57922, Republic of Korea ; ddazzo005@naver.com (J.M.O.)

[3] Department of Pharmaceutical Chemistry, College of Pharmacy, Jouf University, Sakaka 72341, Saudi Arabia; mhmdgwd@ju.edu.sa (M.A.A)

[4] Pharmaceutical Organic Chemistry Department, Faculty of Pharmacy, Beni-Suef University, Beni suef 62514, Egypt

[5] Department of Pharmacy Practice, College of Pharmacy, AlMaarefa University, Ad Diriyah 13713, Saudi Arabia; mghoneim@um.edu.sa (M.M.G.)

[6] Pharmacognosy and Medicinal Plants Department, Faculty of Pharmacy, Al-Azhar University, Cairo 11884, Egypt

[7] Department of Pharmacology, College of Pharmacy, Jouf University, Sakaka 72341, Aljouf, Saudi Arabia; melsayed@ju.edu.sa (M.E.S.)

[8] Department of Pharmacology & Toxicology, Faculty of Pharmacy, Mansoura University, Mansoura 35516, Egypt

[9] Department of Basic Medical Sciences, College of Medicine, AlMaarefa University, P.O. Box 71666, Riyadh 11597, Saudi Arabia; msharbini@mcst.edu.sa (M.E.-S.)

[10] Department of Anatomy, Faculty of Medicine, Mansoura University, Mansoura 35516, Egypt

[11] Department of Basic Science , College of Medicine ,Princess Nourah bint Abdulrahman University ,P.O.Box 84428, Riyadh 11671, Saudi Arabia; hmalmohaimeed@pnu.edu.sa (H.M.A)

[12] School of Pharmacy, Graphic Era Hill University, Dehradun 248002, Uttarakhand, India; pgahtori@gehu.ac.in (P.G.)

[#]Authors contributed equally to this work.

* Correspondence: bijomathew@aims.amrita.edu or bijovilaventgu@gmail.com (B.M.); hoon@sunchon.ac.kr (H.K.)

Table of Contents

Figure S1. IHC-1 Structure………………………………………………………………… S1
1. IHC-1
1.1 TLC of IHC-1
1.2. ^{1}H-NMR of IHC-1
1.3. ^{13}C-NMR of IHC-1
1.4. Mass Spectrum of IHC-1
1.5. Spectral interpretation of IHC-1

Figure S2. IHC-2 Structure………………………………………………………………… S3
2. IHC-2 structure
2.1 TLC of IHC-2
2.2. ^{1}H-NMR of IHC-2
2.3. ^{13}C-NMR of IHC-2
2.4. Mass Spectrum of IHC-2
2.5. Spectral interpretation of IHC-2

Figure S3. IHC-3 Structure………………………………………………………………… S5
3. IHC-3 structure
3.1 TLC of IHC-3
3.2. ^{1}H-NMR of IHC-3
3.3. ^{13}C-NMR of IHC-3
3.4. Mass Spectrum of IHC-3
3.5. Spectral interpretation of IHC-3

Figure S4. IHC-4 Structure………………………………………………………………… S7
4. IHC-4 structure
4.1 TLC of IHC-4
4.2. ^{1}H-NMR of IHC-4
4.3. ^{13}C-NMR of IHC-4
4.4. Mass Spectrum of IHC-4
4.5. Spectral interpretation of IHC-4

Figure S5. IHC-5 Structure………………………………………………………………… S9
5. IHC-5 structure
5.1 TLC of IHC-5
5.2. ^{1}H-NMR of IHC-5
5.3. ^{13}C-NMR of IHC-5
5.4. Mass Spectrum of IHC-5
5.5. Spectral interpretation of IHC-5

Figure S6. IHC-6 Structure………………………………………………………………. S11
6. IHC-6 structure
6.1 TLC of IHC-6
6.2. ^{1}H-NMR of IHC-6
6.3. ^{13}C-NMR of IHC-6
6.4. Mass Spectrum of IHC-6
6.5. Spectral interpretation of IHC-6

Figure S7. IHMC-1 Structure…………………………………………………………… S13
7. IHMC-1 structure
7.1 TLC of IHMC-1
7.2. ^{1}H-NMR of IHMC-1
7.3. ^{13}C-NMR of IHMC-1
7.4. Mass Spectrum of IHMC-1
7.5. Spectral interpretation of IHMC-1

Figure S8. IHMC-2 Structure…………………………………………………………………… S15
8. IHMC-2 structure
8.1 TLC of IHMC-2
8.2. ^{1}H-NMR of IHMC-2
8.3. ^{13}C-NMR of IHMC-2
8.4. Mass Spectrum of IHMC-2
8.5. Spectral interpretation of IHMC-2

Figure S9. IHMC-3 Structure…………………………………………………………………… S17
9. IHMC-3 structure
9.1 TLC of IHMC-3
9.2. ^{1}H-NMR of IHMC-3
9.3. ^{13}C-NMR of IHMC-3
9.4. Mass Spectrum of IHMC-3
9.5. Spectral interpretation of IHMC-3

Figure S10. IHMC-4 Structure…………………………………………………………………… S19
10. IHMC-4 structure
10.1 TLC of IHMC-4
10.2. ^{1}H-NMR of IHMC-4
10.3. ^{13}C-NMR of IHMC-4
10.4. Mass Spectrum of IHMC-4
10.5. Spectral interpretation of IHMC-4

Figure S11. IHMC-5 Structure…………………………………………………………………… S21
11. IHMC-5 structure
11.1 TLC of IHMC-5
11.2. ^{1}H-NMR of IHMC-5
11.3. ^{13}C-NMR of IHMC-5
11.4. Mass Spectrum of IHMC-5
11.5. Spectral interpretation of IHMC-5

Figure S12. IHMC-6 Structure…………………………………………………………………… S23
12. IHMC-6 structure
12.1 TLC of IHMC-6
12.2. ^{1}H-NMR of IHMC-6
12.3. ^{13}C-NMR of IHMC-6
12.4. Mass Spectrum of IHMC-6
12.5. Spectral interpretation of IHMC-6

Figure S13. IHNC-1 Structure…………………………………………………………………… S25
13. IHNC-1 structure
13.1 TLC of IHNC-1
13.2. ^{1}H-NMR of IHNC-1
13.3. ^{13}C-NMR of IHNC-1
13.4. Mass Spectrum of IHNC-1
13.5. Spectral interpretation of IHNC-1

Figure S14. IHNC-2 Structure…………………………………………………………………… S27
14. IHNC-2 structure
14.1 TLC of IHNC-2
14.2. ^{1}H-NMR of IHNC-2
14.3. ^{13}C-NMR of IHNC-2
14.4. Mass Spectrum of IHNC-2
14.5. Spectral interpretation of IHNC-2

Figure S15. IHNC-3 Structure…………………………………………………………………… S29

15. IHNC-3 structure
15.1 TLC of IHNC-3
15.2. ^{1}H-NMR of IHNC-3
15.3. ^{13}C-NMR of IHNC-3
15.4. Mass Spectrum of IHNC-3
15.5. Spectral interpretation of IHNC-3

Figure S16. IHNC-4 Structure……………………………………………………….… S31
16. IHNC-4 structure
16.1 TLC of IHNC-4
16.2. ^{1}H-NMR of IHNC-4
16.3. ^{13}C-NMR of IHNC-4
16.4. Mass Spectrum of IHNC-4
16.5. Spectral interpretation of IHNC-4

Figure S17. IHNC-5 Structure……………………………………………………….… S33
17. IHNC-5 structure
17.1 TLC of IHNC-5
17.2. ^{1}H-NMR of IHNC-5
17.3. ^{13}C-NMR of IHNC-5
17.4. Mass Spectrum of IHNC-5
17.5. Spectral interpretation of IHNC-5

Figure S18. IHNC-6 Structure……………………………………………………….… S35
18. IHNC-6 structure
18.1 TLC of IHNC-6
18.2. ^{1}H-NMR of IHNC-6
18.3. ^{13}C-NMR of IHNC-6
18.4. Mass Spectrum of IHNC-6
18.5. Spectral interpretation of IHNC-6

Figure S19. MAO-B kinetics ……………………………………………………….… S37

Figure S1. IHC-1 Structure

1. IHC-1

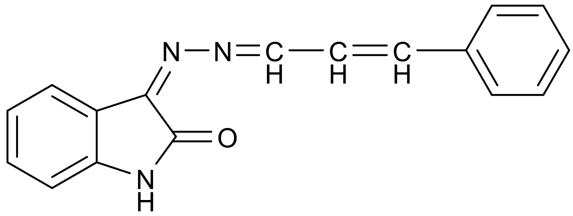

1.1 TLC OF IHC-1

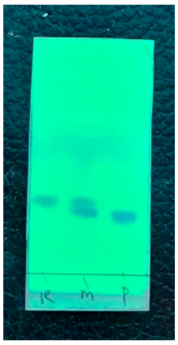

R = Reactant, M= Mixture, P = Product

1.2.^{1}H NMR SPECTRUM OF IHC-1

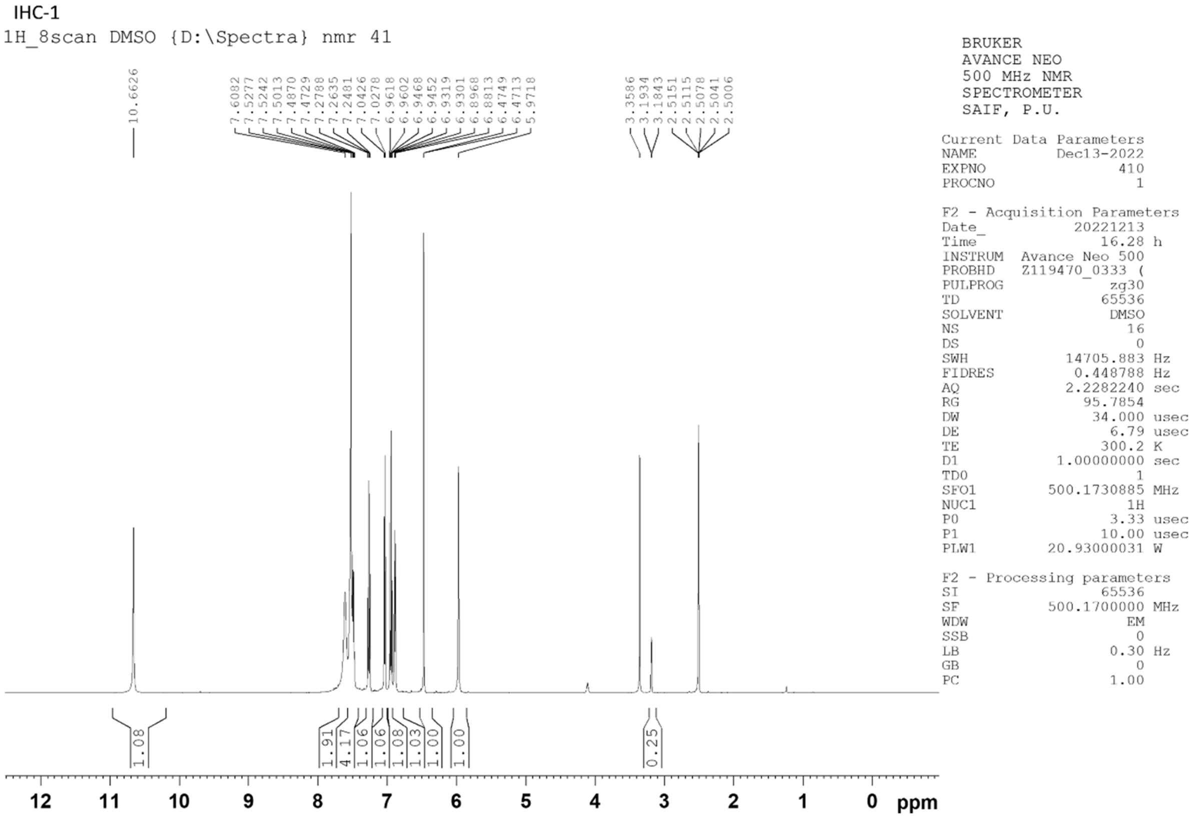

1.3. ^{13}C NMR OF IHC-1

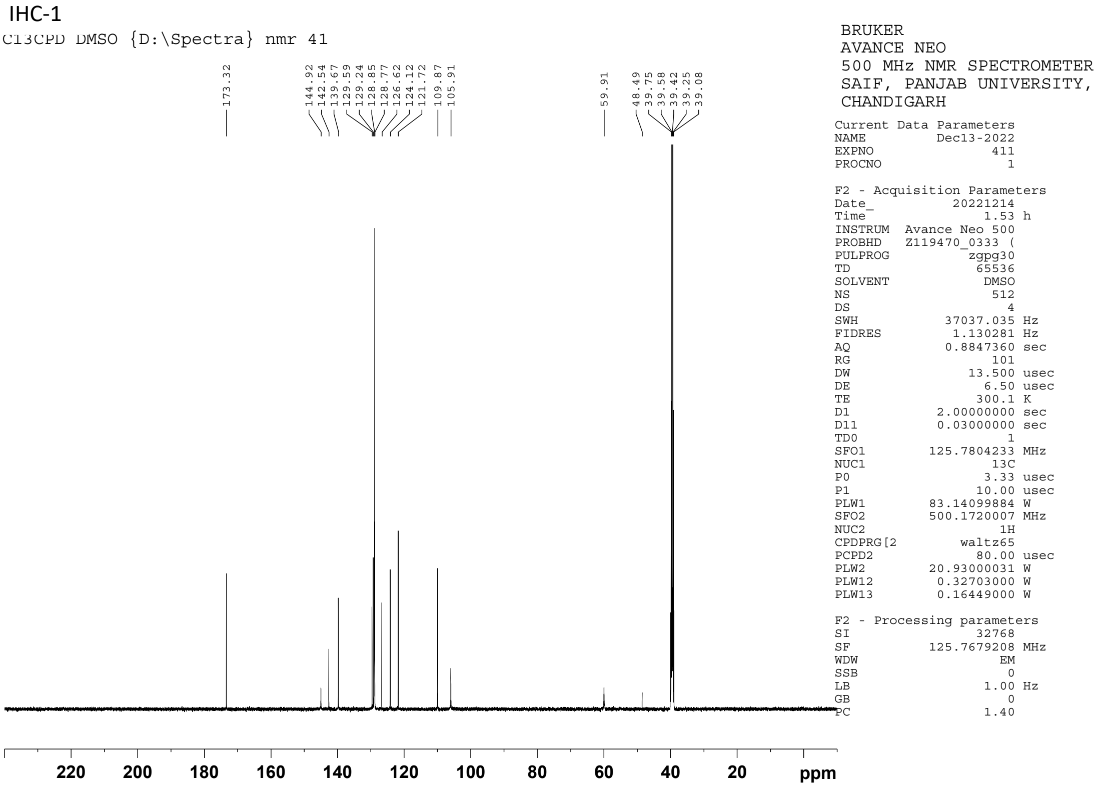

1.4. MASS SPECTRUM OF IHC-1

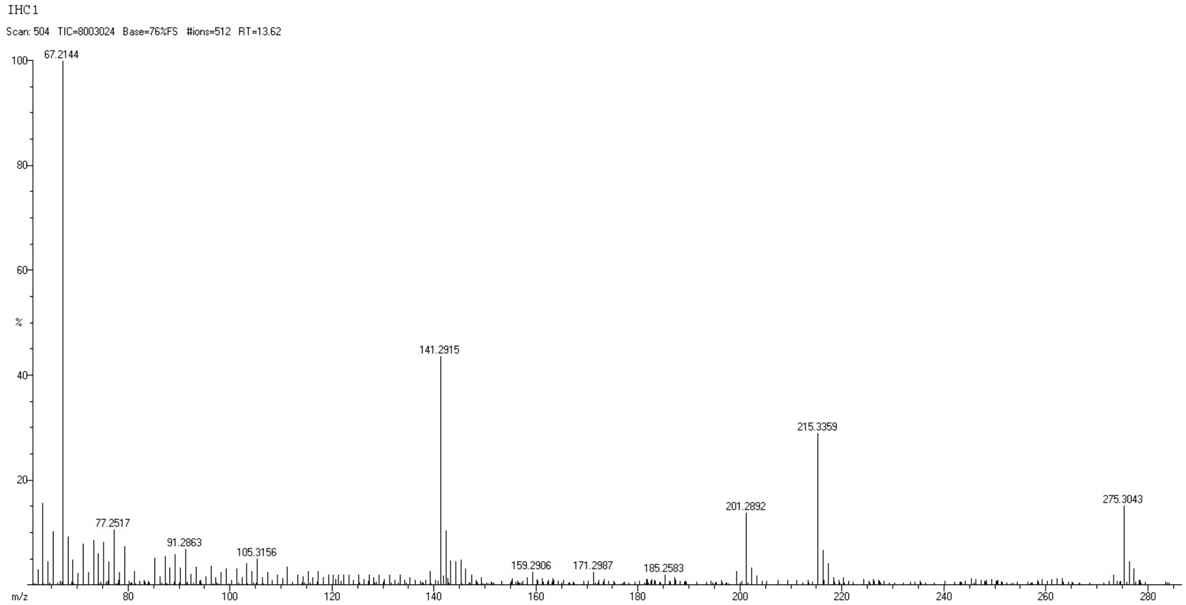

1.5. SPECTRAL INTERPRETATION

(3Z)-3-((3-phenylallylidene)hydrazineylidene)indolin-2-one(IHC-1): ^{1}H NMR (500 MHz, DMSO) δ 10.66 (s, 1H, NH), 7.61-6.95(m, 9H, Ar-H), 6.89(d, 1H, =CH-), 6.47(d, 1H -CH-) , 5.97(s, 1H, -CH-).^{13}C NMR (500 MHz, DMSO) δ: 173.32, 144.72, 142.54, 139.67, 129.59, 129.24, 128.85, 128.77, 126.62, 124.14, 121.72, 109.87, 105.91, 54.91, 48.49, 39.75, 39.08 . Molecular formula: $C_{17}H_{13}N_3O$, (HRMS)Calculated Mol.wt. = 275.30462, observed Mol.wt.= 275.3043.

Figure S2. IHC-2 Structure

2.IHC-2

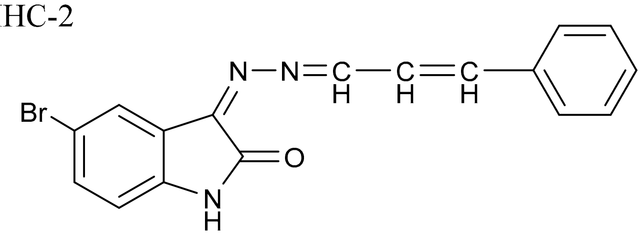

2.1. TLC OF IHC-2

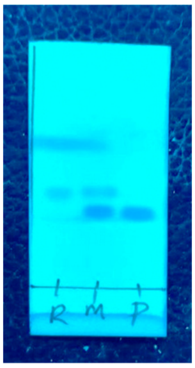

R = Reactant, M= Mixture, P = Product

2.2. ^{1}H NMR OF IHC-2

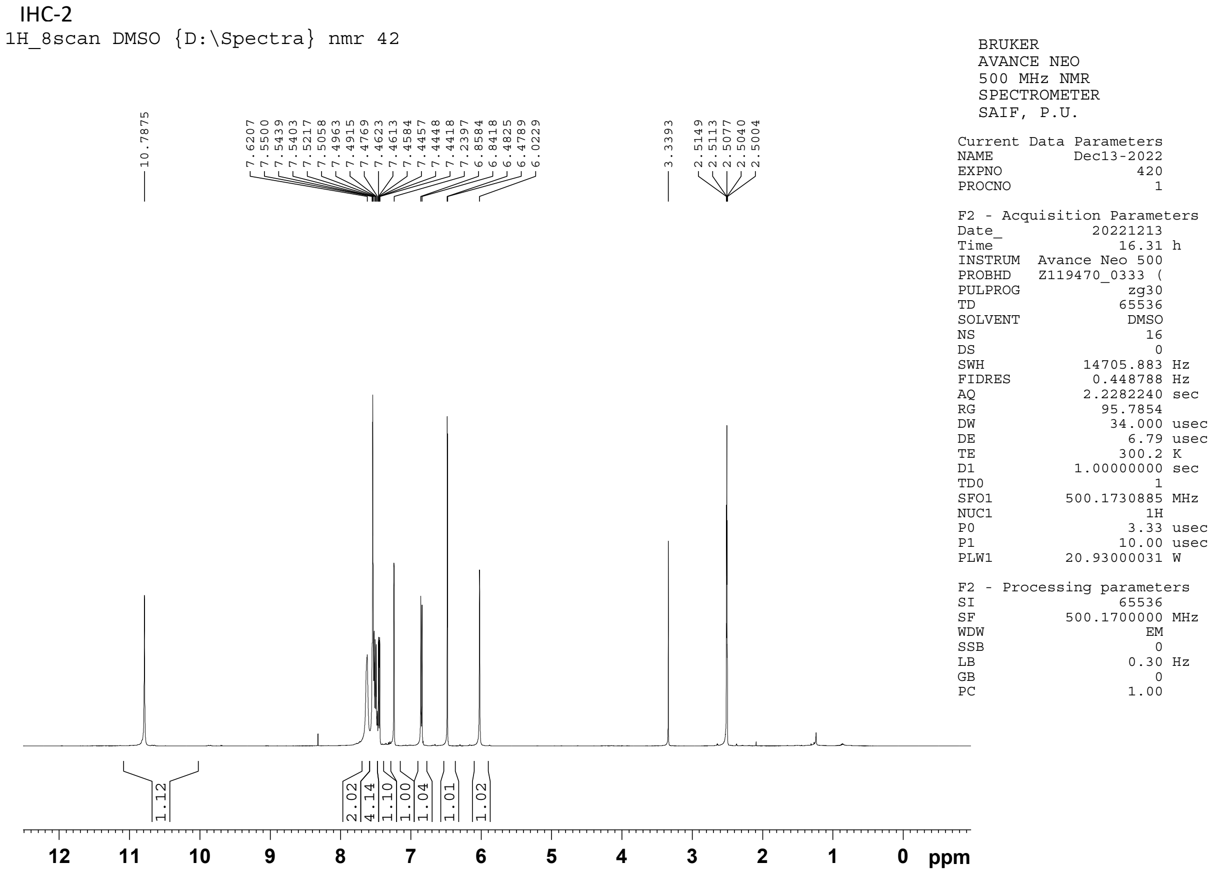

2.3 ^{13}C NMR OF IHC-2

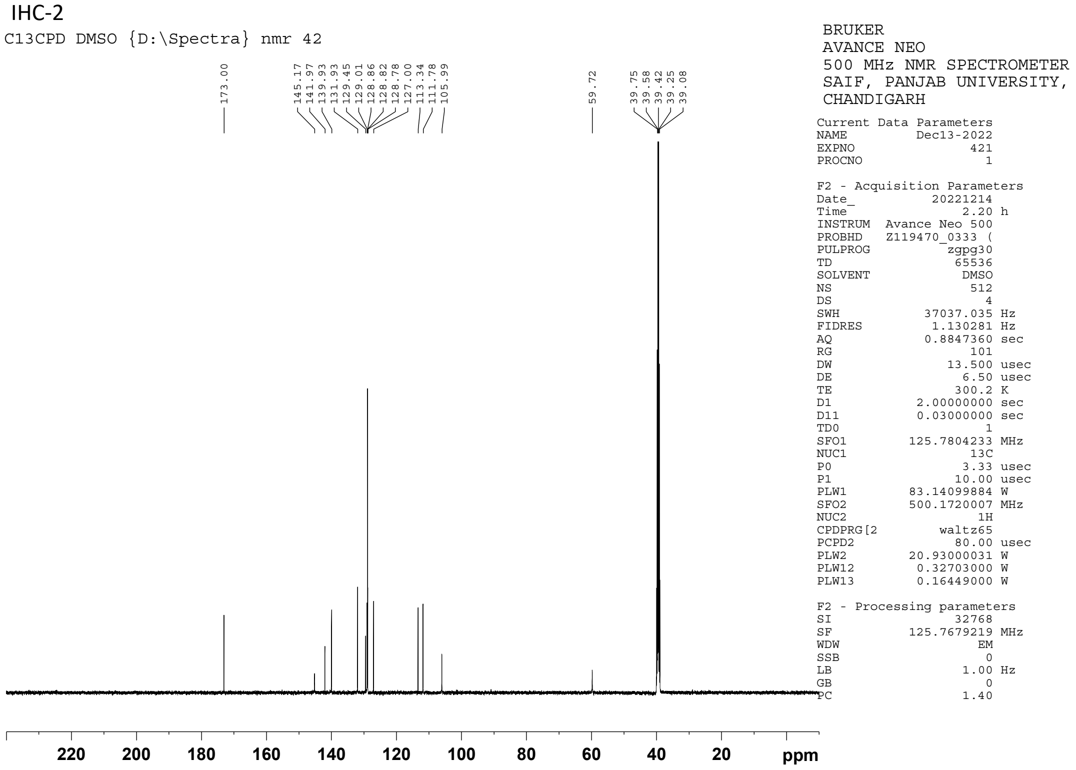

2.4.MASS SPECTRUM OF IHC-2

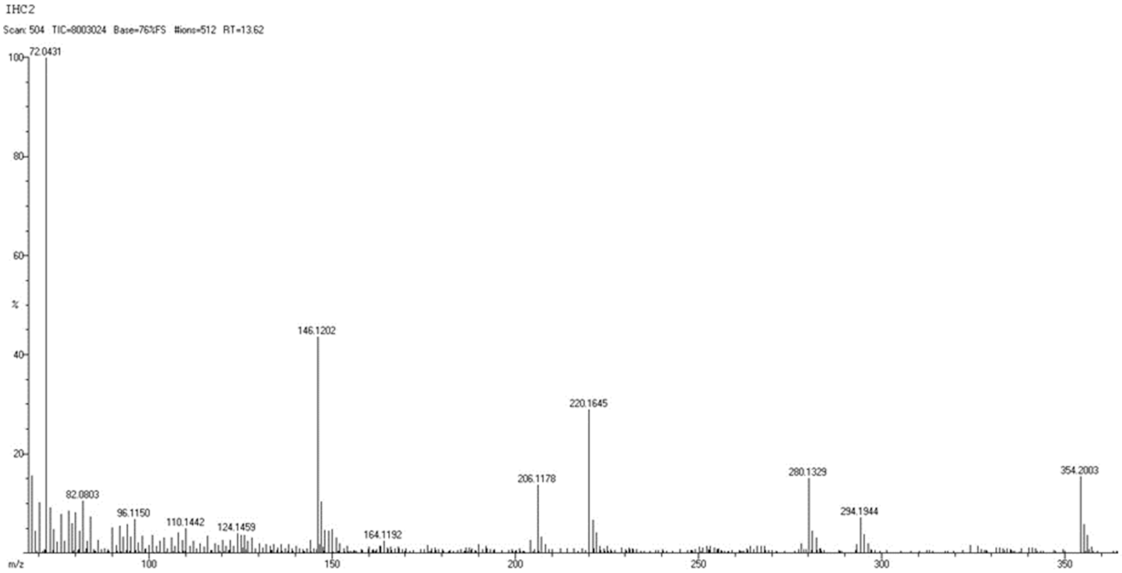

2.5. SPECTRAL INTERPRETATION

(3Z)-5-bromo-3-((3-phenylallylidene)hydrazineylidene)indolin-2-one(IHC-2): ^{1}H NMR (500 MHz, DMSO) δ 10.79 (s, 1H,NH), 7.62-7.24 (m, 8H,Ar-H),6.84 (d,1H,=CH-), 6.48 (d,1H,-CH-), 6.02 (s, 1H,-CH-).13C NMR(500 MHz, DMSO)δ: 173, 145.17, 1471.97, 139.93, 131.93, 129.45, 129.01, 128.86, 128.82, 128.78, 127, 113.34, 111.78, 105.99, 59.72.Molecular Formula: $C_{17}H_{12}BrN_3O$,(HRMS)Calculated Mol.wt.= 354.21, Observed Mol.wt. = 354.2003.

Figure S3. IHC-3 Structure

3. IHC-3

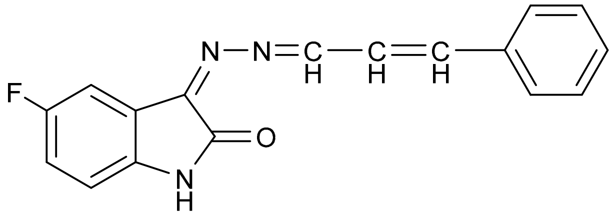

3.1 TLC OF IHC-3

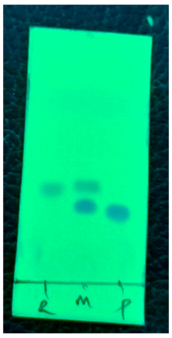

R = Reactant, M= Mixture, P = Product

3.2. ^{1}H NMR OF IHC-3

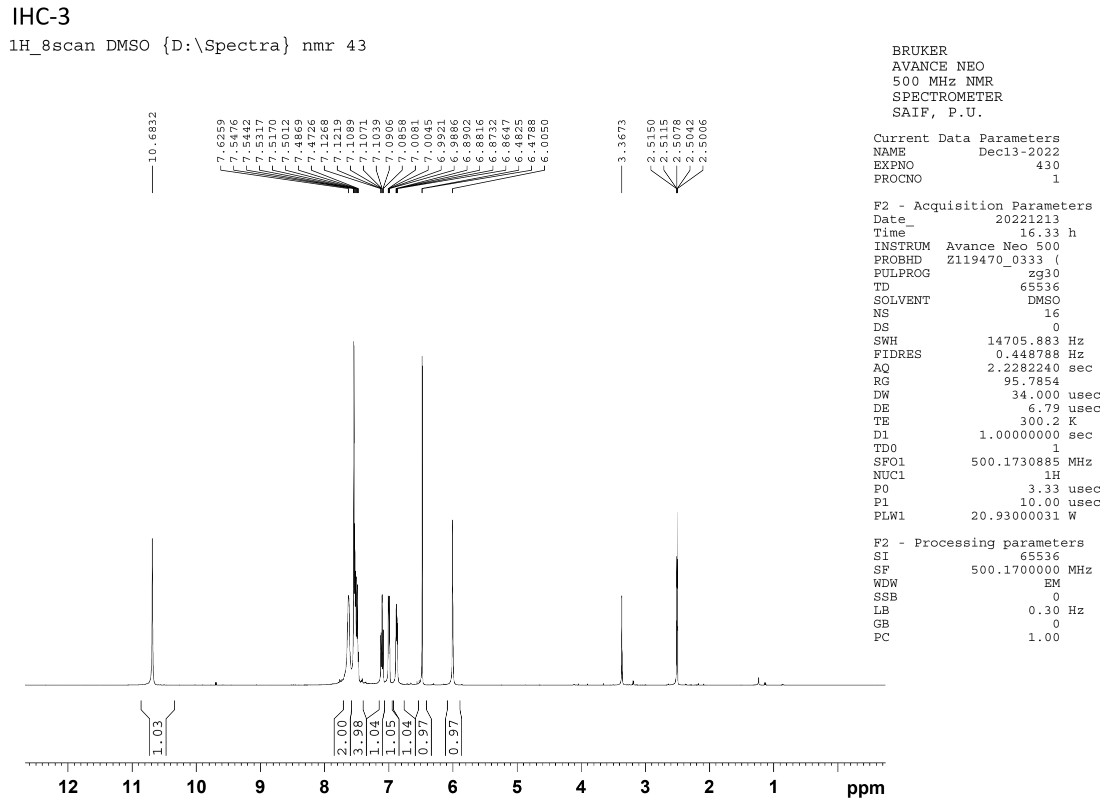

3.3.[13C] NMR OF IHC-3

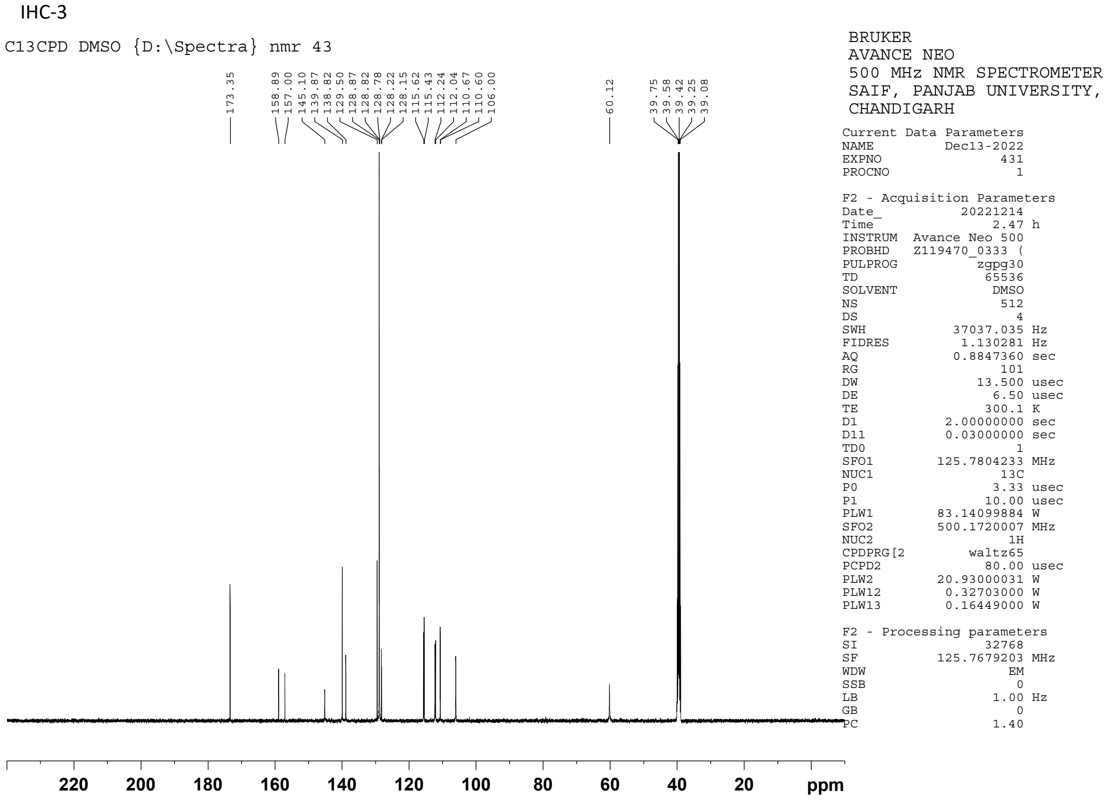

3.4.MASS SPECTRUM OF IHC-3

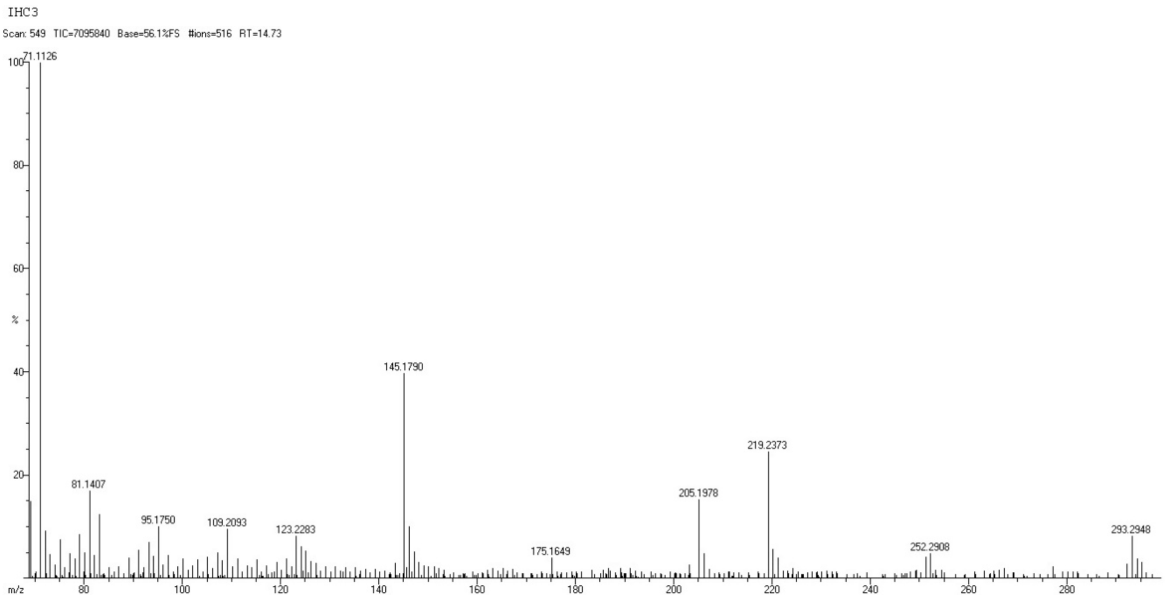

3.5. SPECTRAL INTERPRETATION

(3Z)-5-fluoro-3-((3-phenylallylidene)hydrazineylidene)indolin-2-one(IHC-3):^{1}H NMR (500 MHz, DMSO) δ 10.68 (s, 1H, NH), 7.63-6.87(m, 9H,Ar-H,=CH), 6.48 (d, 1H,-CH-), 6.00 (s, 1H, -CH-). 13C NMR (500 MHz, DMSO) δ:173.35, 158.89, 157, 145.10, 139.87, 138.82, 129.50, 128.87, 128.82, 128.78, 128.15, 115.62, 115.24, 112.24, 110.67, 106, 60.12. Molecular formula: $C_{17}H_{12}FN_3O$, (HRMS), Calculated Mol.wt. 293.30, Observed Mol.wt. 293.2948.

Figure S4. IHC-4 Structure

4. IHC-4

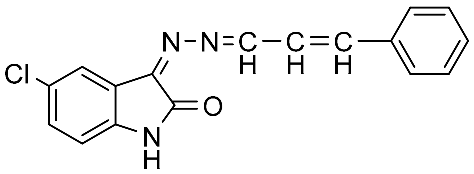

4.1 TLC OF IHC-4

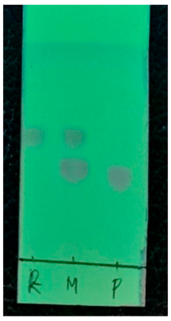

R = Reactant, M= Mixture, P = Product

4.2.^{1}H NMR OF IHC-4

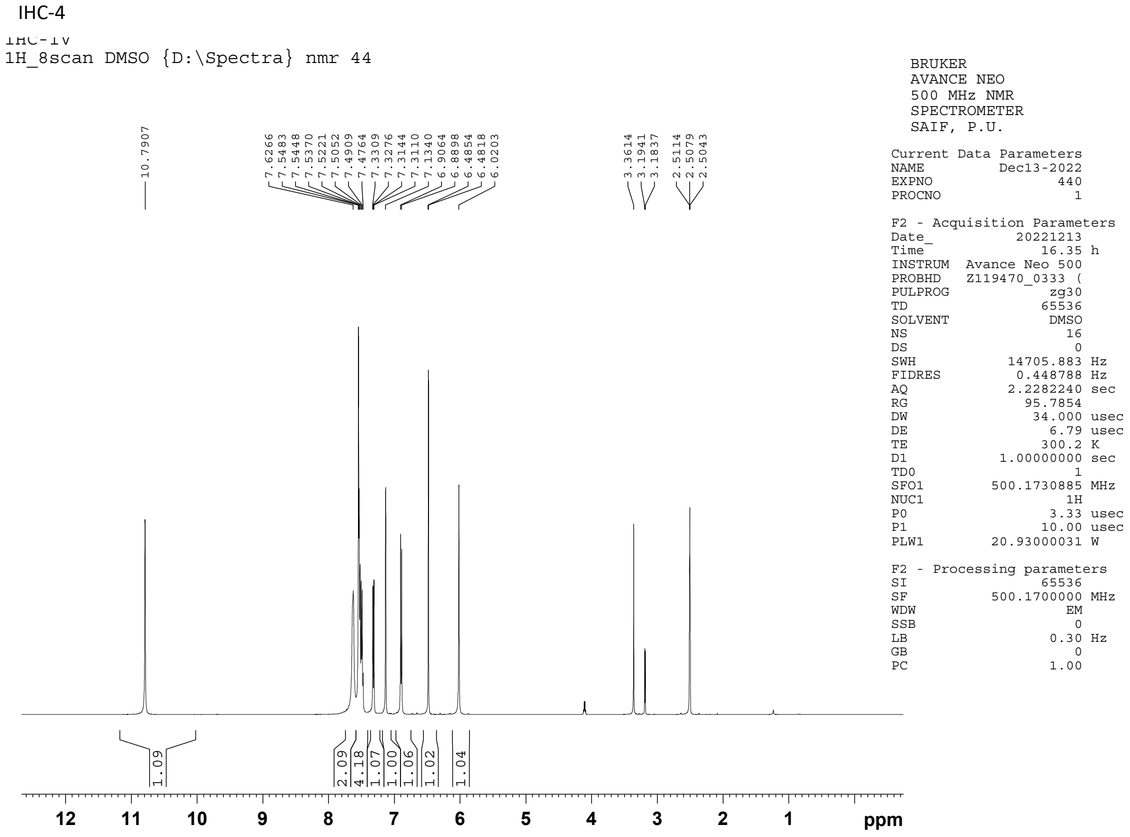

4.3. ^{13}C NMR OF IHC-4

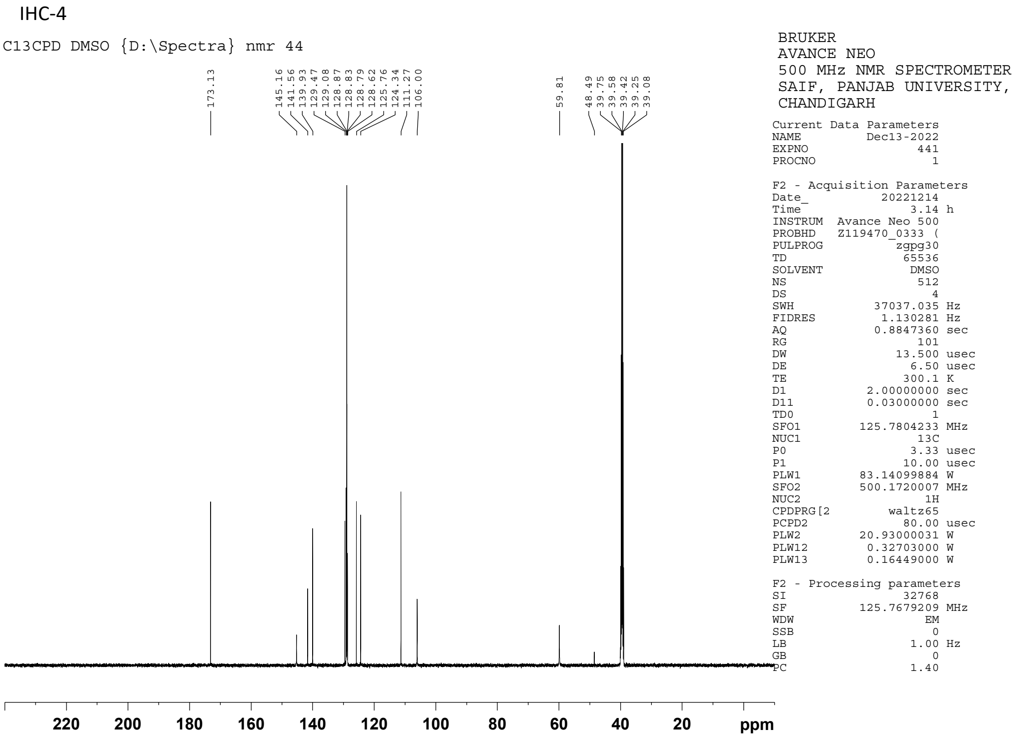

4.4.MASS SPECTRUM OF IHC-4

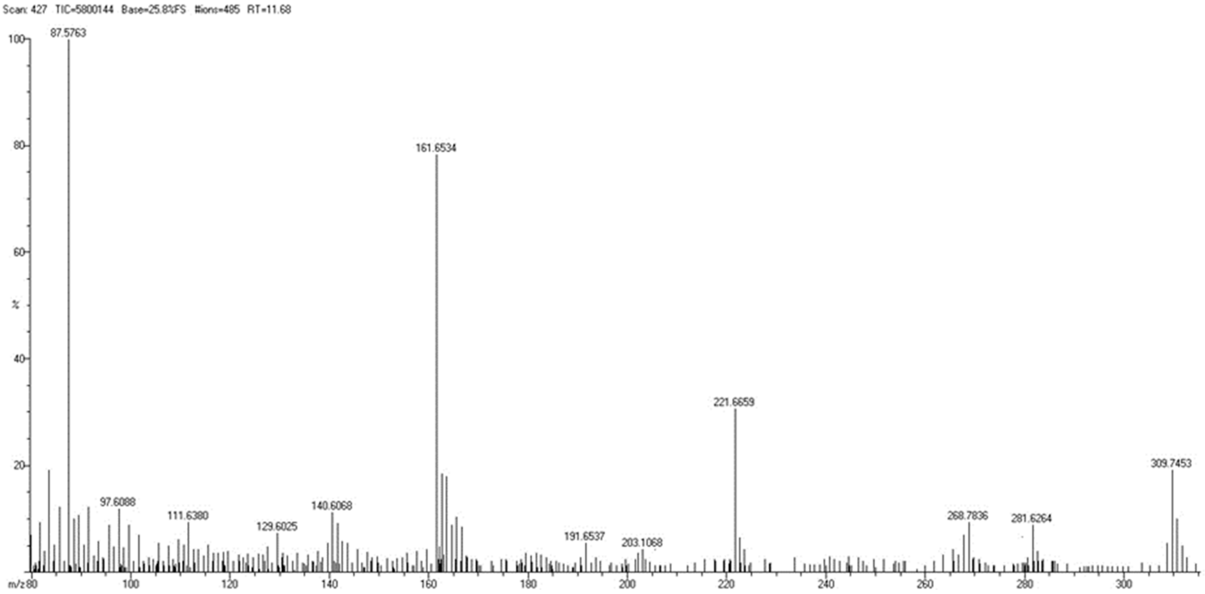

4.5. SPECTRUM INTERPRETATION

(3Z)-5-chloro-3-((3-phenylallylidene)hydrazineylidene)indolin-2-one(IHC-4): ^{1}H NMR (500 MHz, DMSO) δ 10.79 (s,1H,NH), 7.63-7.13 (m, 8H, Ar-H), 6.90 (d,1H=CH-), 6.48 (d,1H,-CH-), 6.02 (s,1H,-CH-). 13C NMR (500 MHz, DMSO) δ: 173.13, 145.16, 141.56, 139.93, 129.47, 129.08, 128.87, 128.83, 128.79, 128.62, 125.76, 124.34, 111.27, 106, 59.81, 48.49. Molecular formula: $C_{17}H_{12}ClN_3O$,(HRMS)Calculated Mol.wt. = 309.75, Observed Mol.wt. = 309.7453.

Figure S5. IHC-5 Structure

5. IHC-5

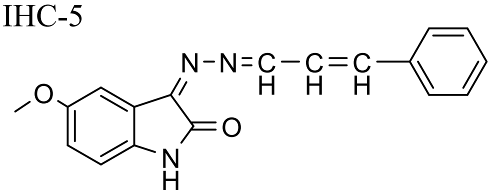

5.1 TLC OF IHC-5

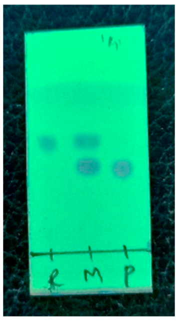

R = Reactant, M= Mixture, P = Product

5.2.^{1}H NMR OF IHC-5

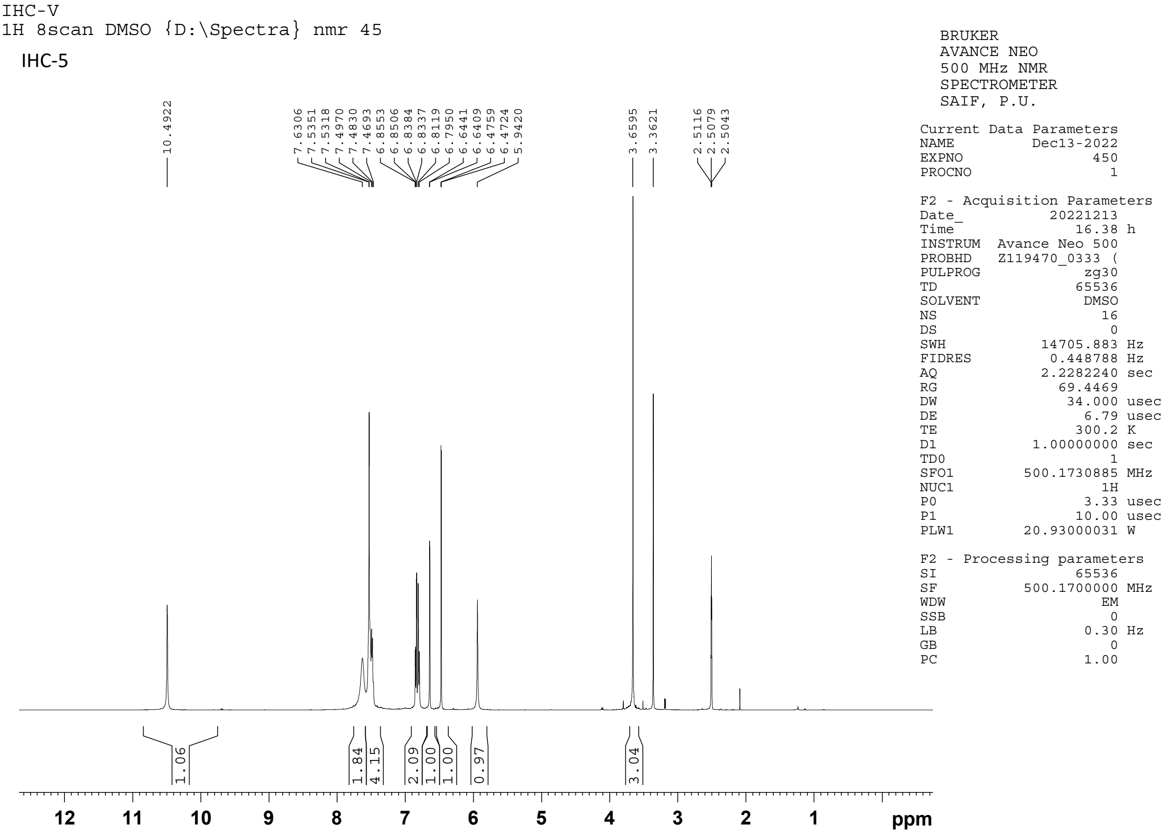

5.3. ^{13}C NMR OF IHC-5

IHC-5

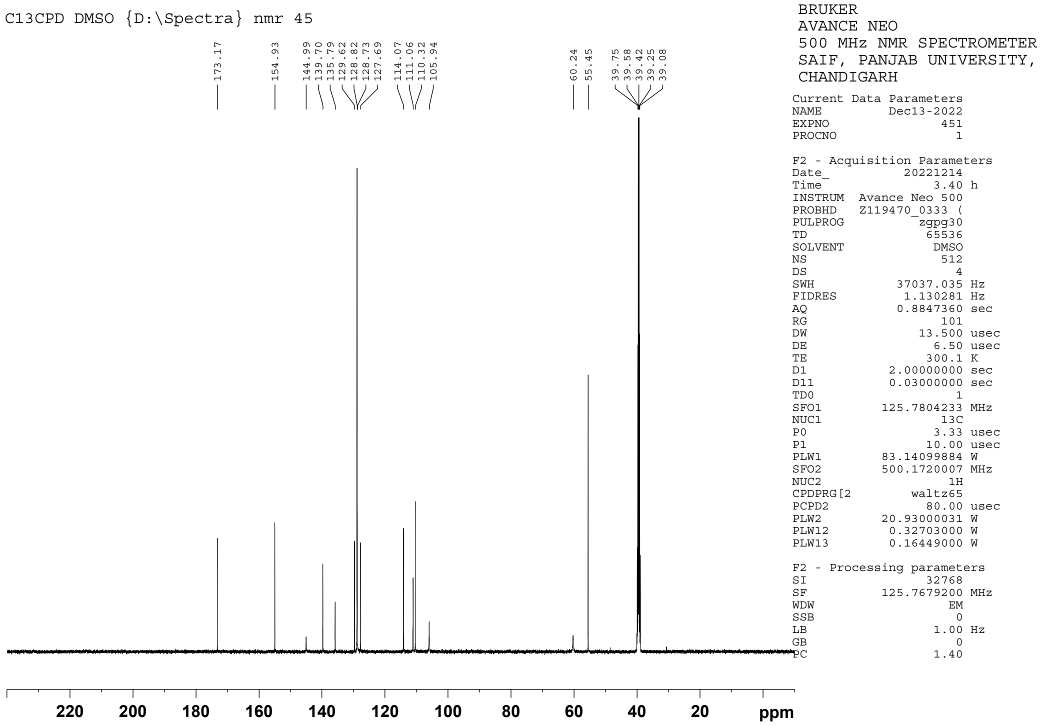

5.4.MASS SPECTRUM OF IHC-5

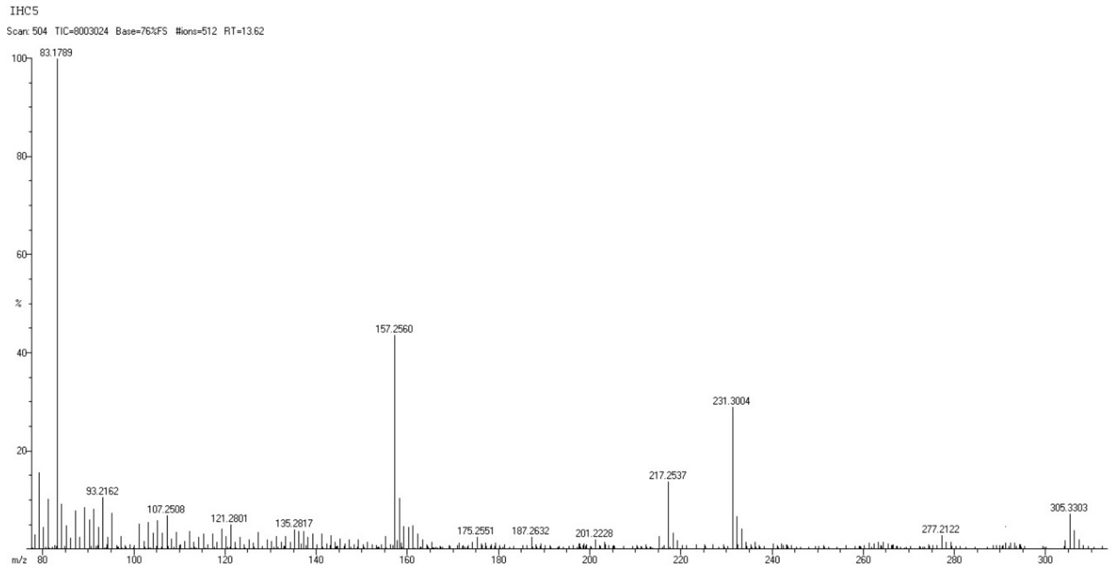

5.5. SPECTRUM INTERPRETATION

(3Z)-5-methoxy-3-((3-phenylallylidene)hydrazineylidene)indolin-2-one(IHC-5) ^{1}H NMR (500 MHz, DMSO) δ: 10.49 (s,1H,NH), 7.69-6.64(m,9H, Ar-H,=CH-) 6.47 (d,1H -CH-), 5.94 (s,1H,-CH-), 3.66 (s,3H,-OCH_3-).^{13}C NMR (500 MHz, DMSO) δ: 173.17, 154.93, 144.99, 139.70, 135.79, 129.62, 128.82, 128.73, 127.69, 114.07, 111.06, 110.32, 105.94.Molecular formula:$C_{18}H_{15}N_3O_2$, (HRMS)Calculated Mol.wt. = 305.34, Observed Mol.wt. = 305.3303

Figure S6. IHC-6 Structure

6. IHC-6

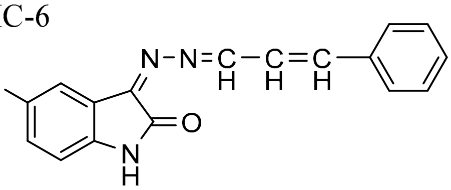

6.1 TLC OF IHC-6

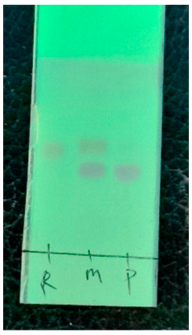

R = Reactant, M= Co-mixture, P = Product

6.2.^{1}H NMR OF IHC-6

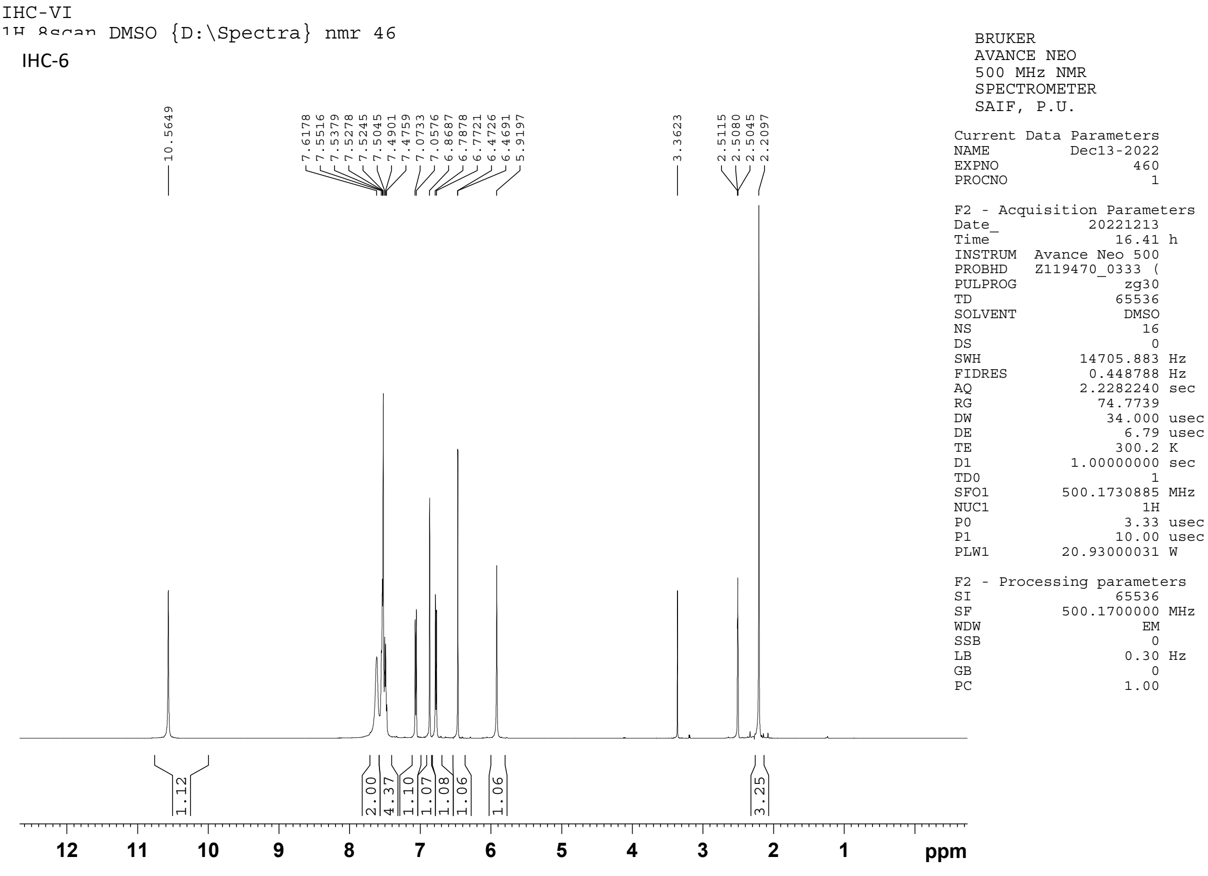

6.3.^{13}C NMR OF IHC-6

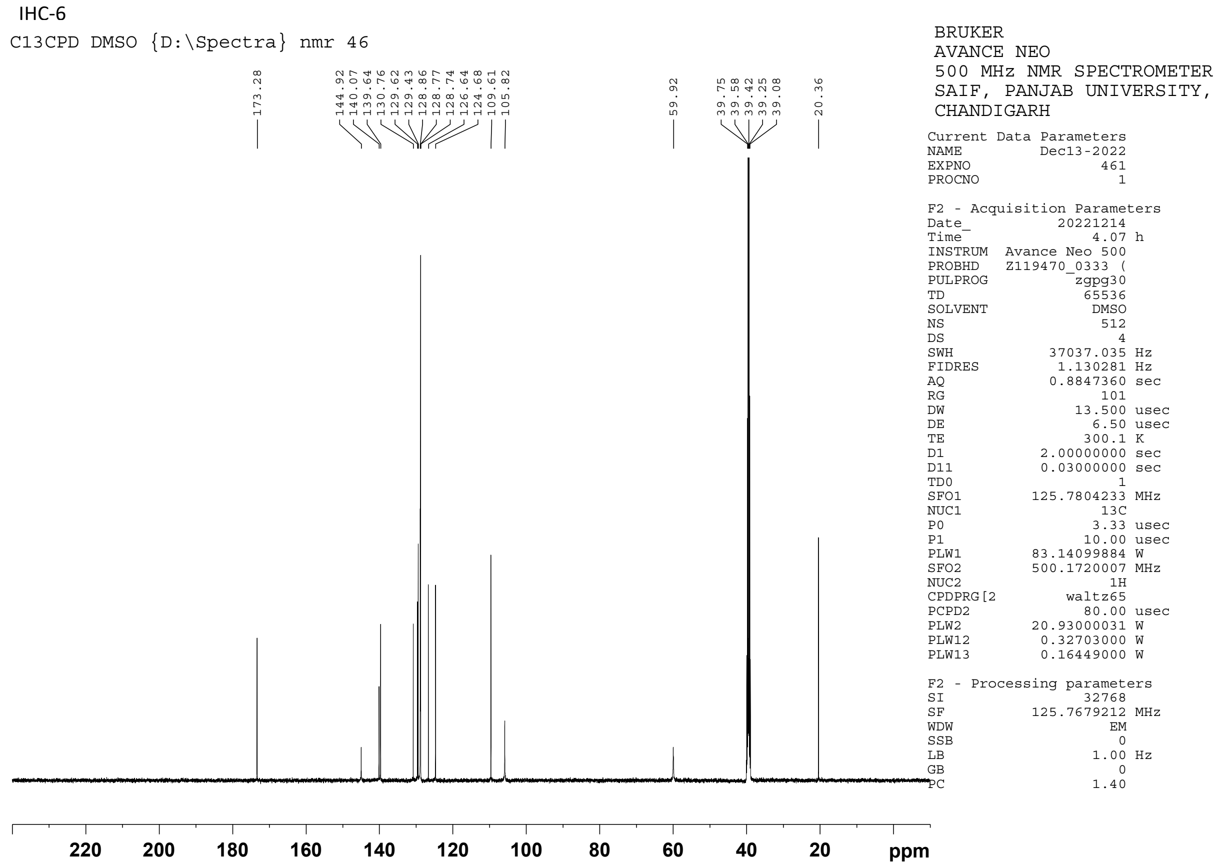

6.4.MASS SPECTRUM OF IHC-6

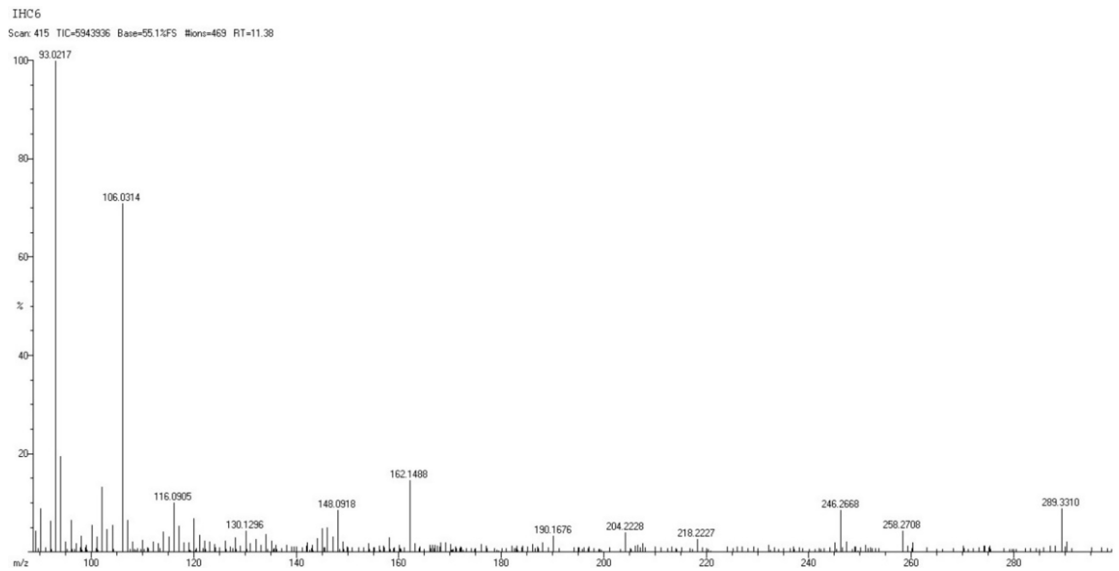

6.5. SPECTRUM INTERPRETATION

(3Z)-5-methyl-3-((3-phenylallylidene)hydrazineylidene)indolin-2-one(IHC-6) ^{1}H NMR (500 MHz, DMSO) δ: 10.56 (s, 1H, NH), 7.62-6.87 (m, 8H,Ar-H), 6.78 (d,1H,=CH-), 6.47 (d, 1H, -CH-), 5.92 (s,1H-CH-), 2.21 (s,3H,-CH_3). ^{13}C NMR (500 MHz, DMSO) δ: 173.28, 144.92, 140.07, 139.64, 130.76, 129.62, 129.43, 128.77, 128.74, 126.64, 124.68, 109.61, 105.82, 59.92,20.36. Molecular formula: $C_{18}H_{15}N_3O$,(HRMS) Calculated Mol.wt. = 289.34, Observed Mol.wt. = 289.3310.

Figure S7. IHMC-1 Structure

7. IHMC-1

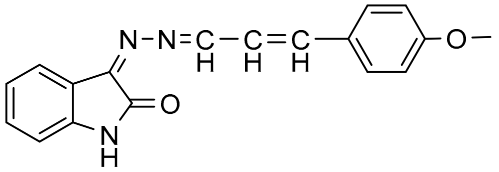

7.1 TLC OF IHMC-1

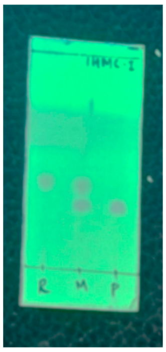

R = Reactant, M= Mixture, P = Product

7.2. ^{1}H NMR OF IHMC-1

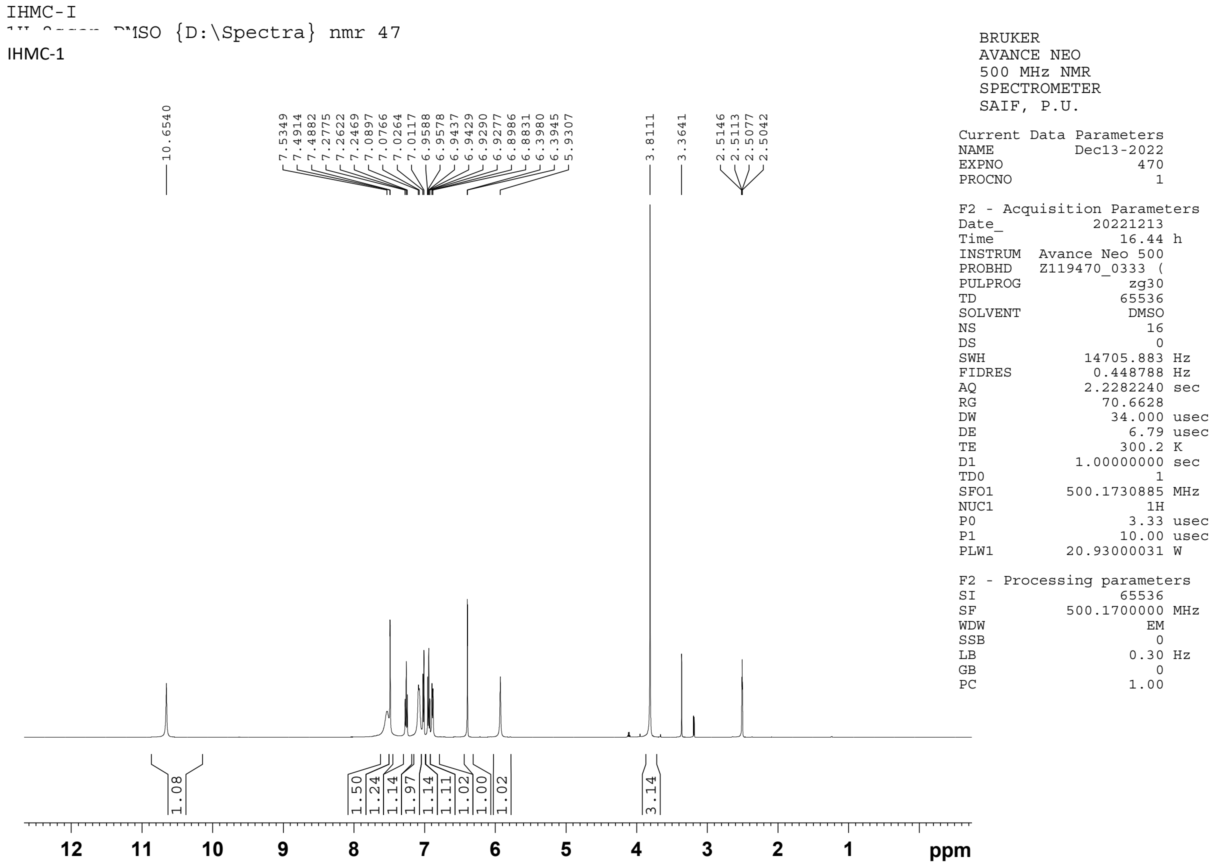

7.3.13C NMR OF IHMC-1

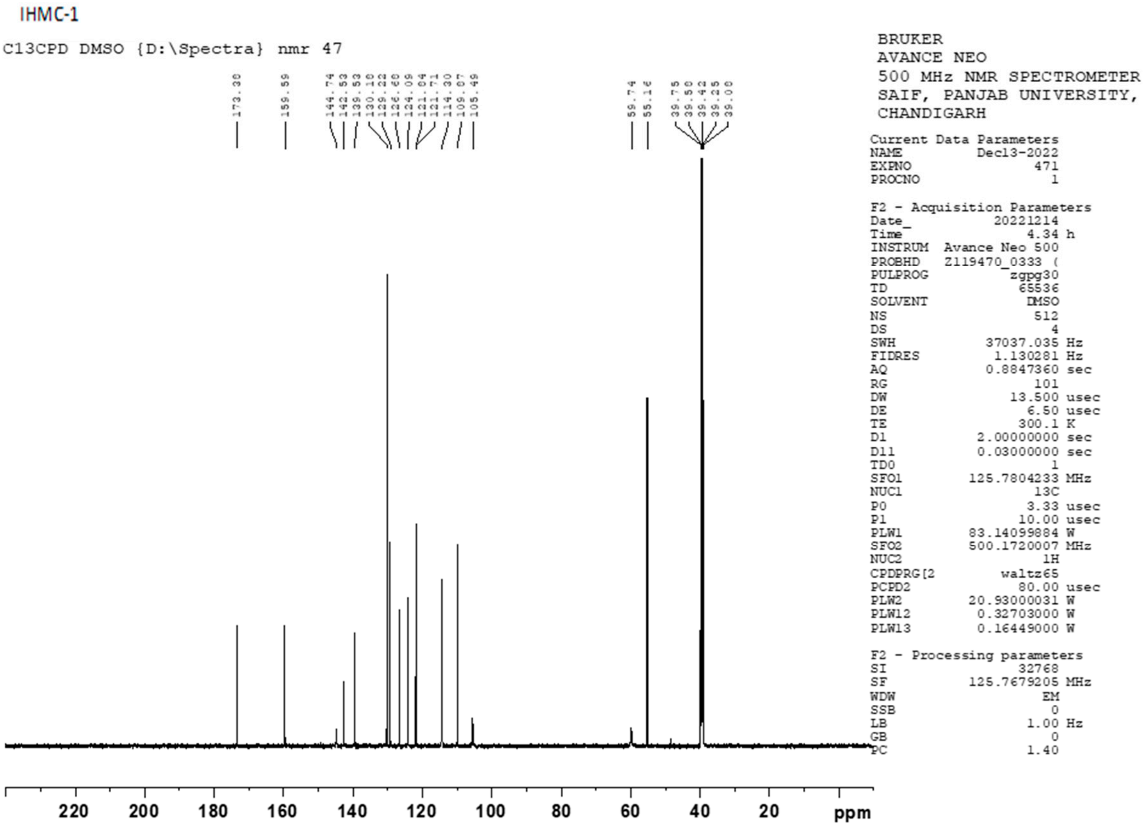

7.4.MASS SPECTRUM OF IHMC-1

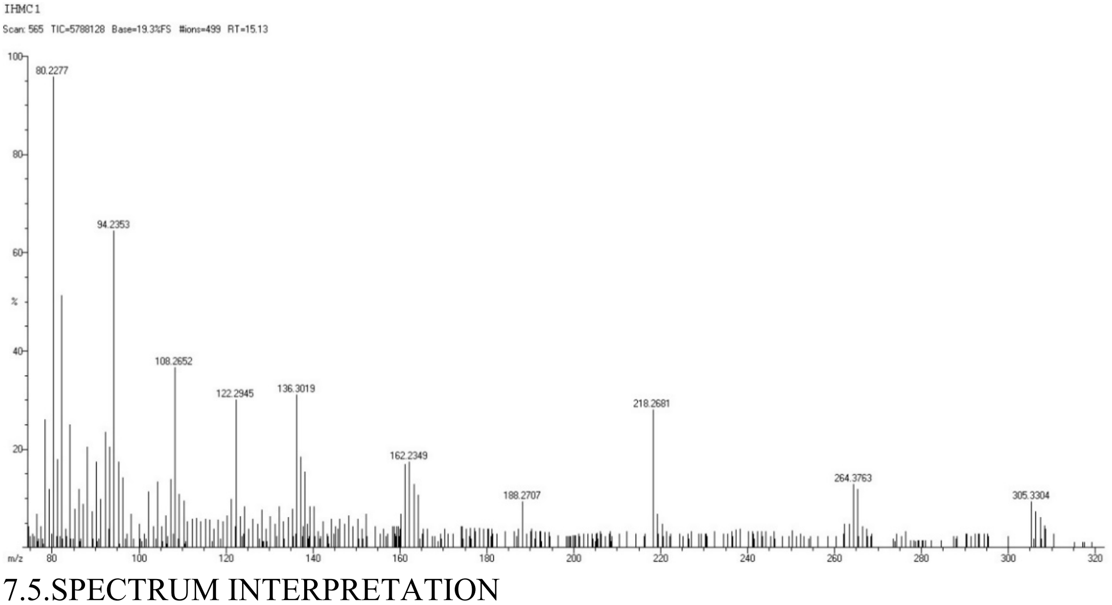

7.5.SPECTRUM INTERPRETATION

(3Z)-3-((3-(4-methoxyphenyl)allylidene)hydrazineylidene)indolin-2-one(IHMC-1) 1H NMR (500 MHz, DMSO) δ 10.65 (s, 1H,NH), 7.53-6.94(m,8H,Ar-H), 6.89 (d,1H,=CH-), 6.40 (d,1H,-CH-), 5.93 (s, 1H,-CH-), 3.81 (s, 3H,-OCH3).13C NMR (500 MHz, DMSO) δ 173.38, 159.59, 144.74, 142.53, 139.53, 130.18, 129.22,126.68, 124.09, 121.84, 121.71, 114.30, 109.87, 105.49, 59.74, 55.16. Molecular formula: $C_{18}H_{15}N_3O_2$ (HRMS), Calculated Mol.wt. = 305.34, Observed Mol.wt. = 305.3304

Figure S8. IHMC-2 Structure

8. IHMC-2

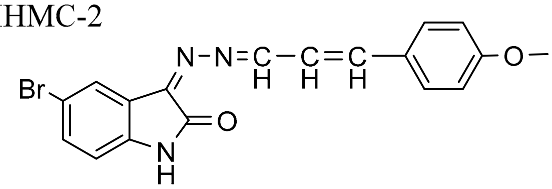

8.1 TLC OF IHMC-2

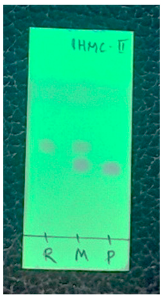

R = Reactant, M=Mixture, P = Product

8.2.^{1}H NMR OF IHMC-2

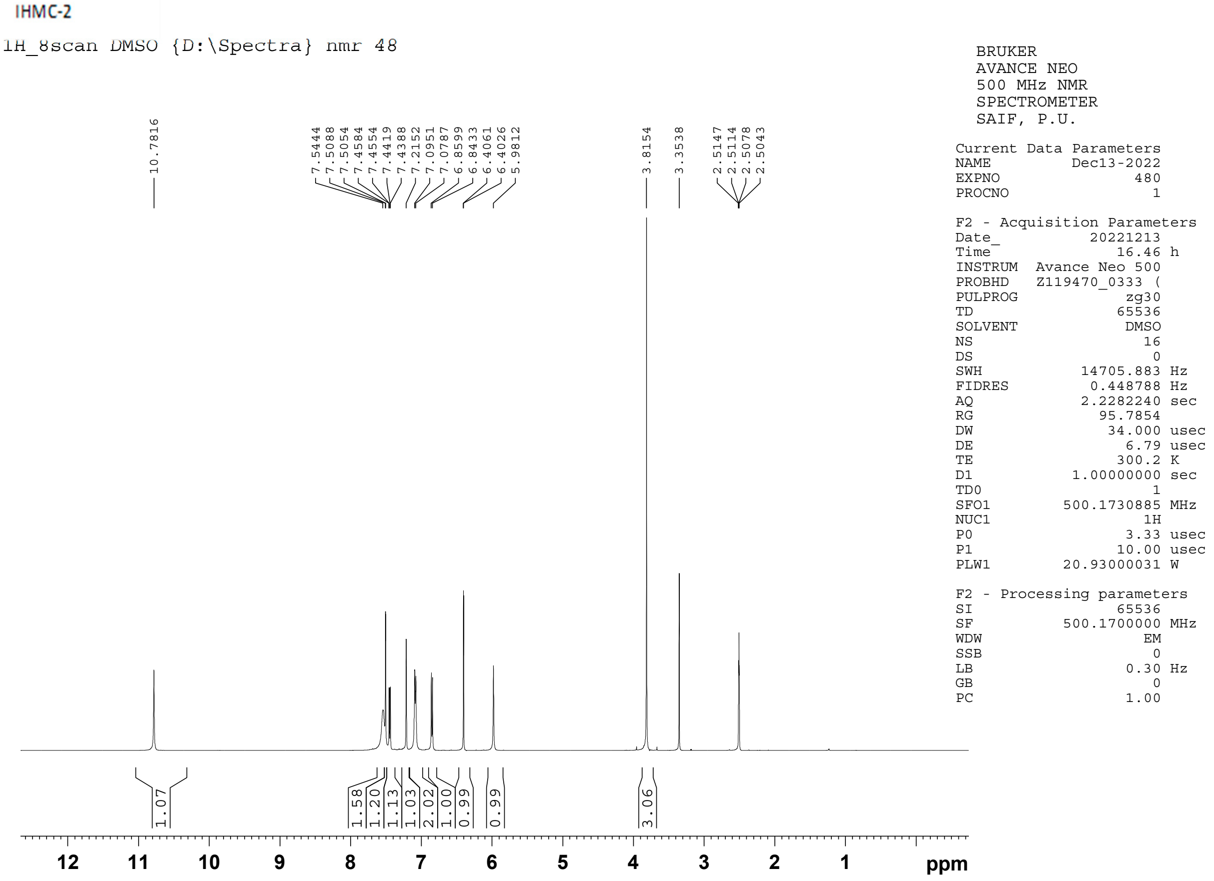

8.3.^{13}C NMR OF IHMC-2

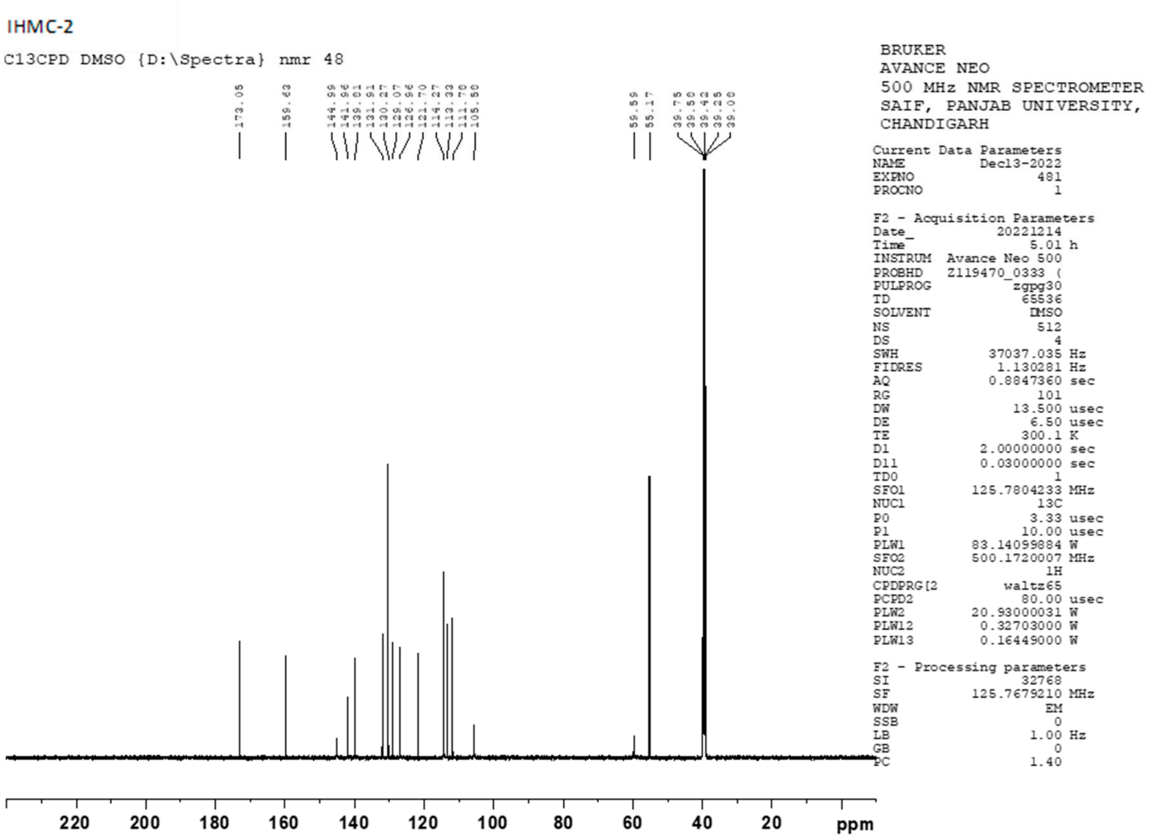

8.4.MASS SPECTRUM OF IHMC-2

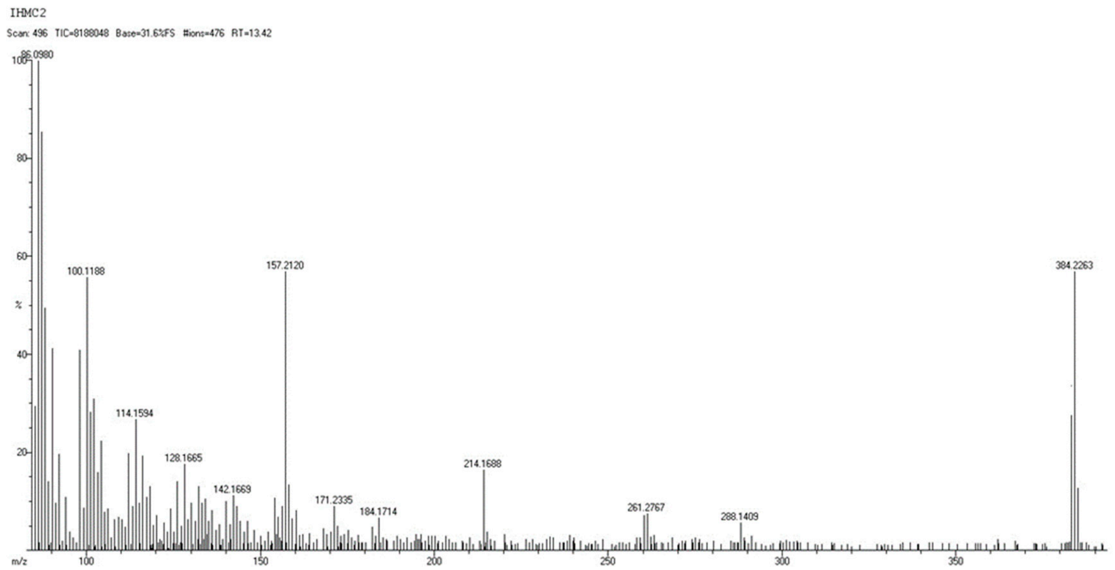

8.5.SPECTRUM INTERPRETATION

(3Z)-5-bromo-3-((3-(4-methoxyphenyl)allylidene)hydrazineylidene)indolin-2-one(IHMC-2)1H NMR (500 MHz, DMSO) δ 10.78 (s,1H,NH), 7.62-7.09(m, 7H,Ar-H), 6.84 (d,1H,=CH-), 6.40 (d,1H,-CH-), 5.98 (s,1H,-CH-), 3.82 (s, 3H,-OCH3).13C NMR (500 MHz, DMSO) δ 173.05, 159.63, 144.99, 141.96, 139.81, 131.91, 130.27, 129.07, 126.96, 121.70, 114.27, 113.33, 111.78, 105.58, 59.59, 55.17. Molecular formula: C18H14BrN3O2 (HRMS),Calculated Mol.wt.=384.23,Observed Mol.wt. = 384.2263.

Figure S9. IHMC-3 Structure

9. IHMC-3

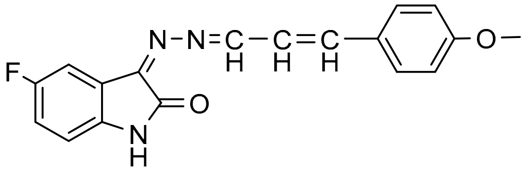

9.1 TLC OF IHMC-3

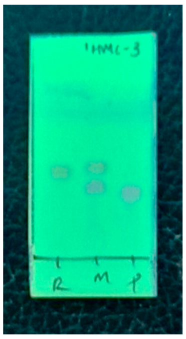

R = Reactant, M= Mixture, P = Product

9.2.^{1}H NMR OF IHMC-3

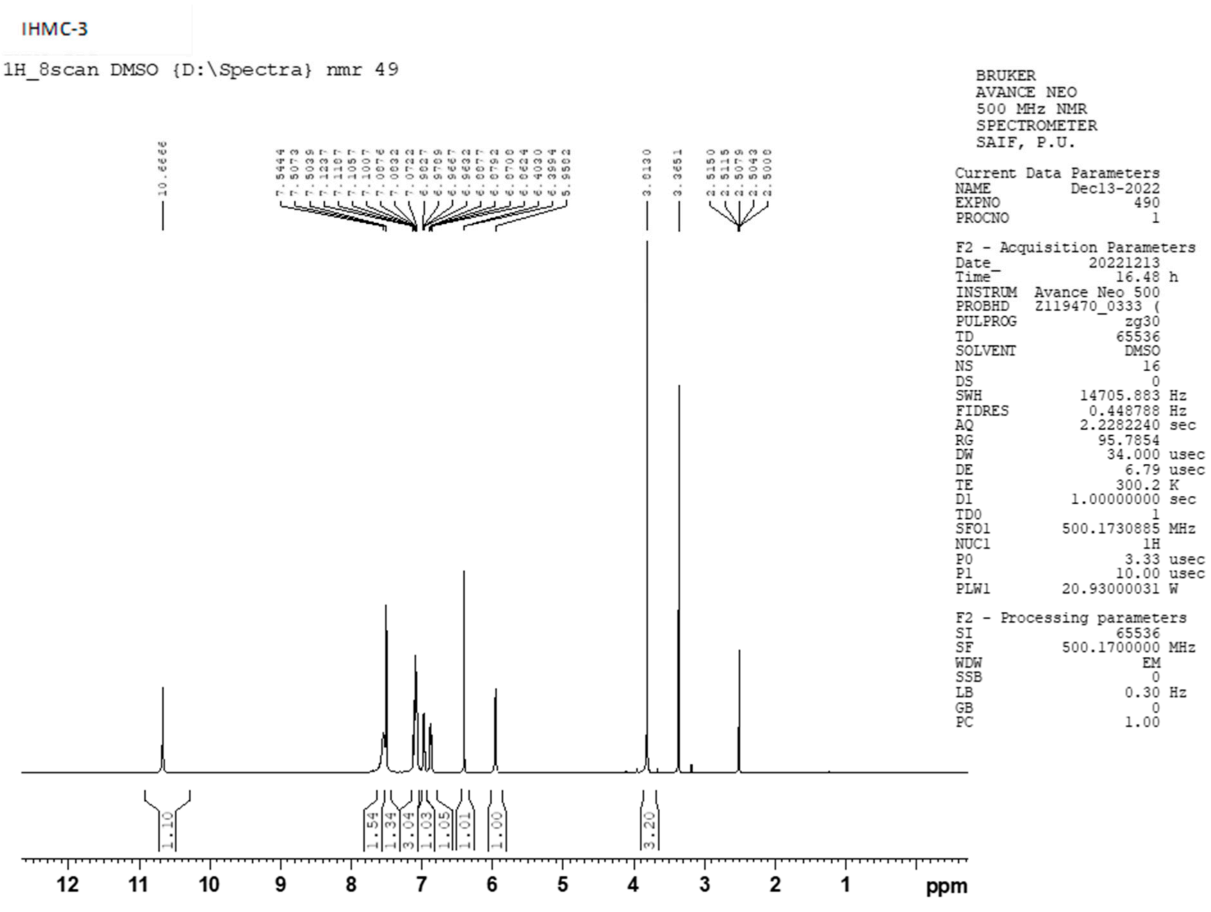

9.3. ^{13}C NMR OF IHMC-3

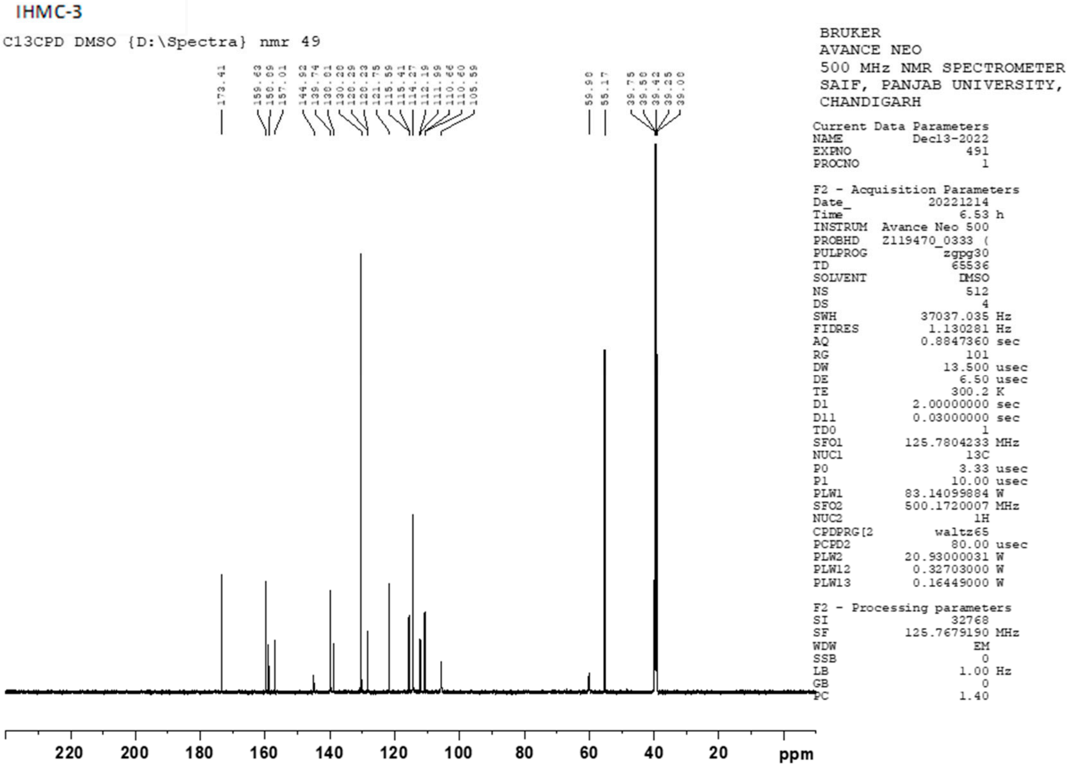

9.4. MASS SPECTRUM OF IHMC-3

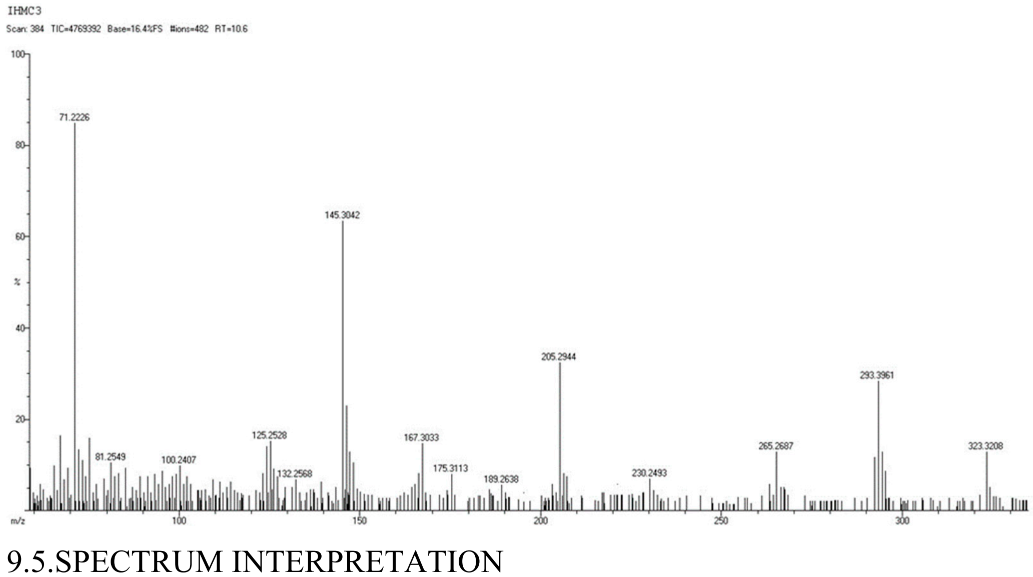

9.5. SPECTRUM INTERPRETATION

(3Z)-5-fluoro-3-((3-(4-methoxyphenyl)allylidene)hydrazineylidene)indolin-2-one(IHMC-3) 1H NMR (500 MHz, DMSO) δ 10.67 (s, 1H,NH), 7.54-6.88(m,8H, Ar-H, =CH-),6.40 (d,1H,-CH-), 5.96 (s,1H,-CH-),3.81 (s,3H,-OCH3). 13C NMR (500 MHz, DMSO) δ 173.41, 159.63,158.89,157.01,144.92,193.74,138.81,130.28,128.29,121.75,115.59,114.27,112.19,111.99,110.66,105.59,59.98,55.17.Molecular formula:C18H14FN3O2(HRMS) Calculated Mol.wt.=323.33, Observed Mol.wt.=323.3208.

Figure S10. IHMC-4 Structure

10. IHMC-4

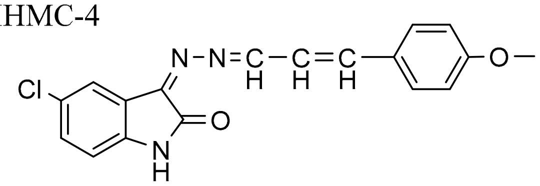

10.1 TLC OF IHMC-4

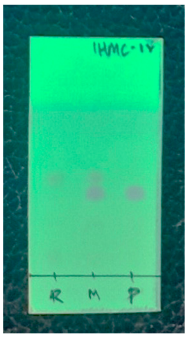

R = Reactant, M= Mixture, P = Product

10.2.^{1}H NMR OF IHMC-4

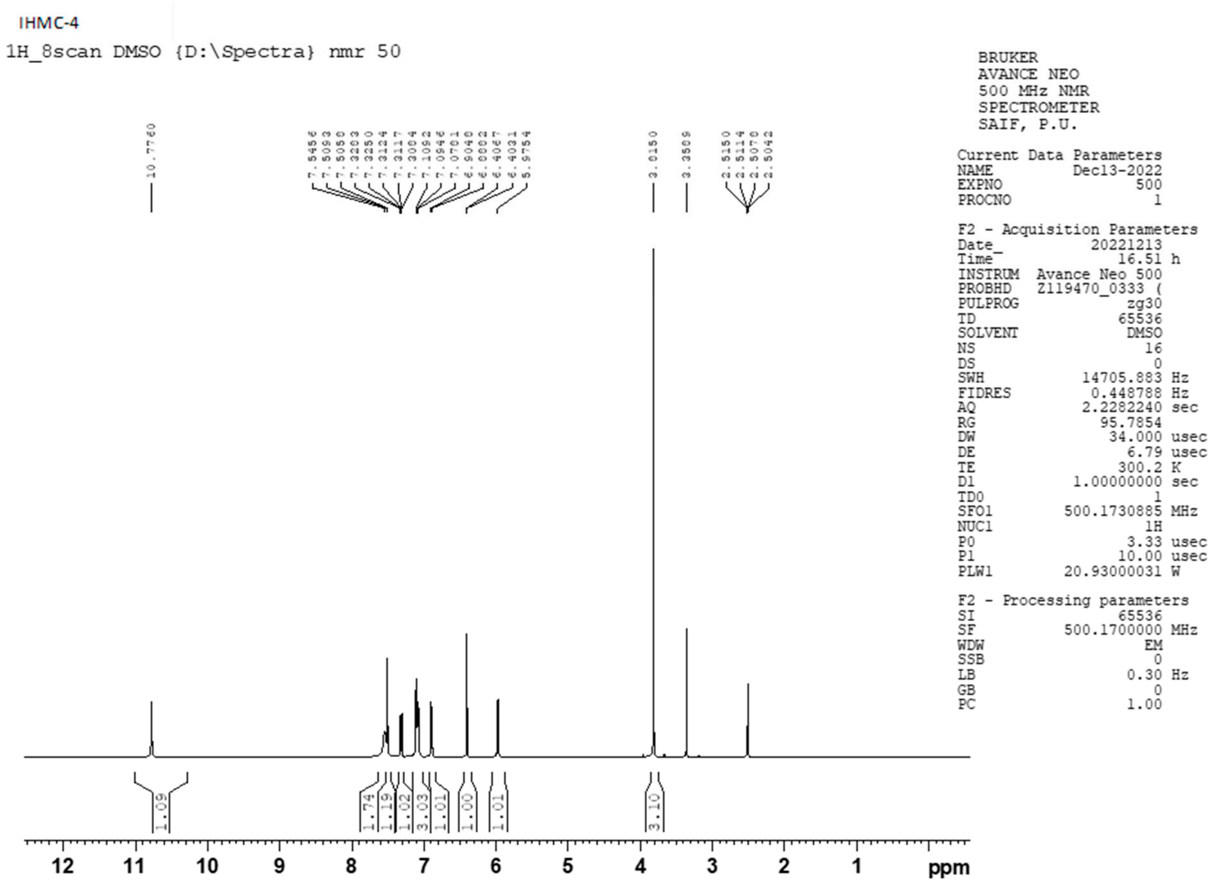

10.3.^{13}C NMR OF IHMC-4

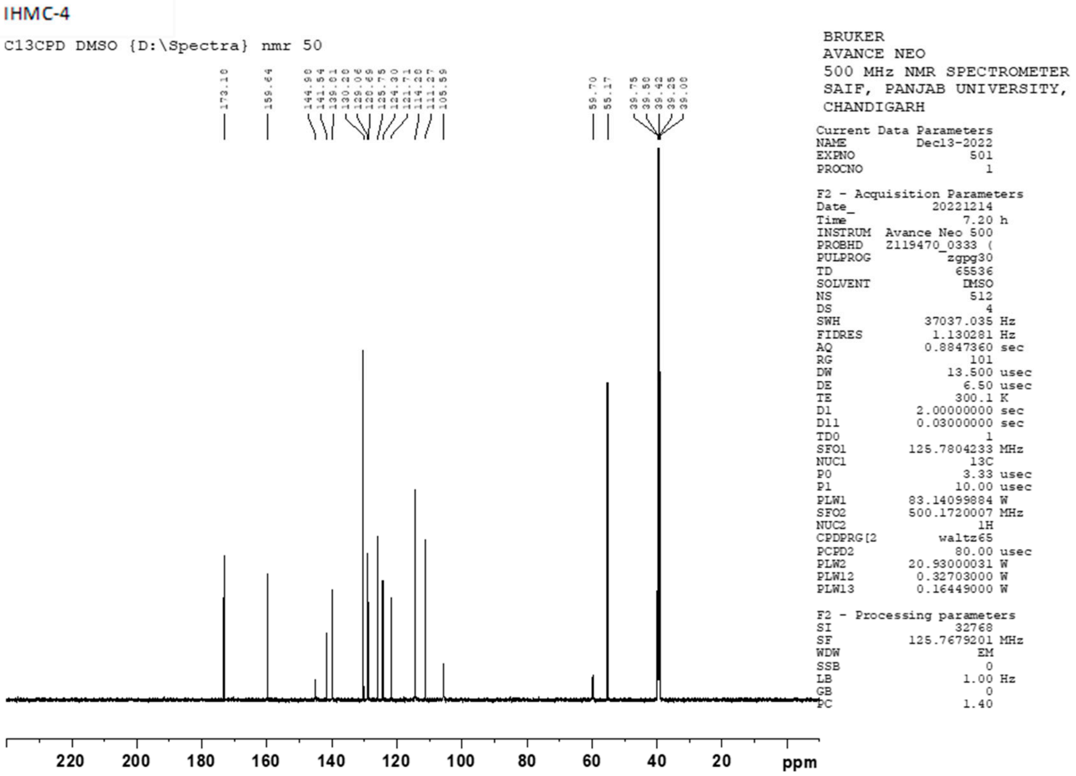

10.4.MASS SPECTRUM OF IHMC-4

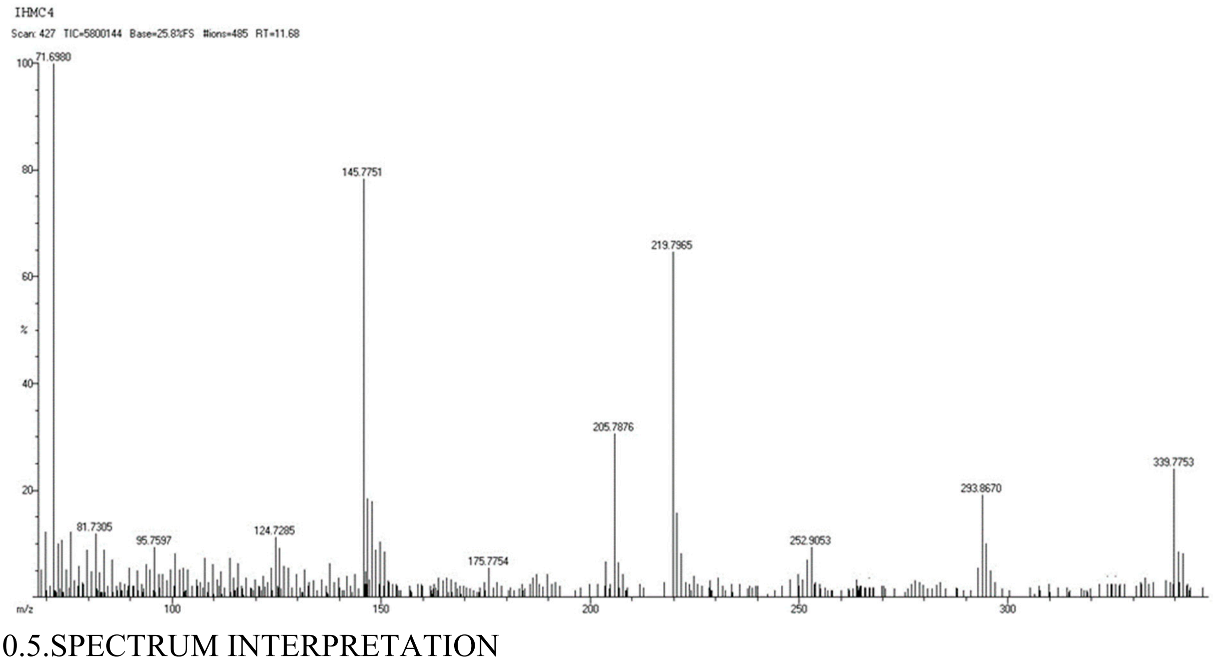

10.5.SPECTRUM INTERPRETATION

(3Z)-5-chloro-3-((3-(4-methoxyphenyl)allylidene)hydrazineylidene)indolin-2-one(IHMC-4) 1H NMR (500 MHz, DMSO) δ 10.78 (s,1H,NH), 7.55 – 7.01(m,7H,Ar-H), 6.90 (d,1H,=CH-), 6.40 (d,1H,-CH-)5.98 (s,1H,-CH-), 3.81 (s, 3H,-OCH3). 13C NMR (500 MHz, DMSO) δ 173.18, 159.64, 141.98, 139.81, 130.28, 129.06, 128.69, 125.75, 124.30, 121.71, 114.28, 111.27, 105.59, 59.70, 55.17. Molecular formula: $C_{18}H_{14}ClN_3O_2$ (HRMS) Calculated Mol.wt.=339.78, Observed Mol.wt.=339.7753.

Figure S11. IHMC-5 Structure

11. IHMC-5

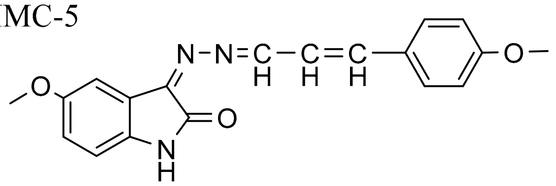

11.1 TLC OF IHMC-5

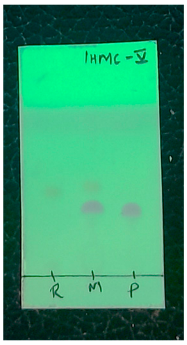

R = Reactant, M= Mixture, P = Product

11.2.^{1}H NMR OF IHMC-5

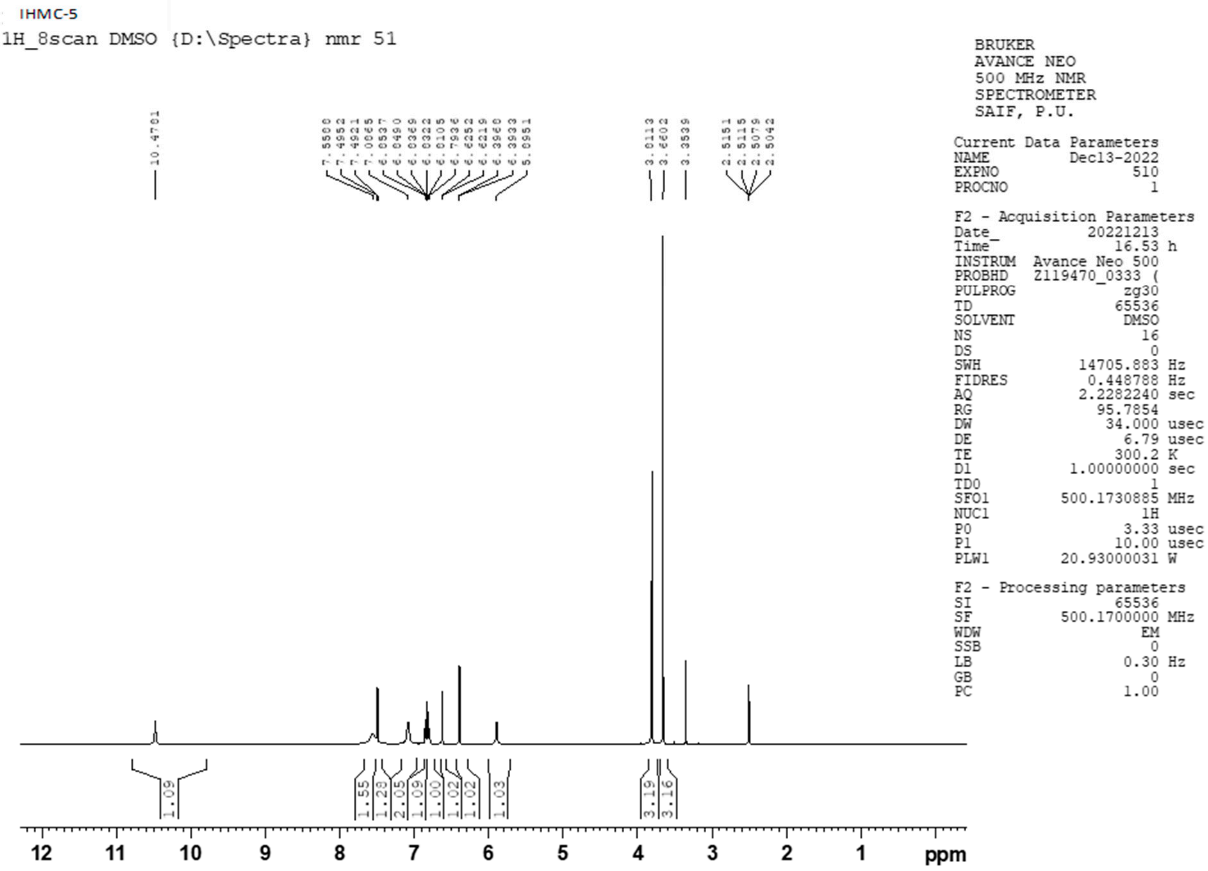

11.3.^{13}C NMR OF IHMC-5

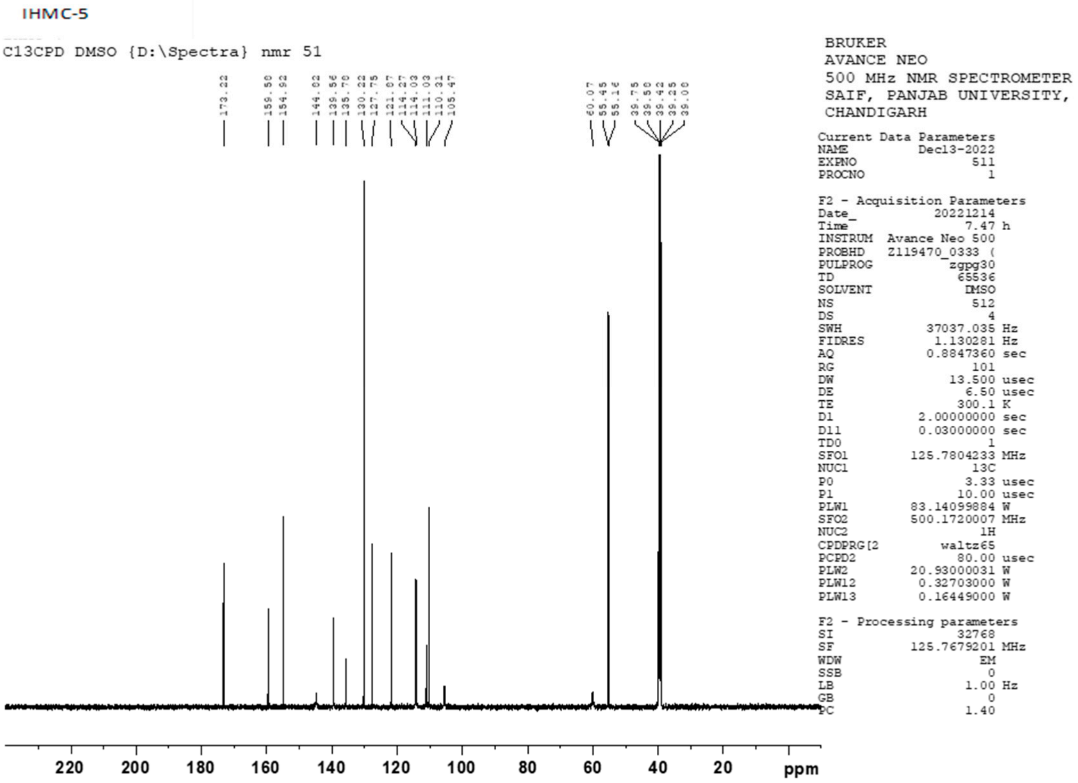

11.4.MASS SPECTRUM OF IHMC-5

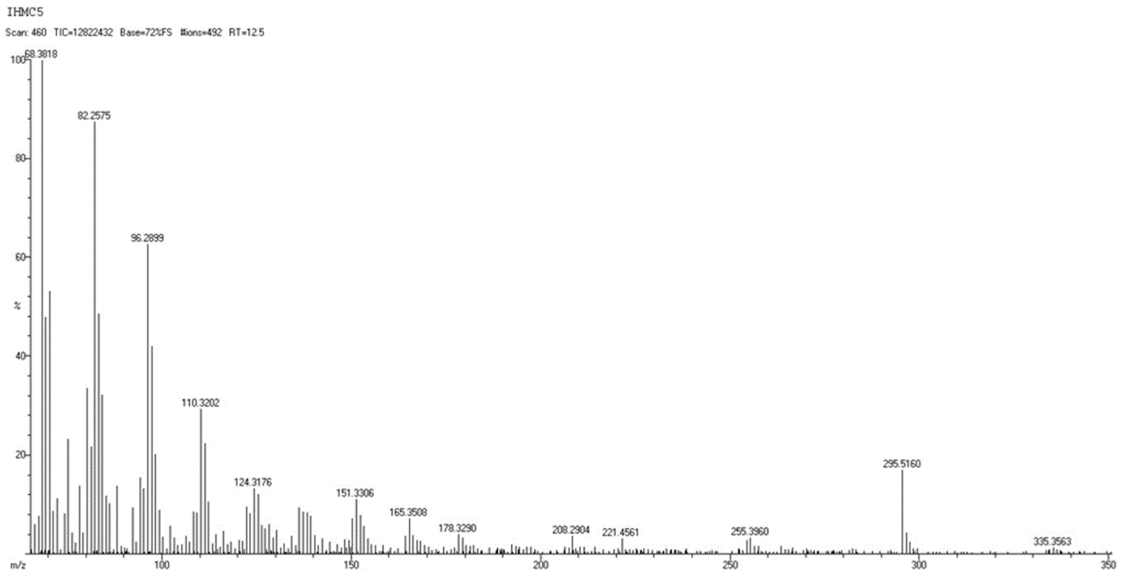

11.5.SPECTRUM INTERPRETATION

(3Z)-5-methoxy-3-((3-(4-methoxyphenyl)allylidene)hydrazineylidene)indolin-2-one(IHMC-5) 1H NMR (500 MHz, DMSO) δ 10.48 (s, 1H,NH), 7.58-6.62(m,7H,Ar-H), 6.78 (d,1H,=CH-), 6.40 (d,1H,-CH-), 5.90 (s, 1H,-CH-), 3.81 (s, 3H, -OCH3), 3.66 (s, 3H,-OCH3). 13C NMR (500 MHz, DMSO) δ 173.22, 159.58, 154.92, 144.82, 139.56, 135.78, 130.22, 127.75, 121.87, 114.27, 114.03, 111.03, 110.31, 105.47, 60.07, 55.45, 55.16. Molecular formula: C19H17N3O3 (HRMS) Calculated Mol.wt.=335.36, Observed Mol.wt.=335.3563

Figure S1. IHMC-6 Structure

12. IHMC-6

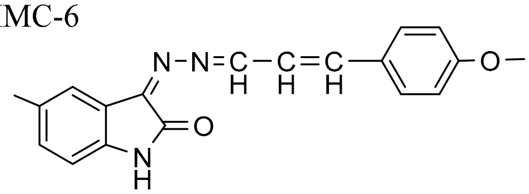

12.1 TLC OF IHMC-6

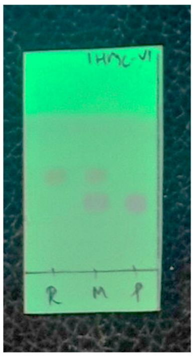

R = Reactant, M= Mixture, P = Product

12.2.^{1}H NMR OF IHMC-6

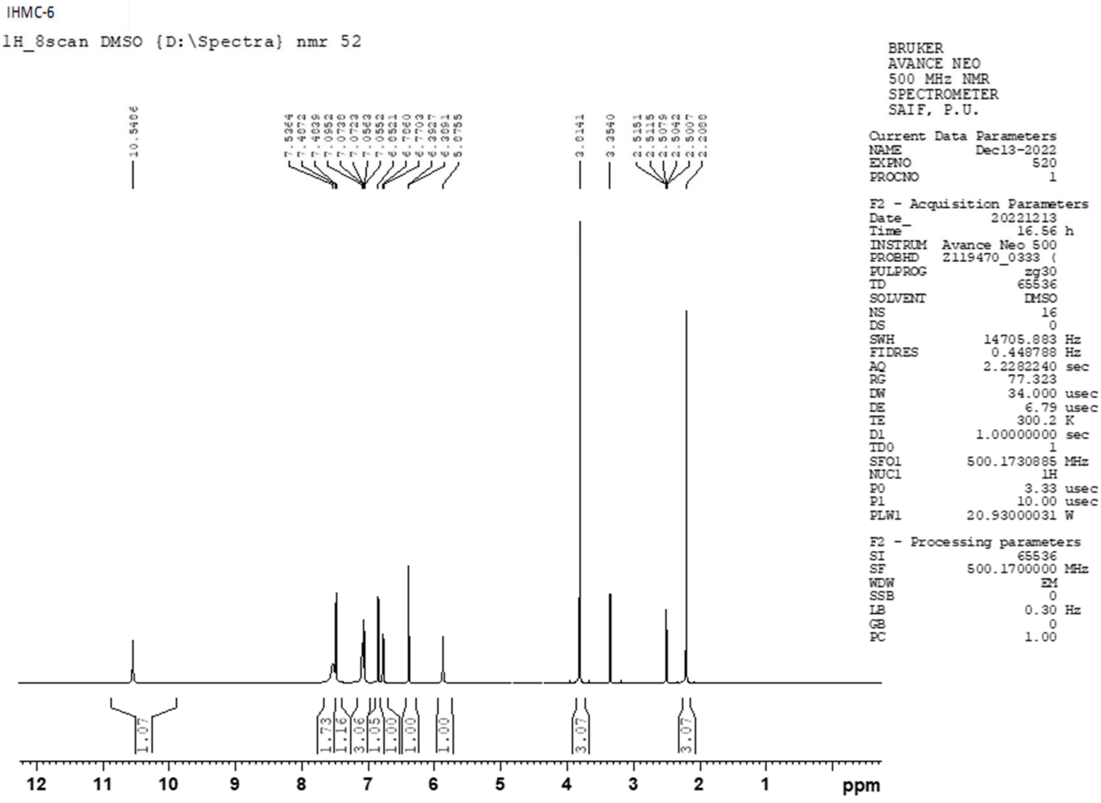

12.3.^{13}C NMR OF IHMC-6

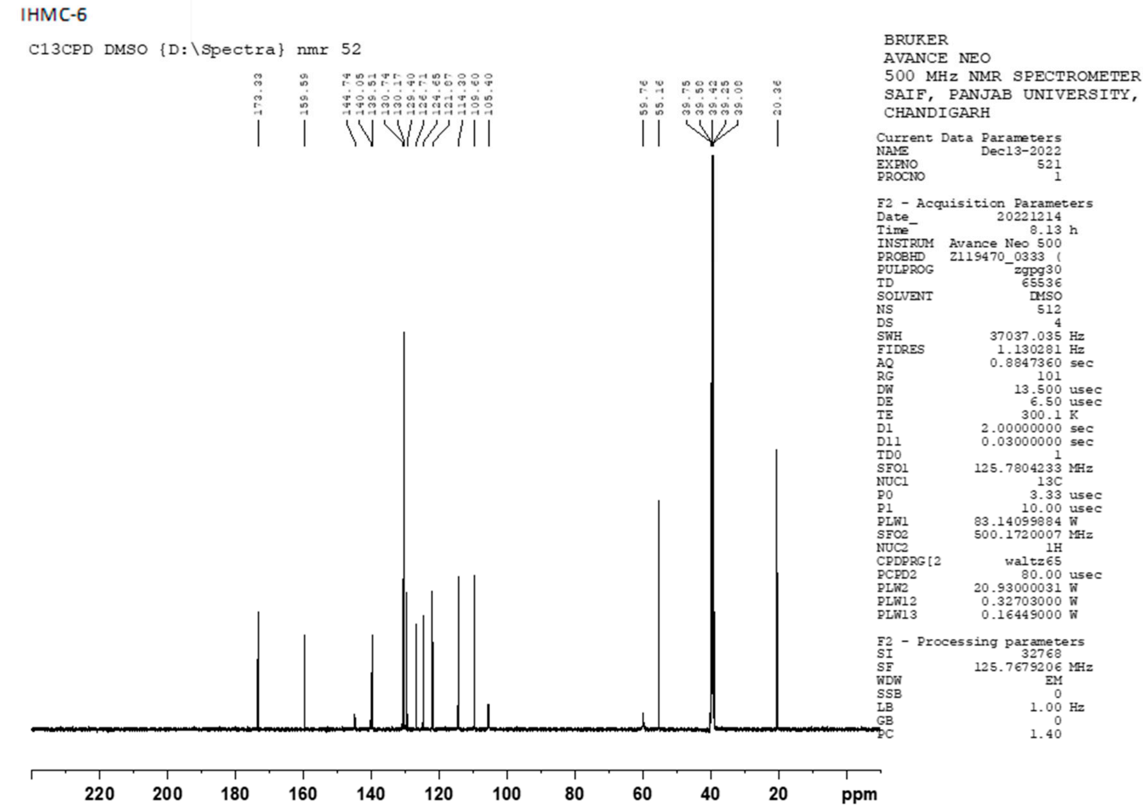

12.4.MASS SPECTRUM OF IHMC-6

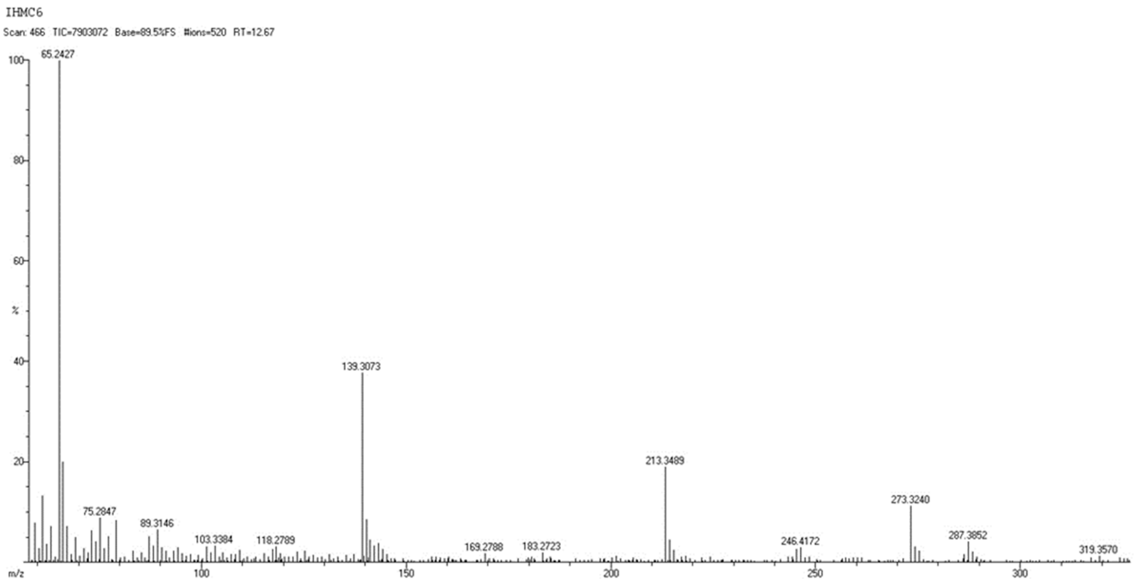

12.5.SPECTRUM INTERPRETATION

(3Z)-3-((3-(4-methoxyphenyl)allylidene)hydrazineylidene)-5-methylindolin-2-one(IHMC-6) 1H NMR (500 MHz, DMSO) δ 10.55 (s, 1H,NH), 7.52-6.85 (m, 7H,Ar-H), 6.78 (d,1H,=CH-), 6.39 (d,1H,-CH-), 5.88 (s, 1H,-CH-), 3.81 (s, 3H, -OCH3), 2.21 (s, 3H,-CH3). 13C NMR (500 MHz, DMSO) δ 173.33, 159.59, 144.74, 140.05, 139.51,130.74, 130.17,129.40,126.71, 124.65,121.87, 114.30,109.60, 105.40, 59.76, 55.16,20.36. Molecular formula: $C_{19}H_{17}N_3O_2$(HRMS) Calculated Mol.wt.= 319.36 Observed Mol.wt.= 319.3570.

Figure S13. IHNC-1 Structure

13. IHNC-1

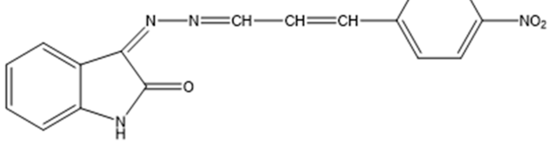

13.1 TLC OF IHNC-1

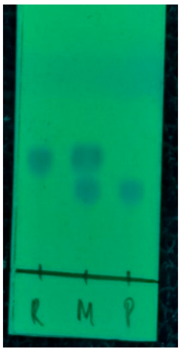

R = Reactant, M= Mixture, P = Product

13.2.^{1}H NMR OF IHNC-1

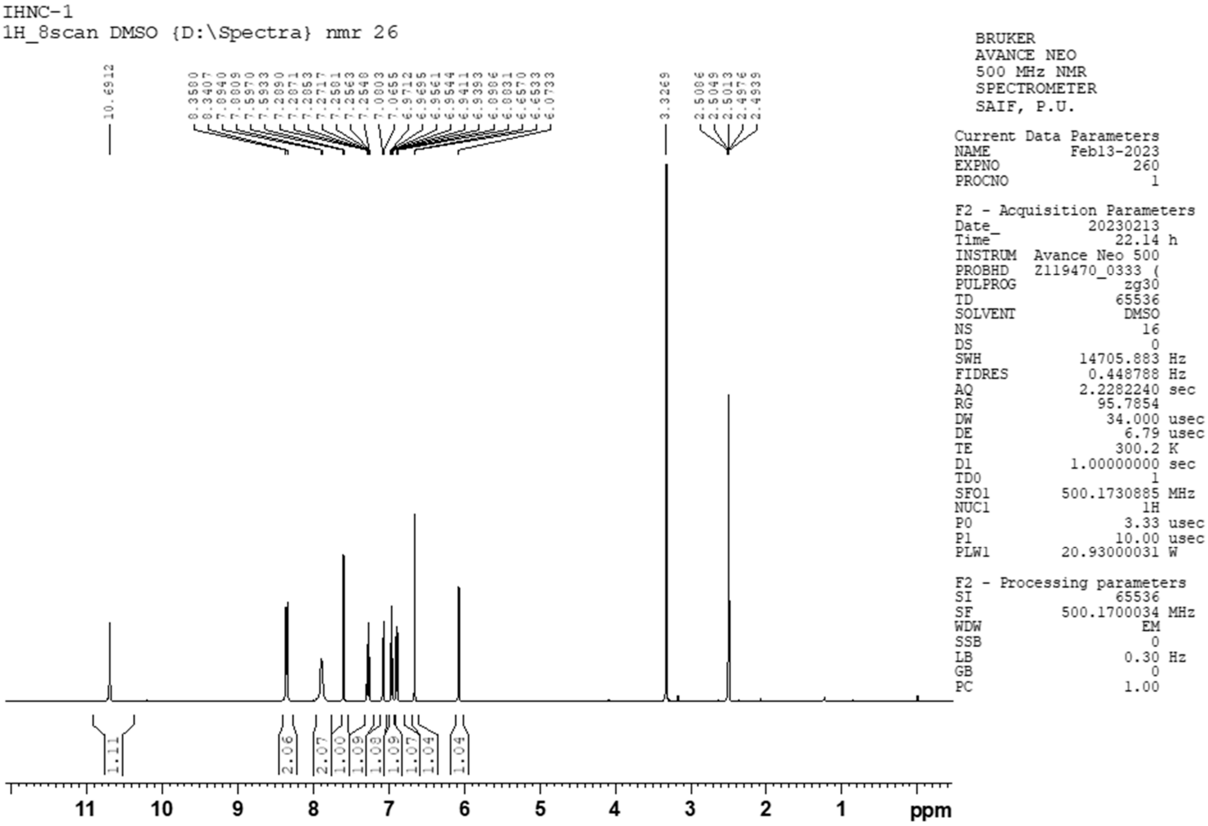

13.3.^{13}C NMR OF IHNC-1

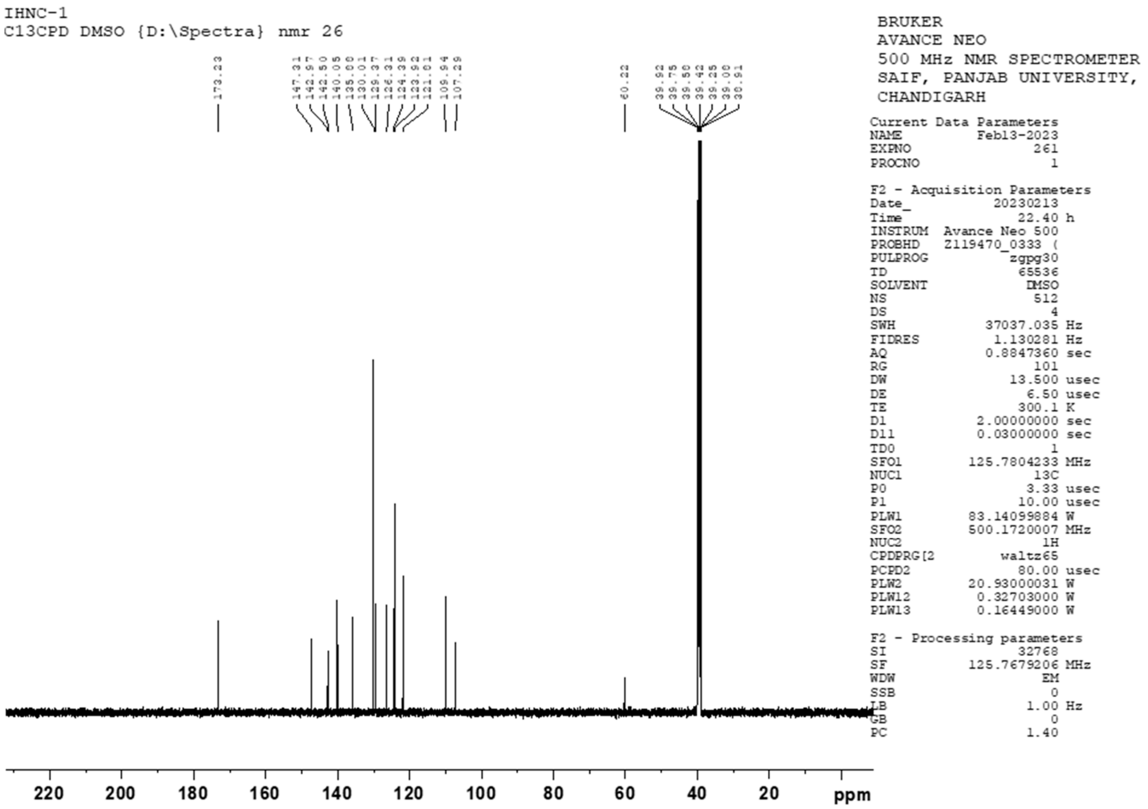

13.4.MASS SPECTRUM OF IHNC-1

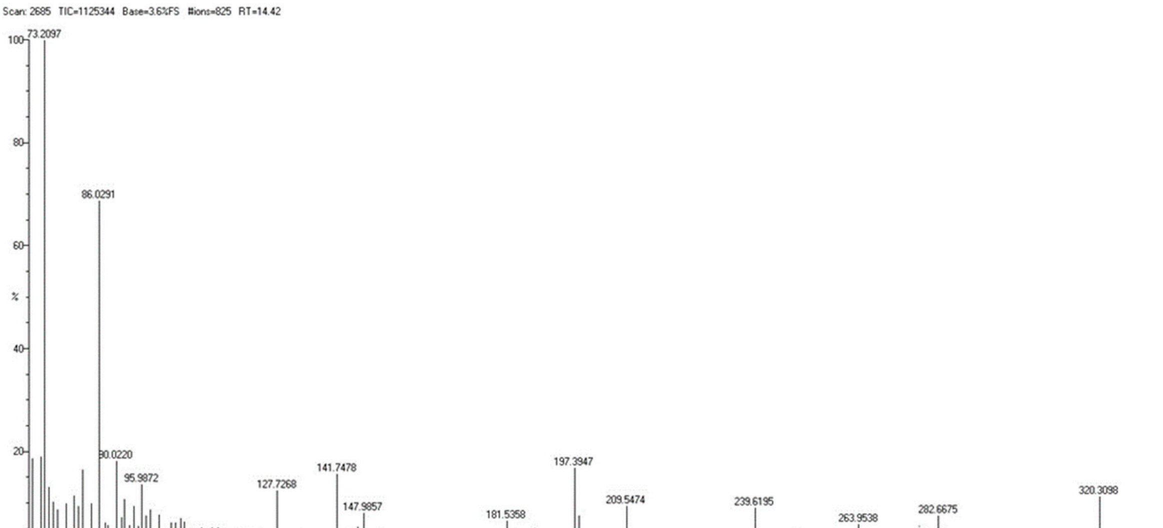

13.5. SPECTRUM INTERPRETATION

(3Z)-3-((3-(4-nitrophenyl)allylidene)hydrazineylidene)indolin-2-one(IHNC-1)^{1}H NMR (500 MHz, DMSO) δ 10.69 (s, 1H,NH), 8.35-6.95(m,8H,Ar-H), 6.89 (d,1H,=CH-), 6.66 (d,1H,-CH-), 6.07 (s, 1H,-CH-). ^{13}C NMR (500 MHz, DMSO) δ 173.23, 147.31, 142.97, 142.50, 140.05, 135.88, 130.01, 129.37, 126.31, 124.39, 123.92, 121.81, 109.94, 107.29, 60.22. Molecular formula: $C_{17}H_{12}N_4O_3$ (HRMS) Calculated Mol.wt. = 320.31 Observed Mol.wt. = 320.309

Figure S14. IHNC-2 Structure

14. IHNC-2

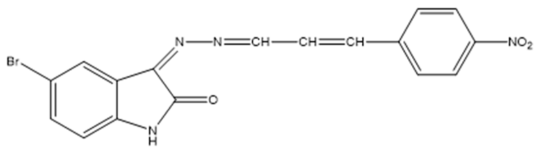

14.1 TLC OF IHNC-2

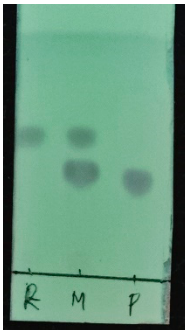

R = Reactant, M= Co-mixture, P = Product

14.2.^{1}H NMR OF IHNC-2

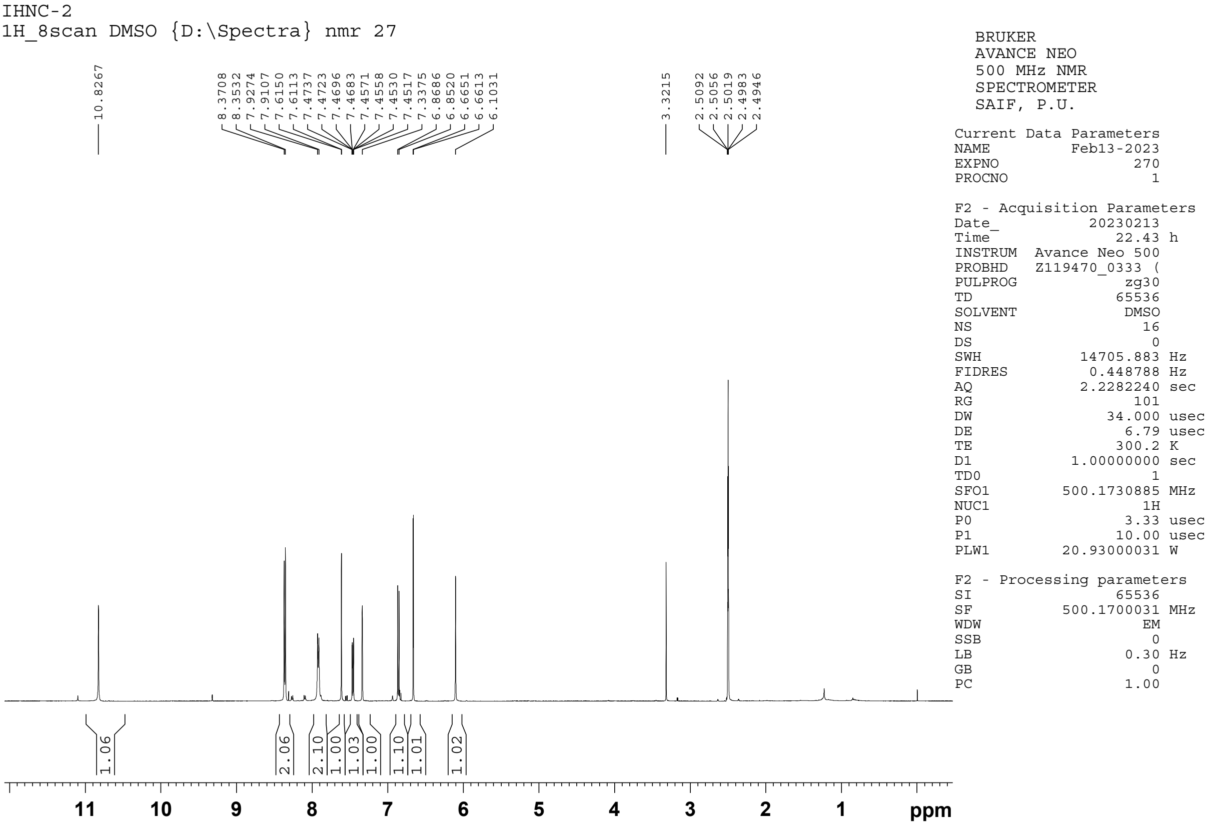

14.3.^{13}C NMR OF IHNC-2

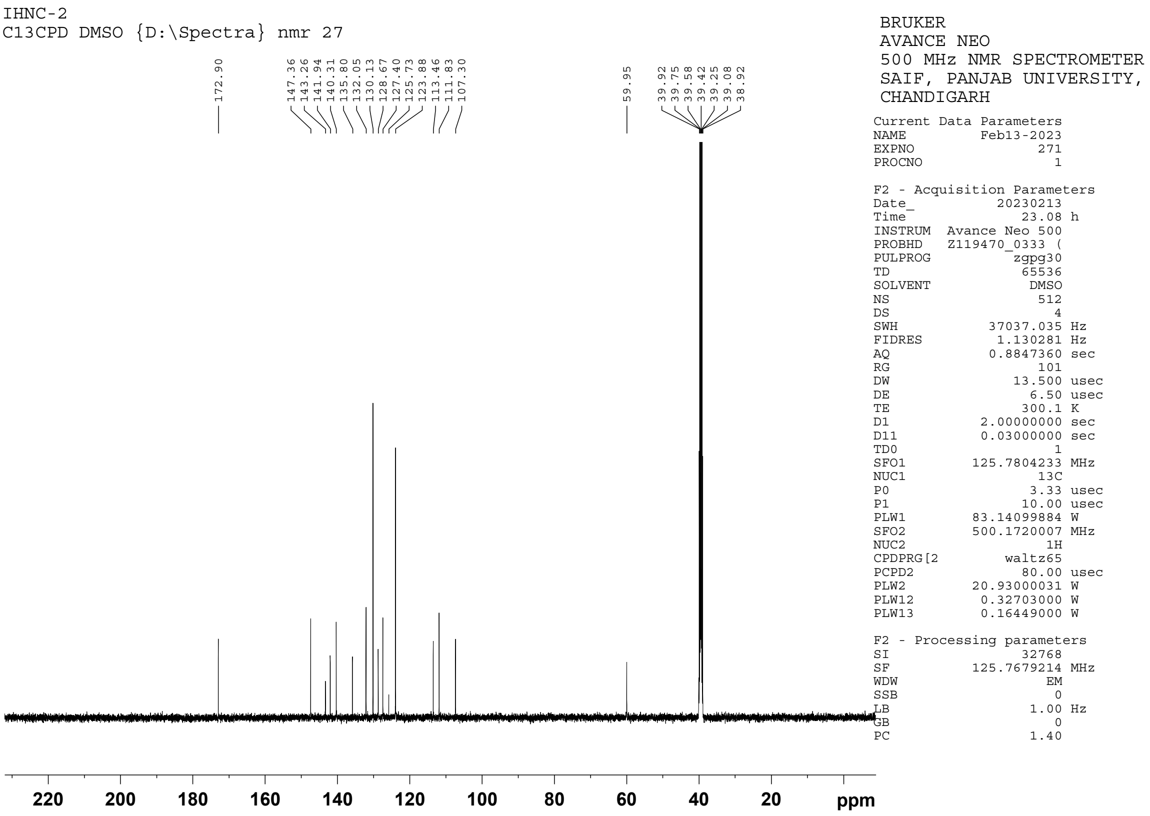

14.4. MASS SPECTRUM OF IHNC-2

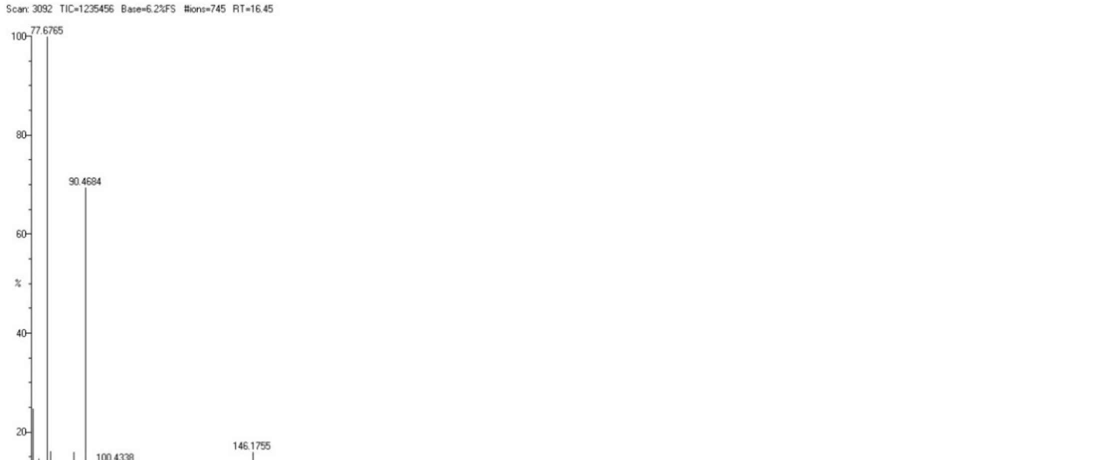

14.5.SPECTRUM INTERPRETATION

(3Z)-5-bromo-3-((3-(4-nitrophenyl)allylidene)hydrazineylidene)indolin-2-one(IHNC-2)^{1}H NMR (500 MHz, DMSO) δ 10.83 (s, 1H,NH), 8.36-7.34(m, 7H,Ar-H), 6.96 (d,1H,=CH-), 6.66 (d,1H,-CH-), 6.10 (s, 1H,-CH-). ^{13}C NMR (500 MHz, DMSO) δ 172.90, 147.36, 143.26, 140.31, 135.80, 132.05, 130.13, 128.67, 127.40, 125.73, 123.88, 113.46, 111.83, 107.30, 59.95. Molecular formula: $C_{17}H_{11}BrN_4O_3$(HRMS) Calculated Mol.wt.= 399.20 Observed Mol.wt. = 399.199

Figure S15. IHNC-3 Structure

15. IHNC-3

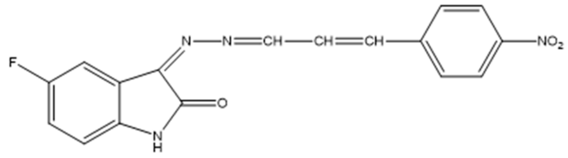

15.1 TLC OF IHNC-3

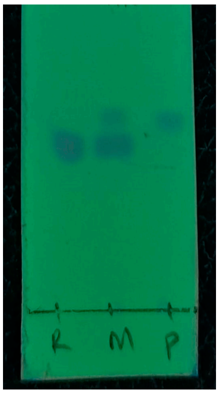

R = Reactant, M= Co-mixture, P = Product

15.2.^{1}H NMR OF IHNC-3

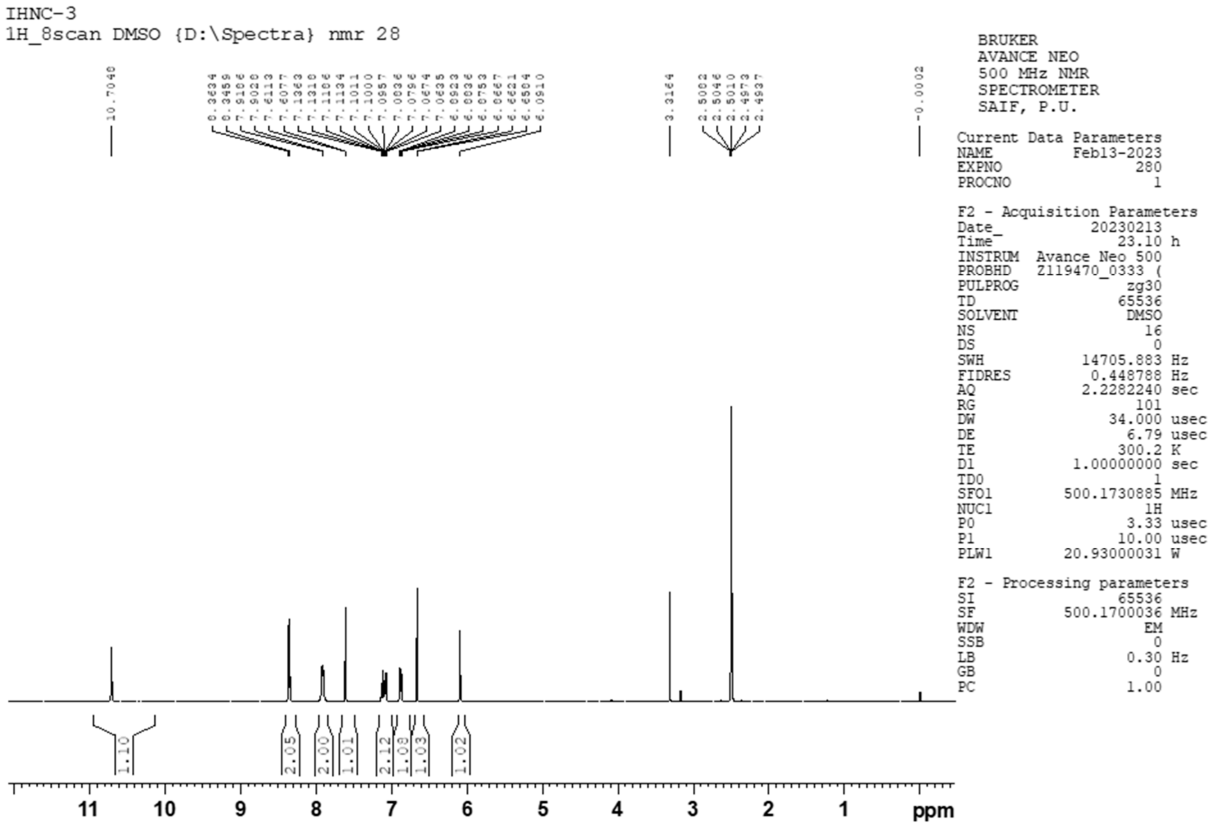

15.3.^{13}C NMR OF IHNC-3

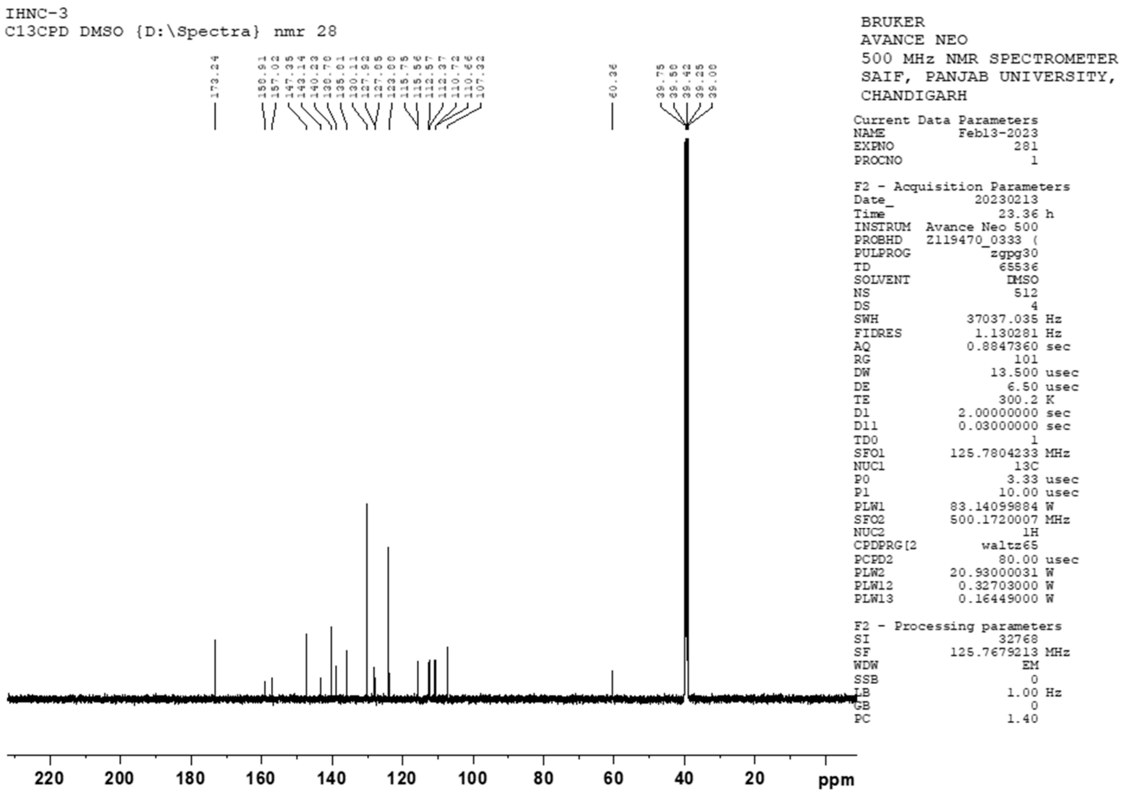

15.4.MASS SPECTRUM OF IHNC-3

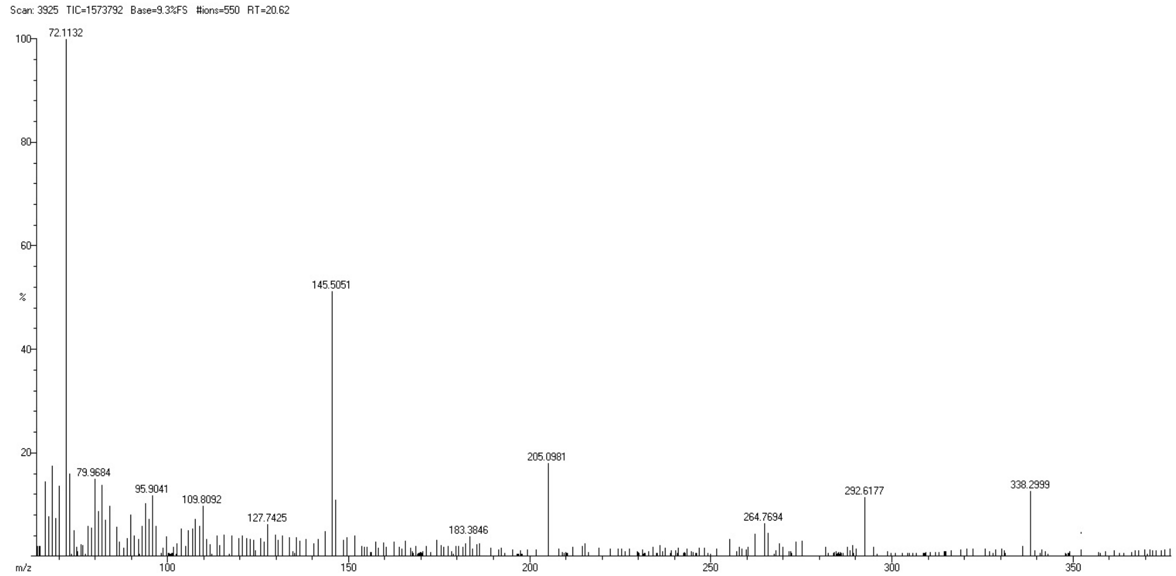

15.5.SPECTRUM INTERPRETATION

(3Z)-5-fluoro-3-((3-(4-nitrophenyl)allylidene)hydrazineylidene)indolin-2-one(IHNC-3) ^{1}H NMR (500 MHz, DMSO) δ 10.70 (s, 1H,NH), 8.35-6.81(m,8H,Ar-H,=CH) 6.66 (d,1H,-CH-), 6.09 (s, 1H,-CH-).) ^{13}C NMR (500 MHz, DMSO) δ173.24, 158.91, 157.02, 147.35, 143.14, 140.23, 138.78, 135.81, 130.11, 127.92, 123.88, 115.75, 112.57, 112.37, 110.72, 107.32, 60.36.Molecular formula: $C_{17}H_{11}FN_4O_3$(HRMS) Calculated Mol.wt. = 338.30 Observed Mol.wt. = 338.299

Figure S16. IHNC-4 Structure

16. IHNC-4

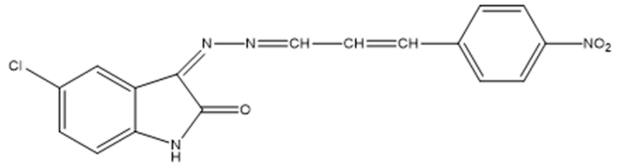

16.1 TLC OF IHNC-4

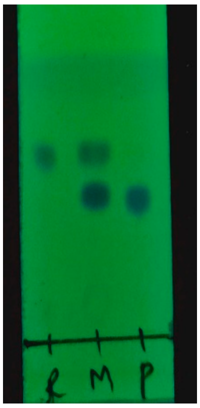

R = Reactant, M= Mixture, P = Product

16.2.^{1}H NMR OF IHNC-4

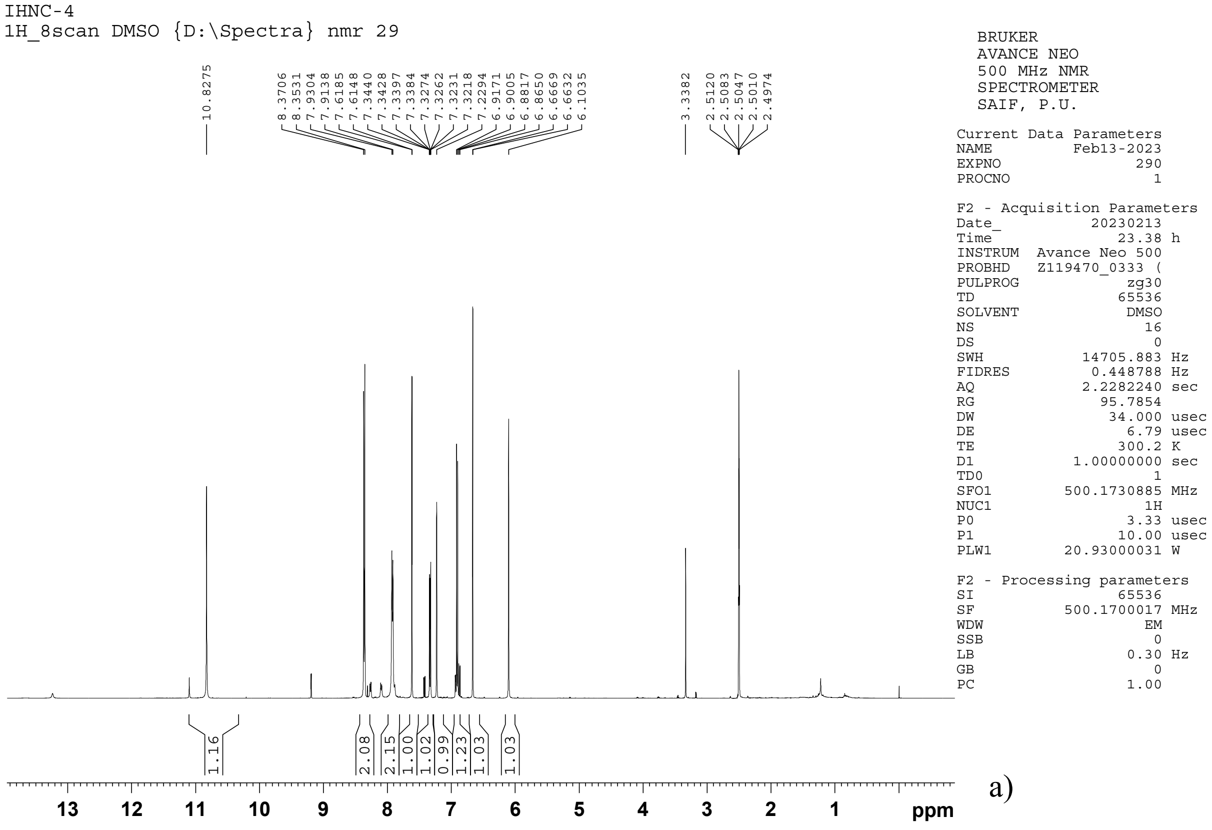

a)

16.3.^{13}C NMR OF IHNC-4

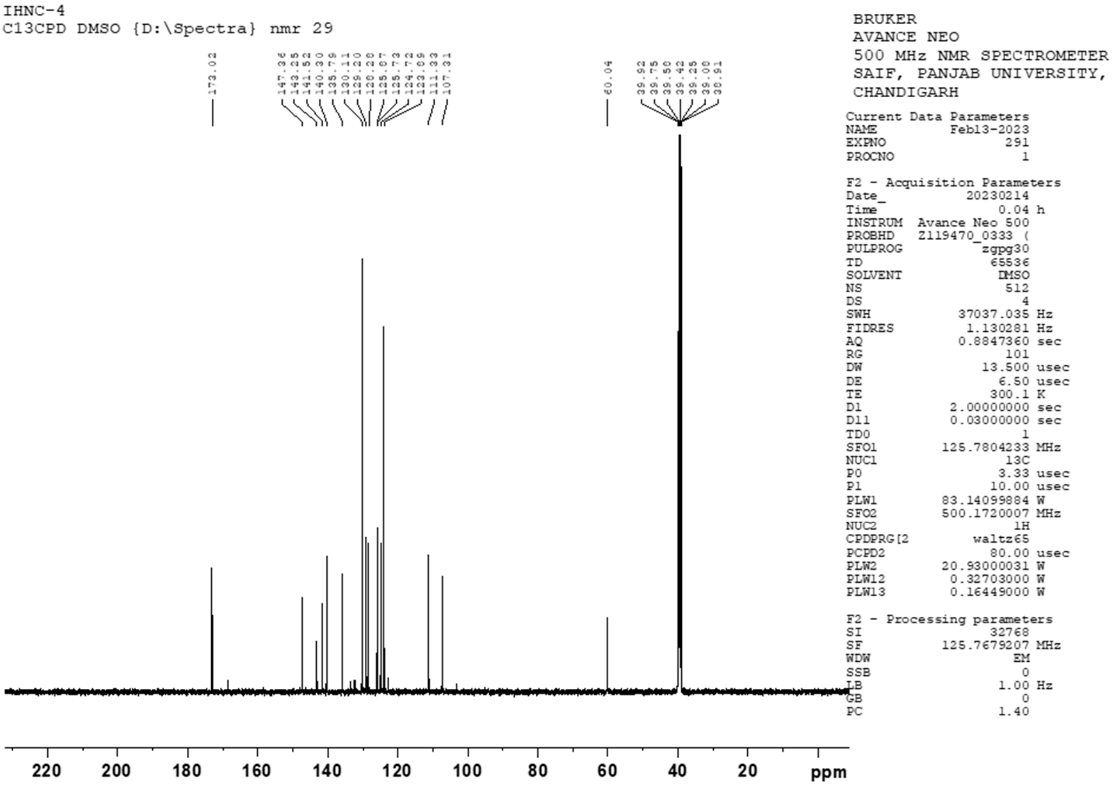

16.4.MASS SPECTRUM OF IHNC-4

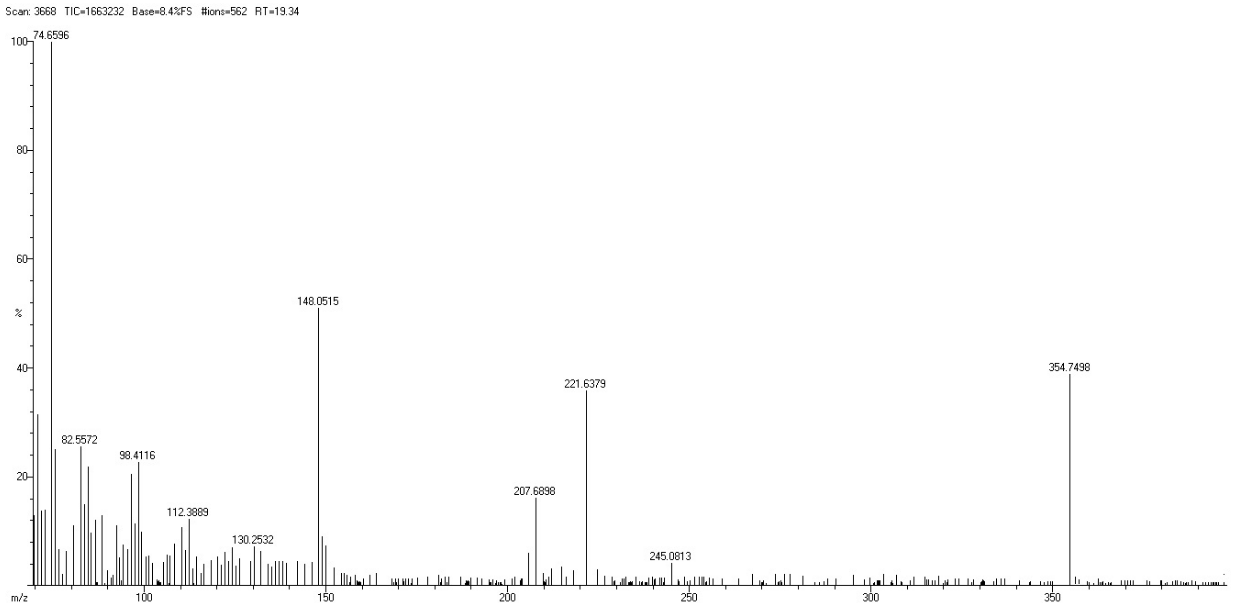

16.5.SPECTRUM INTERPRETATION

(3Z)-5-chloro-3-((3-(4-nitrophenyl)allylidene)hydrazineylidene)indolin-2-one(IHNC-4) ^{1}H NMR (500 MHz, DMSO) δ 10.83 (s, 1H,NH), 8.36 (m,8H,Ar-H,=CH-), 6.67 (d,1H,-CH-), 6.10 (s,1H,-CH-) ^{13} C NMR (500 MHz, DMSO) δ 173.02, 147.36, 143.25, 141.30, 140.30, 135.79, 130.11, 129.20, 128.20, 128.28, 125.87, 125.73, 124.72, 123.89, 111.33, 107.31, 60.04. Molecular formula: $C_{17}H_{11}ClN_4O_3$(HRMS) Calculated Mol.wt. = 354.75 Observed Mol.wt. = 354.749

Figure S17. IHNC-17 Structure

17. IHNC-5

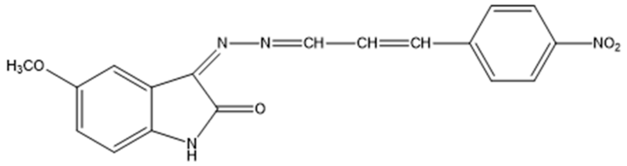

17.1 TLC OF IHNC-5

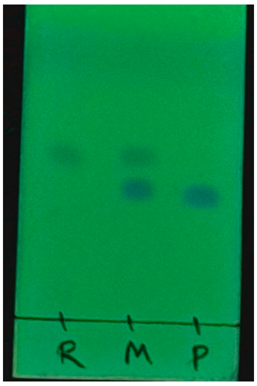

R = Reactant, M= Mixture, P = Product

17.2.^{1}H NMR OF IHNC-5

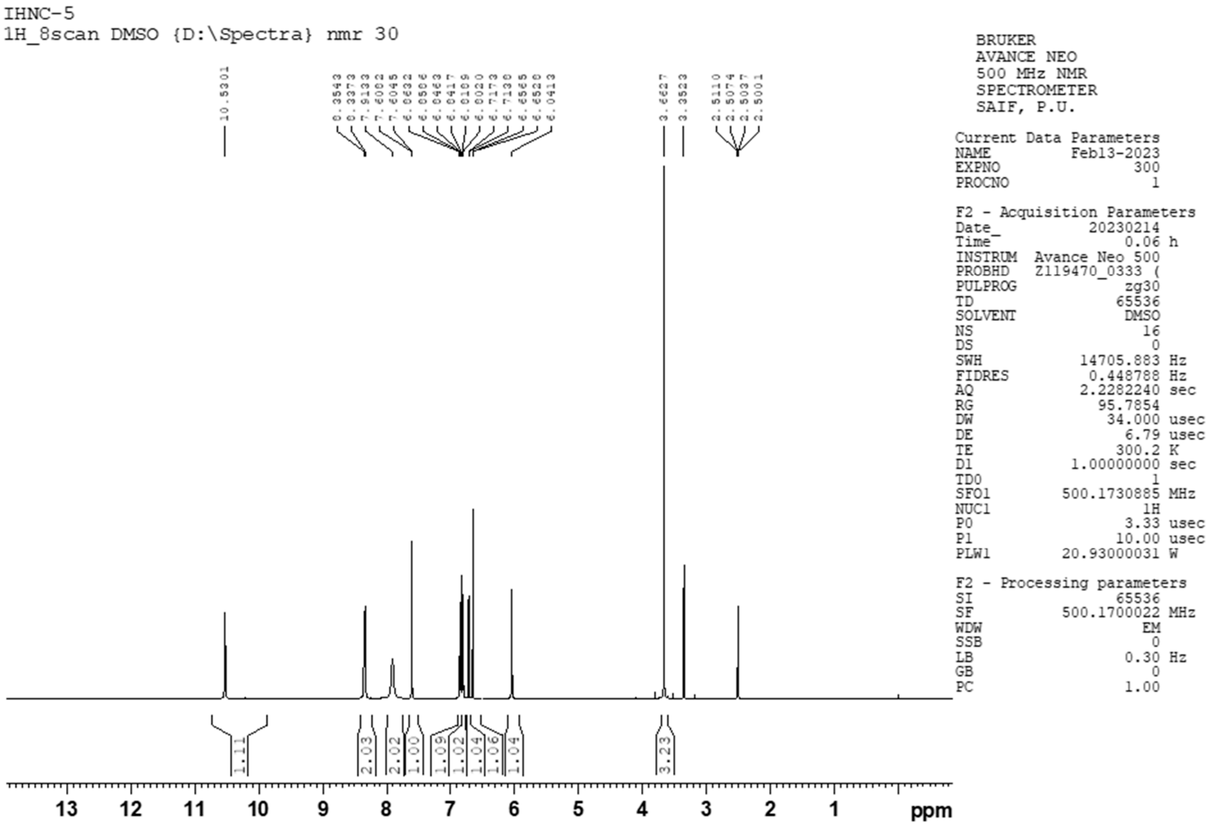

17.3.^{13}C NMR OF IHNC-5

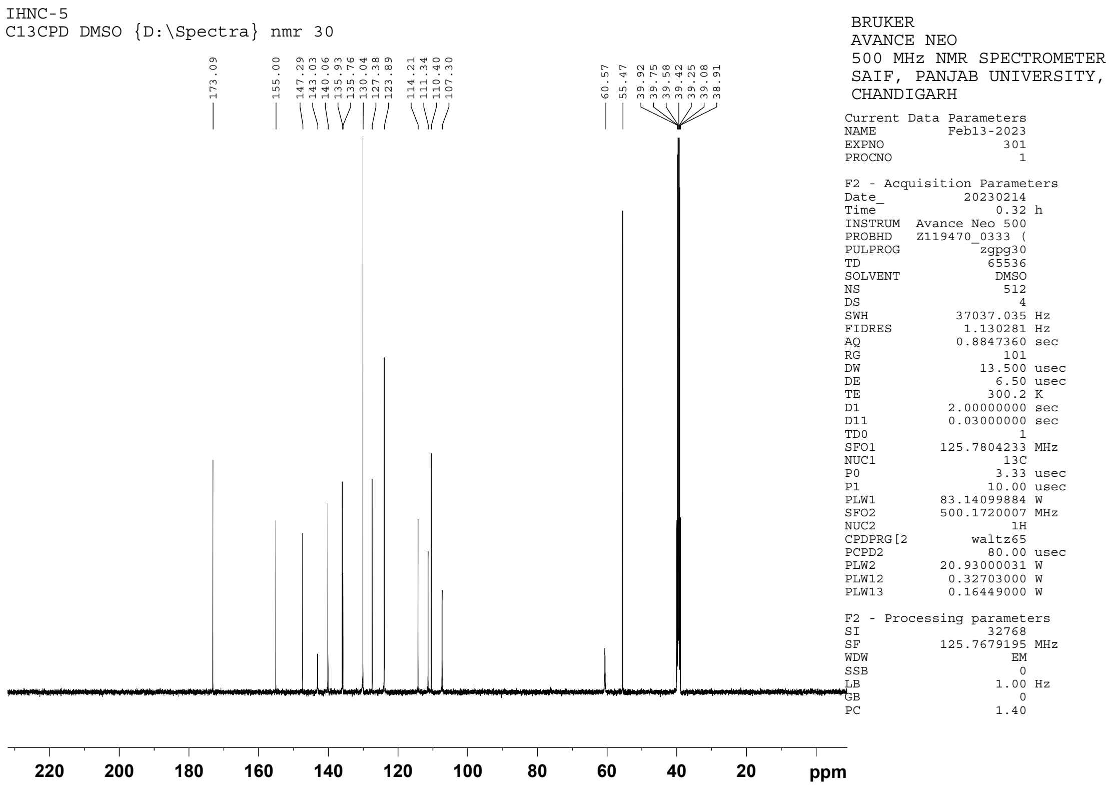

17.4.MASS SPECTRUM OF IHNC-5

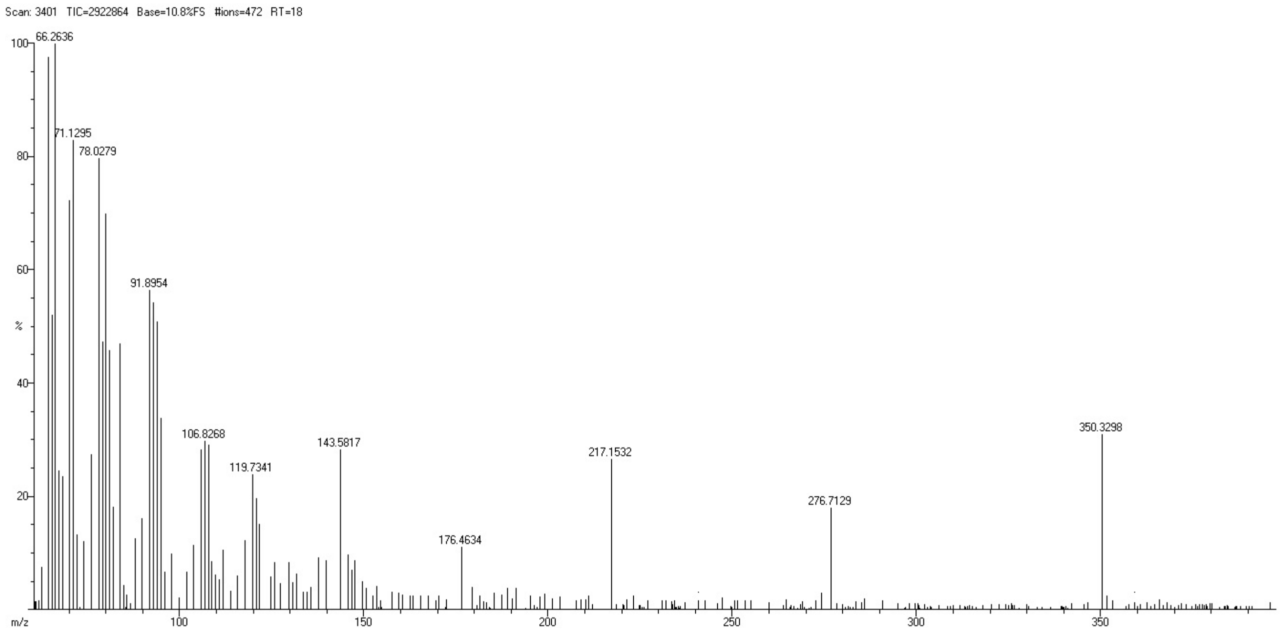

17.5.SPECTRUM INTERPRETATION

(3Z)-5-methoxy-3-((3-(4-nitrophenyl)allylidene)hydrazineylidene)indolin-2-one(IHNC-5) ^{1}H NMR (500 MHz, DMSO) δ 10.53 (s, 1H,NH), 8.33-6.72 (m,7H,Ar-H), 6.81 (d,1H,=CH-), 6.65 (d,1H,-CH-), 6.04 (s, 1H,-CH-), 3.66 (s, 3H,-OCH_3). ^{13}C NMR (500 MHz, DMSO) δ 173.09, 155, 147.29, 143.03, 140.06, 135.93, 135.76, 130.04, 127.38, 123.84, 114.21, 111.34, 110.40, 107.30, 60.57, 55.47. Molecular formula: $C_{18}H_{14}N_4O_4$(HRMS) Calculated Mol.wt. = 350.33 Observed Mol.wt. = 350.329

Figure S18. IHNC-6 Structure

18. IHNC-6

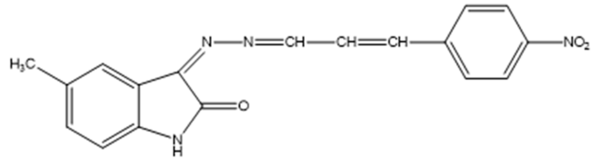

18.1 TLC OF IHNC-6

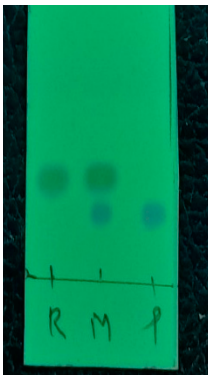

R = Reactant, M= Mixture, P = Product

18.2.^{1}H NMR OF IHNC-6

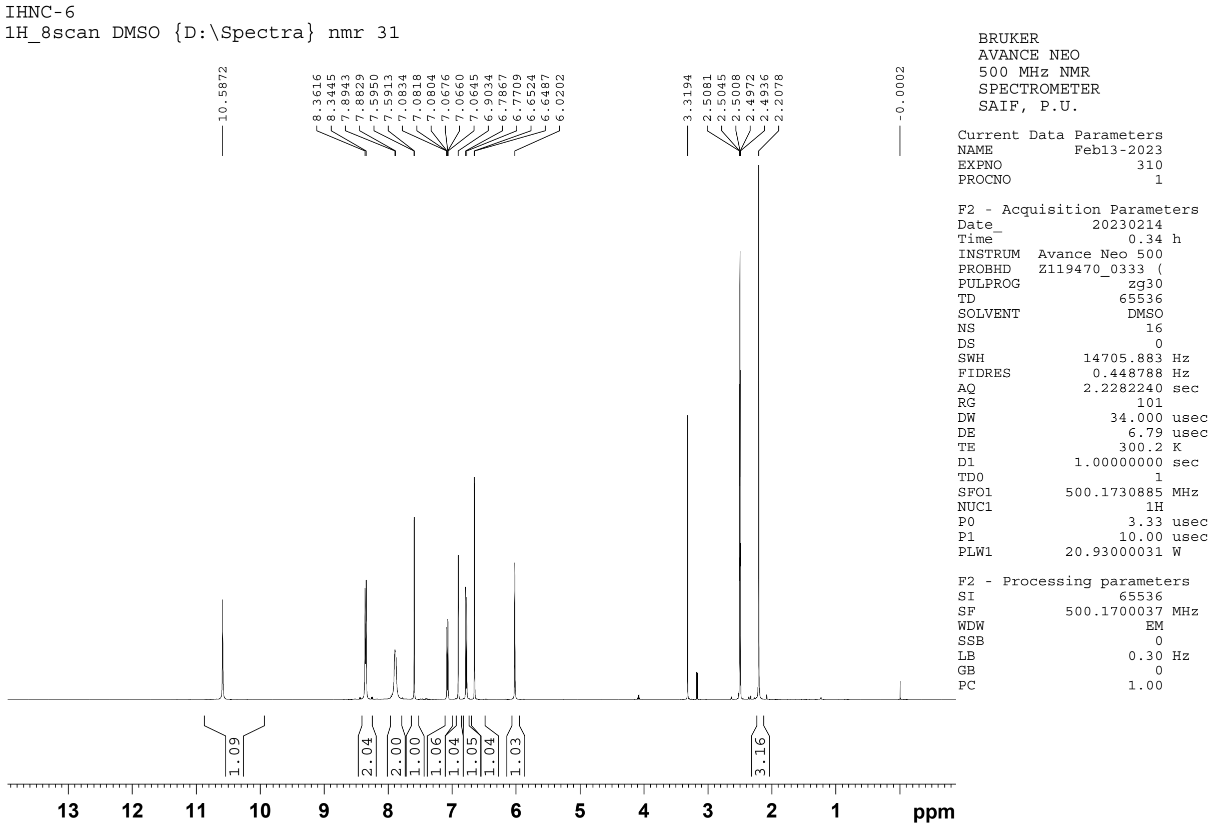

18.3.^{13}C NMR OF IHNC-6

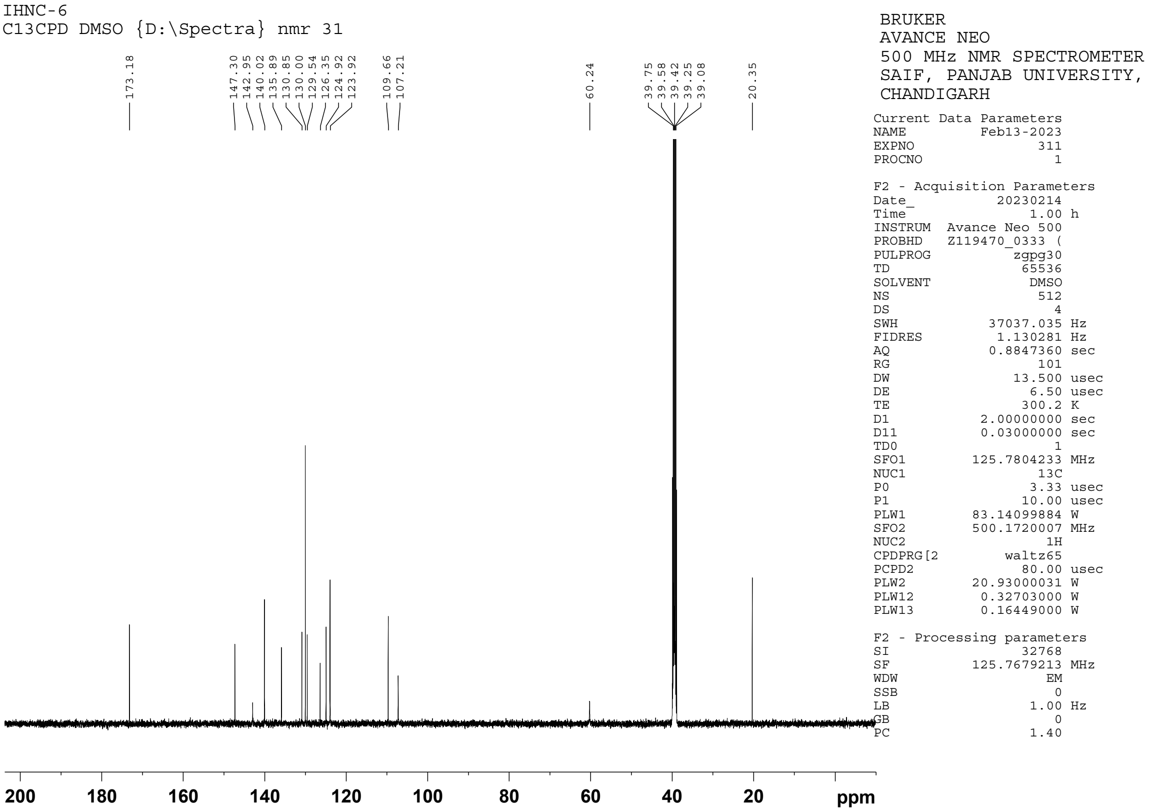

18.4.MASS SPECTRUM OF IHNC-6

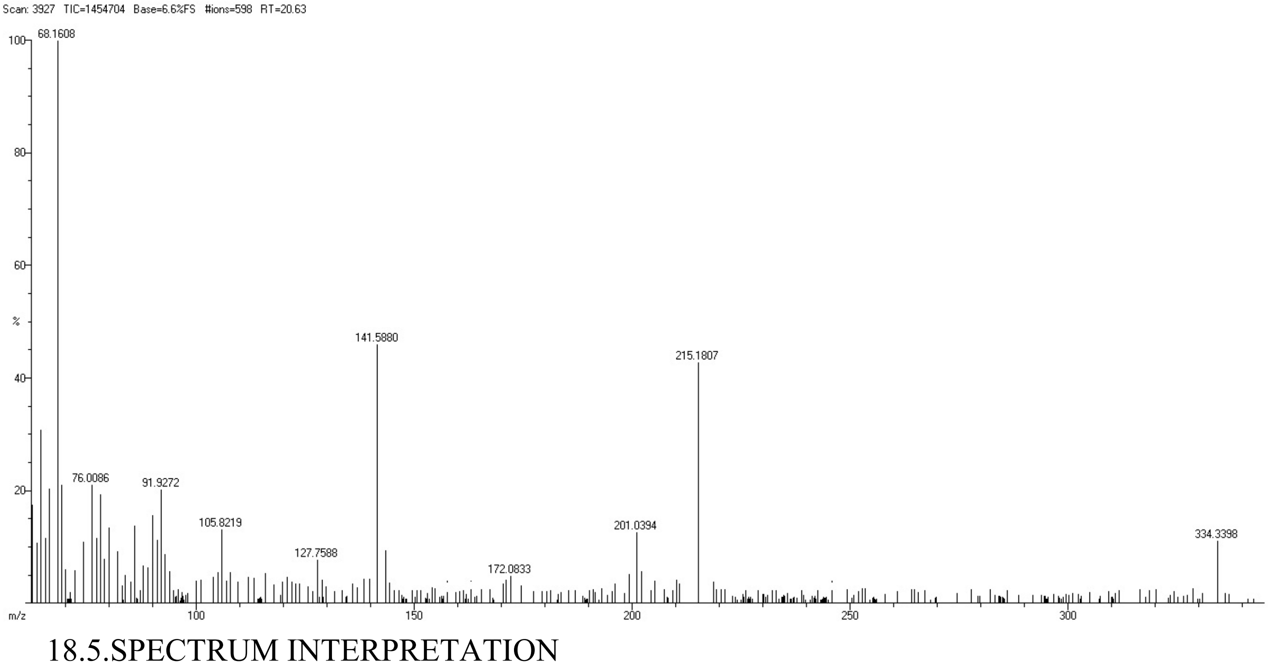

18.5.SPECTRUM INTERPRETATION

(3Z)-5-methyl-3-((3-(4-nitrophenyl)allylidene)hydrazineylidene)indolin-2-one(IHNC-6)^{1}H NMR (500 MHz, DMSO) δ 10.59 (s, 1H,NH), 8.32 (m, 7H,Ar-H), 6.78 (d,1H,=CH-), 6.65 (d,1H,-CH-), 6.02 (s, 1H,-CH-), 2.21 (s, 3H,-CH_3). ^{13}C NMR (500 MHz, DMSO) δ 173.18, 147.30, 142.95, 140.02, 135.89, 130.85, 130, 129.54, 126.35, 124.92, 123.92, 109.66, 107.21, 60.24, 20.35. Molecular Formula: $C_{18}H_{14}N_4O_3$(HRMS) Calculated Mol.wt. = 334.34 Observed Mol.wt. = 334.339.

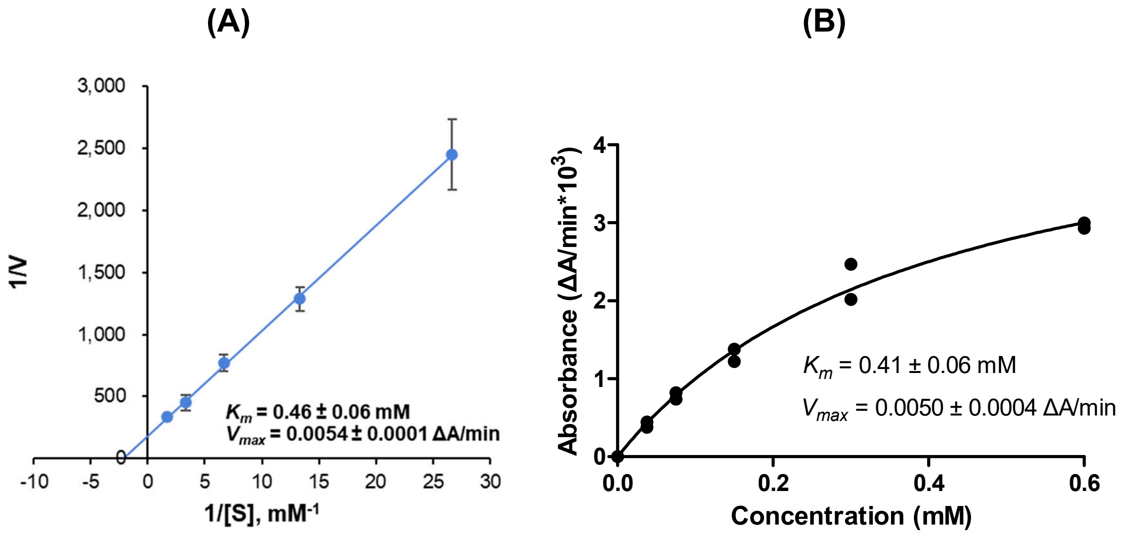

Figure S19. Lineweaver-Burk plot (A) and nonlinear regression of Michaelis-Menten equation analyzed by Prism 5.0 (B) for MAO-B kinetics.
